# Temporal trends and geographic disparities in thyroid cancer burden: a global analysis from 1990 to 2021

**DOI:** 10.3389/fnut.2025.1613737

**Published:** 2025-07-16

**Authors:** Zuzhi Zhao, Yinghao Fan, Peng Sun, Suqin Zhang, Mengfei Xu, Jianhua Li, Pengfei Xu

**Affiliations:** ^1^Department of Thyroid Surgery, The First Affiliated Hospital of Zhengzhou University, Zhengzhou, China; ^2^The Children's Hospital, The First Affiliated Hospital of Zhengzhou University, Zhengzhou, China; ^3^Clinical Systems Biology Laboratories, Translational Medicine Center, The First Affiliated Hospital of Zhengzhou University, Zhengzhou, China

**Keywords:** thyroid cancer, global burden of disease, disability-adjusted life years, incidence, prevalence

## Abstract

**Background:**

Over the past 40 years, the global incidence of thyroid cancer has increased steadily. This study aimed to update the evaluation of thyroid cancer prevalence, incidence, mortality, and Disability–Adjusted Life Years (DALYs) rates from 1990 to 2021, with a focus on integrating prevalence data. Analyses were stratified by gender, age, and Socio–Demographic Index (SDI) at global, regional, and national levels.

**Methods:**

Data were obtained from the 2021 Global Burden of Disease, Injuries, and Risk Factors Study (GBD). The estimated annual percentage change (EAPC) was calculated to quantify temporal trends and evaluate age - standardized rates for prevalence (ASPR), incidence (ASIR), mortality (ASDR), and DALYs.

**Results:**

In 2021, the global thyroid cancer burden was substantial, with 1,987,148.5 cases. From 1990 to 2021, the ASPR increased from 14.9 (95% Uncertainty Interval [UI]: 14.1–16.0) to 23.1 (95% UI: 20.7–25.6) per 100,000 population, with an EAPC of 1.58 (95% UI: 1.44–1.73); the ASIR increased from 2.1 (95% UI: 2–2.2) to 2.9 (95% UI: 2.6–3.2) per 100,000 population, with an EAPC of 1.25 (95% UI: 1.14–1.37); the ASDR declined from 0.6 (95% UI: 0.5–0.6) to 0.5 (95% UI: 0.5–0.6) per 100,000 population, with an EAPC of −0.24 (95% UI: −0.24 – −0.21); the age - standardized DALY rate decreased from 15.2 (95% UI: 14.2–16.8) to 14.6 (95% UI: 12.8–16.1) per 100,000 population, with an EAPC of −0.14 (95% UI: −0.17 – –0.11). Western Sub–Saharan Africa had the lowest rates, while high–income North America had the highest ASPR and ASIR, and Andean Latin America had the highest ASDR. Higher SDI regions showed higher ASPR and ASIR, whereas lower SDI regions had higher ASDR. Saudi Arabia had the highest ASPR and ASIR, and Ethiopia had the highest ASDR and age–standardized DALY rate.

**Conclusion:**

From 1990 to 2021, the global health burden of thyroid cancer increased significantly, with marked geographical disparities. Prevention and control strategies should consider the unequal global distribution of the disease.

## Introduction

In recent decades, thyroid cancer has emerged as a pressing global health concern, casting a wide-reaching shadow over public health (1). It affects individuals across all age spectrums, genders, and geographical boundaries (2). The disease’s impact is not only limited to the physical well-being of patients but also extends to their mental health, quality of life, and the economic burden on healthcare systems globally. From developed countries to developing nations, thyroid cancer cases are being diagnosed with increasing frequency, highlighting the need for a comprehensive understanding of its epidemiology (3).

Previous analyses using the Global Burden of Disease (GBD) database have provided valuable insights into the burden of thyroid cancer. The GBD study, which encompasses comprehensive epidemiological data from 204 countries and regions, has been instrumental in understanding the global trends of thyroid cancer incidence, prevalence, mortality, and disability-adjusted life years (DALYs) (4). For example, earlier GBD - based research has shown the increasing incidence of thyroid cancer in many parts of the world over the past few decades (5, 6). However, the complexity of the disease burden, especially in relation to factors such as the Socio - Demographic Index (SDI), age, and gender, still requires further in - depth exploration (7).

This current study builds upon previous GBD-related research and offers several distinct advantages. Firstly, it provides the most up-to-date assessment of thyroid cancer burden from 1990 to 2021, using the latest GBD 2021 results (8). This allows for a more accurate and timely understanding of the disease’s trends. Secondly, by stratifying the analysis by gender, 20 age groups, 21 GBD regions, 204 countries/territories, and 5 SDI categories, it offers a more comprehensive and detailed analysis of the factors associated with thyroid cancer burden (9). Thirdly, the study uses advanced statistical methods, such as the estimated annual percentage change (EAPC) and the Bayesian Age-Period-Cohort (BAPC) model (10, 11), to not only analyze the current situation but also predict future trends. This holistic approach can contribute to a more in - depth understanding of the epidemiology of thyroid cancer and provide a solid foundation for the development of targeted prevention and treatment strategies (12).

## Methods

### Data sources

The data in this study is derived from the GBD 2021 results (available at https://vizhub.healthdata.org/gbd-results/). The GBD 2021 study utilizes the latest epidemiological data and refined standardized methods to analyze age- and sex-specific mortality rates for 288 causes across 204 countries and regions, the prevalence and DALYs for 371 diseases and injuries, and the comparative risks for 88 risk factors (13). Detailed information on the study design and methodology of the GBD research has been extensively described in the existing GBD literature (4). Additionally, the Institutional Review Board at the University of Washington waived the informed consent requirement for accessing GBD data (14). All GBD 2021 analyses adhere to the guidelines for accurate and transparent health estimation reporting.

### Estimation framework

The GBD study employs complex modeling techniques to estimate the burden of thyroid cancer. The DisMod-MR 2.1 (Disease Model-Bayesian Meta-Regression) tool was used to calculate incidence and prevalence. This Bayesian geospatial software integrates various disease parameters, epidemiological relationships, and geospatial data to generate reliable estimates. For mortality estimation, the Cause of Death Ensemble Modeling (CODEm) framework was applied. This method incorporates both vital registration and verbal autopsy data, including data with nonspecific codes. The data were rigorously adjusted to ensure accuracy prior to analysis. CODEm combines multiple models to estimate mortality rates with higher precision. These models were applied to the 2021 database to provide a comprehensive estimate of thyroid cancer burden. The method takes into account differences in the study design and methodology of multiple data sources, ensuring consistent and accurate estimates of thyroid cancer incidence, prevalence, and mortality. To calculate the DALYs attributable to thyroid cancer, we summarized two components: years lived with disability (YLD), which quantifies the burden of living with stroke-related impairments, and years of life lost (YLL), which measures the impact of premature death.

### Sociodemographic index

SDI (Socio-Demographic Index) quantifies the level of development of a country or region using data on fertility rates, education levels, and per capita income. Ranging from 0 to 1, higher SDI values indicate faster socioeconomic development. SDI is known to be associated with disease incidence and mortality (4, 15). In this study, we categorized countries and regions into five SDI groups (low, low-middle, middle, middle-high, and high) to examine the relationship between the burden of thyroid cancer and socioeconomic development.

### Statistical analyses

To assess the trends in age-standardized rates (ASR) of thyroid cancer incidence, mortality, DALY, and prevalence, the study employed the estimated annual percentage change (EAPC).

The ASR was computed per 100,000 individuals utilizing the subsequent formula:


$$\mathrm{ASR}=\frac{\sum_{i=1}^{A}a_{i}w_{i}}{\sum_{i=1}^{A}w_{i}}\times 100,000$$

(*α* i: the age-specific rate in *i*th the age group; w: the number of people in the corresponding *i*th age group among the standard population; *A*: the number of age groups).

Prior to applying linear regression, we systematically evaluated the appropriateness of modeling the natural logarithm of ASR (ln[ASR]) using both linear and nonlinear regression approaches. We calculated the estimated annual percentage change (EAPC) in ASR to evaluate the average changing trends over a specified time interval. To validate the linearity assumption, we performed the following analyses: 1. Visual inspection of scatter plots and residual plots to assess for non-linear patterns (e.g., curvature, heteroscedasticity). 2. Comparison of model fit using R2 values and Akaike Information Criterion (AIC) between the linear model (Y = *α* + *β*X + *ε*, where Y = ln[ASR] and X = calendar year) and a quadratic model (Y = α + β₁X + β₂X2 + ε) to capture potential non-linear trends. 3. Testing for autocorrelation in residuals using the Durbin-Watson test to ensure independence of observations.

Results of these analyses supported the linear model as the most appropriate: 1. The linear model exhibited higher R2 values compared to quadratic models (ΔR2 < 0.03 for all outcomes). 2. AIC values were lower for the linear model, indicating better goodness-of-fit. 3. Residual plots showed no systematic patterns, and the Durbin-Watson statistic (d ≈ 2) confirmed no significant autocorrelation.

Therefore, we assumed the natural logarithm of ASR fit the linear regressions model Y = *α* + βX + e, where Y refers to ln (ASR), and X is the calendar year:


ln(ASR)=α+βX+e

EAPC=100×(e^β−1)

We identified an ASR as indicative of an increasing or decreasing trend over time if both the EAPC and its 95% Confidence interval (CI) were above or below zero, respectively. In instances where the 95% CI encompassed zero, we deemed the change in ASR statistically insignificant.

Spearman correlation was used to assess the relationship between SDI and the age-standardized incidence rate of thyroid cancer. Bayesian age-period-cohort (BAPC) models, implemented with integrated nested Laplace approximation (INLA), were utilized to predict the future prevalence, incidence, mortality, and DALYs associated with thyroid cancer through 2040. Previous research has demonstrated that BAPC offers superior coverage and precision compared to alternative prediction methods (16–18). Our analysis was conducted using the ‘INLA’ and ‘BAPC’ R packages, in accordance with established protocols. Statistical analyses were performed using R software (version 4.4.2), with a significance threshold set at *p* < 0.05.

The detailed foundation and mathematical formulation of the BAPC model are provided in Supplementary Methods S1. Sensitivity analyses for the EAPC estimation, including overdispersion tests and linearity assessments, are described in Supplementary Methods S2.

## Results

### Global level

**Figure 1:**
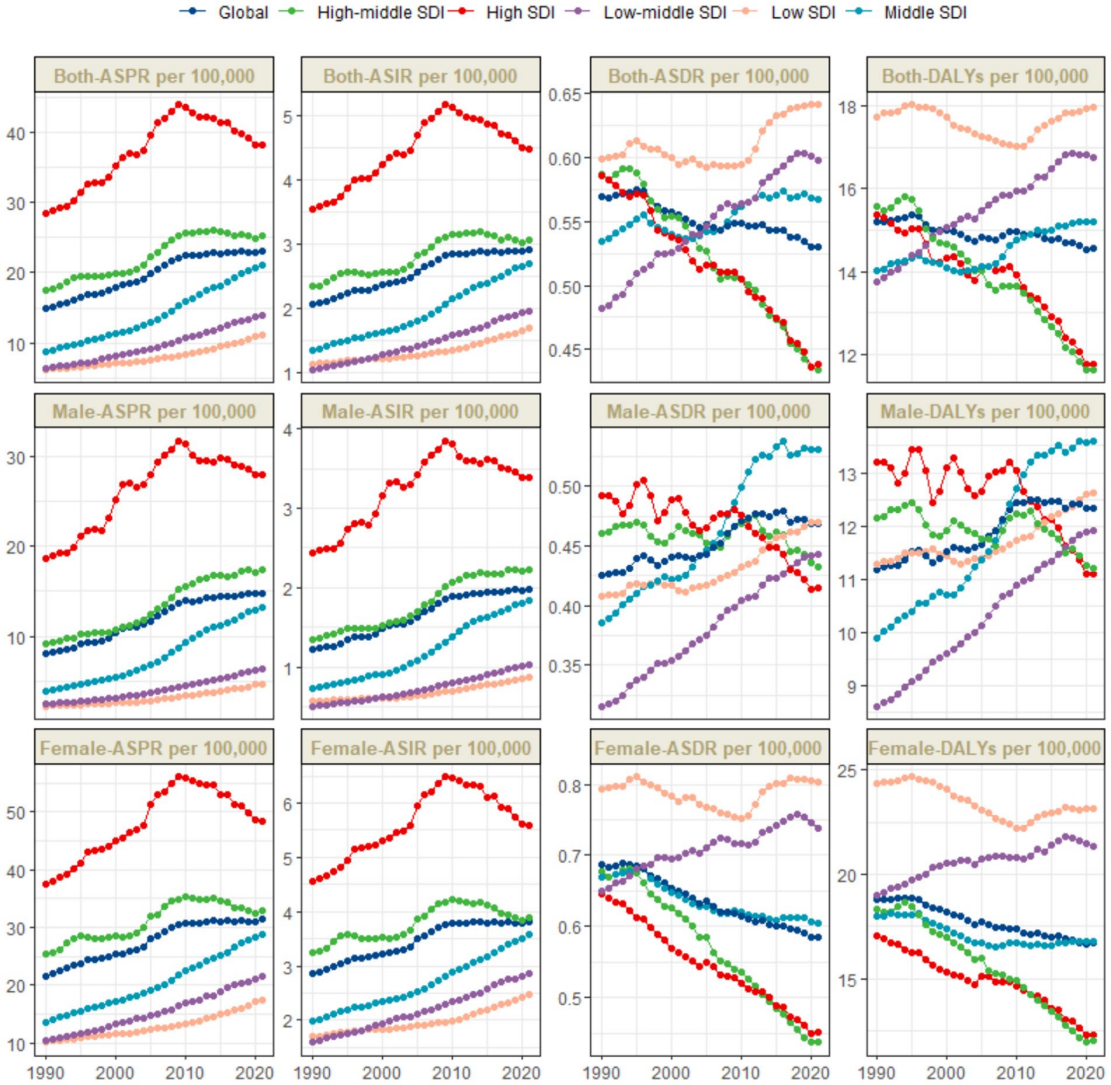
Trends in thyroid cancer prevalence, incidence, mortality and DALYs from 1990 to 2021.

In 2021, the global burden of thyroid cancer remained substantial. There were a total of 1,987,148.5 cases (95% Uncertainty Interval [UI]: 1,776,275.3 - 2,198,245.2), representing an increase of 193.68% compared to 1990. Not only did the absolute number of cases increase significantly, but the age - standardized prevalence rate (ASPR) also rose from 14.9 cases per 100,000 population (95% UI: 14.1–16) in 1990 to 23.1 cases per 100,000 population (95% UI: 20.7–25.6) in 2021. The estimated annual percentage change (EAPC) of ASPR was 1.58 (95% CI: 1.44–1.73) (Table 1 and Figure 1). In 2021, the global incidence of thyroid cancer reached 249,538 cases (95% UI: 223,290.3–274,638.2), representing a 177.62% increase compared to 1990. The ASIR of thyroid cancer increased from 2 cases per 100,000 population (95% UI: 2–2.2) in 1990 to 2.9 cases per 100,000 population (95% UI: 2.6–3.2) in 2021. During the study period, The EAPC of ASIR was 1.25 (95% CI: 1.14–1.37), indicating a continuous upward trend in the incidence of thyroid cancer.(Table 1 and Figure 1). The mortality caused by thyroid cancer was estimated to be 44,798.5 (95% UI: 39,924.7 – 48,541). The ASDR was 0.5 per 100,000 population (95% UI: 0.5–0.6), and the EAPC was −0.24 (95% CI: −0.27 to −0.21, Table 1 and Figure 1). In 2021, the global disability - adjusted life years (DALYs) for thyroid cancer were 1,246,484.8 (95% UI: 1,094,415.6 - 1,375,852.5). The age - standardized DALY rate was 14.6 per 100,000 population (95% UI: 12.8–16.1), and the EAPC was - 0.14 (95% CI: –0.17 to – 0.11) (Table 1 and Figure 1).

**Table 1:** The prevalence, incidence, mortality and DALYs of thyroid cancer in 1990 and 2021, and changes from 1990 to 2021 at the global level and different regions.

Location	1990		2021		EAPC_95%CI
	Number	ASR	Number	ASR	
Prevalence					
Global	676648.8 (636788.9–727722.8)	14.9 (14.1–16)	1987148.5 (1776275.3–2198245.2)	23.1 (20.7–25.6)	1.58 (1.44 to 1.73)
Andean Latin America	2640.4 (2211.1–3138.8)	9.8 (8.1–11.5)	18047.3 (13837.2–22,793)	28.1 (21.6–35.6)	3.55 (3.34 to 3.77)
Australasia	4599.9 (4076.9–5210.9)	20.6 (18.3–23.3)	16211.2 (13090.8–19620.8)	38.9 (31.4–47.6)	2.87 (2.29 to 3.47)
Caribbean	3118.1 (2873.6–3378.8)	10.6 (9.8–11.5)	9357.3 (8102.5–10834.1)	17.8 (15.4–20.6)	1.86 (1.69 to 2.04)
Central Asia	6945.8 (6347.8–7593.4)	12.7 (11.6–13.9)	13021.3 (11400.3–14782.4)	13.4 (11.7–15.2)	0.16 (−0.61 to 0.93)
Central Europe	34174.4 (32484.3–36024.9)	23.6 (22.4–24.8)	37892.2 (34073.8–41467.7)	21.9 (19.7–24)	−0.33 (−0.52 to −0.14)
Central Latin America	11472.2 (11049.9–11916.4)	10.2 (9.8–10.6)	58,071 (51803–65647.6)	21.8 (19.4–24.6)	2.39 (2.28 to 2.5)
Central Sub-Saharan Africa	870.5 (619–1301.5)	2.6 (1.8–3.9)	3,432 (2194.6–5420.7)	4 (2.5–6.3)	1.42 (1.14 to 1.7)
East Asia	95529.4 (76898.9–113229.6)	8.5 (6.9–10.1)	411402.4 (334530–513557.1)	20.5 (16.8–25.6)	3.16 (3 to 3.33)
Eastern Europe	50163.3 (47847.6–52997.6)	18.9 (18.1–20)	75906.4 (68504.6–84343.5)	25.9 (23.3–28.8)	1.47 (0.97 to 1.97)
Eastern Sub-Saharan Africa	12,047 (9301.5–15328.6)	9.4 (7.3–12)	48,137 (34449.7–73226.7)	15.4 (11.2–23.1)	1.47 (1.29 to 1.66)
High SDI	288686.4 (280129.1–297,109)	28.5 (27.6–29.3)	598,370 (566881.9–629719.5)	38.2 (36.3–40.4)	1.23 (0.93 to 1.53)
High-income Asia Pacific	55344.6 (51670.9–60632.8)	26.9 (25.1–29.6)	110959.1 (98931.9–129334.7)	37.1 (33.2–43.8)	1.42 (0.91 to 1.93)
High-income North America	100673.1 (97445.8–103,313)	32.1 (31.1–32.9)	237,732 (226609–247666.5)	45.5 (43.6–47.3)	1.23 (1.05 to 1.42)
High-middle SDI	186,568 (174097.6–198522.8)	17.6 (16.4–18.7)	448569.8 (401405.4–510752.5)	25.2 (22.6–28.8)	1.4 (1.22 to 1.58)
Low SDI	21971.9 (17309–27986.9)	6.2 (4.9–7.9)	92787.3 (71789.9–126,590)	11.1 (8.7–14.9)	1.87 (1.76 to 1.99)
Low-middle SDI	56029.2 (47678.1–71121.3)	6.3 (5.3–7.9)	251682.8 (206843.3–308118.1)	14 (11.5–16.9)	2.67 (2.64 to 2.7)
Middle SDI	122505.4 (108257.6–142672.6)	8.6 (7.7–10.1)	594136.2 (493696.7–673,492)	21 (17.5–23.8)	3.03 (2.95 to 3.12)
North Africa and Middle East	31,166 (25920.4–41,990)	13 (10.8–17.4)	183491.2 (151632.4–216130.2)	30.7 (25.4–36)	3.16 (2.99 to 3.33)
Oceania	308.3 (199–418.5)	6.8 (4.5–9.3)	960.2 (576–1382.1)	8.6 (5.2–12.3)	0.59 (0.48 to 0.7)
South Asia	53580.2 (43818.6–70783.2)	5.9 (4.9–7.8)	282509.1 (227052.9–343508.3)	15.3 (12.3–18.5)	3.24 (3.16 to 3.33)
Southeast Asia	45,506 (36198.2–51840.3)	12.7 (10.3–14.5)	206164.7 (161849.8–244210.6)	26.9 (21.1–31.8)	2.33 (2.23 to 2.43)
Southern Latin America	6,690 (5949.1–7523.9)	14.1 (12.6–15.9)	15500.3 (13535.1–17754.5)	19.7 (17.1–22.6)	1.21 (1 to 1.42)
Southern Sub-Saharan Africa	2655.2 (2243.8–3,153)	6.9 (5.8–8.2)	8000.6 (6622.7–9541.6)	10.7 (8.9–12.7)	1.8 (1.54 to 2.05)
Tropical Latin America	9237.1 (8742.4–9743.8)	7.8 (7.4–8.2)	33082.7 (31066.3–34983.6)	12.6 (11.8–13.3)	1.32 (1.13 to 1.51)
Western Europe	148253.3 (140945–155876.6)	31.1 (29.5–32.7)	211123.9 (193366–229245.3)	32.7 (29.9–35.5)	0.51 (0.09 to 0.94)
Western Sub-Saharan Africa	1,674 (1212.8–2,106)	1.2 (0.9–1.5)	6146.3 (4496.6–8323.4)	1.8 (1.3–2.3)	1.09 (1 to 1.18)
Incidence					
Global	89885.5 (84681.3–96998.8)	2.1 (2–2.2)	249,538 (223290.3–274638.2)	2.9 (2.6–3.2)	1.25 (1.14 to 1.37)
Andean Latin America	422.2 (354–495.4)	1.8 (1.5–2.1)	2,424 (1907.2–3044.3)	3.9 (3.1–4.8)	2.6 (2.44 to 2.77)
Australasia	585.9 (522.4–660.1)	2.6 (2.3–2.9)	1949.1 (1569.7–2343.2)	4.6 (3.7–5.5)	2.61 (2.06 to 3.16)
Caribbean	442.8 (410.8–479.3)	1.6 (1.5–1.7)	1244.9 (1085.7–1427.4)	2.4 (2.1–2.7)	1.5 (1.32 to 1.69)
Central Asia	914 (840.8–996.1)	1.7 (1.6–1.9)	1630.9 (1432.2–1845.3)	1.7 (1.5–2)	0.01 (−0.73 to 0.75)
Central Europe	4654.9 (4428.6–4883.6)	3.2 (3–3.4)	4876.2 (4406.6–5323.5)	2.7 (2.4–2.9)	−0.65 (−0.86 to −0.45)
Central Latin America	1712.2 (1651.2–1775.4)	1.7 (1.7–1.8)	7752.6 (6907.4–8701.4)	3 (2.6–3.3)	1.66 (1.52 to 1.8)
Central Sub-Saharan Africa	153.7 (111.5–225.9)	0.6 (0.4–0.8)	495.7 (319.2–770.7)	0.7 (0.4–1.1)	0.63 (0.42 to 0.83)
East Asia	13203.4 (10809.5–15460.8)	1.3 (1.1–1.5)	50885.2 (41562–63161.9)	2.5 (2.1–3.1)	2.43 (2.26 to 2.6)
Eastern Europe	6467.8 (6164.2–6,831)	2.4 (2.3–2.6)	9,617 (8698.1–10650.3)	3.2 (2.9–3.5)	1.27 (0.8 to 1.75)
Eastern Sub-Saharan Africa	1908.4 (1516.6–2387.8)	1.8 (1.5–2.2)	6384.4 (4629.9–9478.7)	2.4 (1.8–3.5)	0.76 (0.6 to 0.92)
High SDI	36533.2 (35292–37708.3)	3.5 (3.4–3.7)	72995.8 (68514–76746.9)	4.5 (4.3–4.7)	1.01 (0.75 to 1.28)
High-income Asia Pacific	6950.1 (6496.3–7654.3)	3.4 (3.2–3.8)	14277.6 (12630.3–16476.5)	4.4 (3.9–5.2)	1.15 (0.68 to 1.61)
High-income North America	12130.3 (11695.2–12,450)	3.8 (3.7–3.9)	28289.1 (26782.7–29,536)	5.3 (5.1–5.5)	1.15 (0.97 to 1.33)
High-middle SDI	24410.6 (22753.3–25919.8)	2.3 (2.2–2.5)	55158.1 (49518.3–62,489)	3.1 (2.8–3.5)	1.05 (0.89 to 1.21)
Low SDI	3431.3 (2759.4–4295.6)	1.1 (0.9–1.4)	12358.4 (9598.7–16514.5)	1.7 (1.3–2.2)	1.23 (1.12 to 1.34)
Low-middle SDI	8233.9 (7035–10302.4)	1 (0.9–1.3)	33,464 (27896.3–40292.7)	2 (1.7–2.3)	2.09 (2.07 to 2.12)
Middle SDI	17155.2 (15282.6–19997.3)	1.4 (1.2–1.6)	75356.9 (62756–84674.8)	2.7 (2.3–3)	2.37 (2.28 to 2.46)
North Africa and Middle East	3,792 (3150.6–5143.1)	1.7 (1.4–2.3)	21222.4 (17602–24974.6)	3.7 (3.1–4.3)	2.89 (2.72 to 3.06)
Oceania	44.6 (29.9–60.2)	1.2 (0.8–1.6)	131.1 (80.2–185.4)	1.4 (0.9–1.9)	0.33 (0.24 to 0.42)
South Asia	7853.1 (6476.2–10284.5)	1 (0.8–1.3)	37335.6 (30262.7–44931.4)	2.1 (1.7–2.6)	2.54 (2.46 to 2.62)
Southeast Asia	6440.6 (5205.8–7276.5)	2 (1.7–2.3)	26,559 (20898.8–31183.5)	3.6 (2.9–4.2)	1.82 (1.74 to 1.9)
Southern Latin America	1000.1 (896.4–1118.9)	2.1 (1.9–2.4)	2046.9 (1787.2–2,334)	2.5 (2.2–2.9)	0.7 (0.48 to 0.92)
Southern Sub-Saharan Africa	376.9 (317.8–447.9)	1.1 (0.9–1.3)	1116.8 (934.8–1315.9)	1.6 (1.3–1.9)	1.54 (1.3 to 1.79)
Tropical Latin America	1371.6 (1299.4–1443.4)	1.3 (1.2–1.3)	4491.1 (4197.7–4757.9)	1.7 (1.6–1.8)	0.76 (0.6 to 0.91)
Western Europe	19206.5 (18291.7–20159.2)	3.9 (3.7–4.1)	26004.5 (23787.6–28201.5)	3.8 (3.5–4.2)	0.28 (−0.1 to 0.67)
Western Sub-Saharan Africa	254.3 (186.2–312.4)	0.2 (0.2–0.3)	803.8 (597.7–1068.9)	0.3 (0.2–0.3)	0.52 (0.45 to 0.58)
Deaths					
Global	21,893 (20437.5–24108.1)	0.6 (0.5–0.6)	44798.5 (39924.7–48,541)	0.5 (0.5–0.6)	−0.24 (−0.27 to −0.21)
Andean Latin America	182.3 (153.4–211.5)	0.9 (0.8–1)	641.2 (507.9–794.5)	1.1 (0.9–1.4)	0.66 (0.54 to 0.77)
Australasia	97.6 (87.2–109.1)	0.4 (0.4–0.5)	199.1 (161.3–237.4)	0.4 (0.3–0.4)	0.14 (−0.13 to 0.41)
Caribbean	142.5 (131.7–156)	0.6 (0.5–0.6)	321.3 (280.7–367.5)	0.6 (0.5–0.7)	0.42 (0.2 to 0.65)
Central Asia	253.3 (235.5–274.3)	0.5 (0.5–0.6)	339.3 (301.7–378.5)	0.4 (0.4–0.5)	−0.81 (−1.43 to −0.18)
Central Europe	1266.4 (1213.2–1316.6)	0.9 (0.8–0.9)	985.7 (898–1064.6)	0.4 (0.4–0.5)	−2.37 (−2.65 to −2.1)
Central Latin America	635.6 (613.9–657.6)	0.8 (0.8–0.8)	1964.6 (1748.8–2165.4)	0.8 (0.7–0.9)	−0.07 (−0.26 to 0.11)
Central Sub-Saharan Africa	78.4 (57.7–112.8)	0.4 (0.3–0.5)	182.8 (118.4–284)	0.3 (0.2–0.6)	−0.11 (−0.23 to 0.01)
East Asia	3780.9 (3211.5–4380.1)	0.5 (0.4–0.6)	8063.8 (6456.3–9800.1)	0.4 (0.3–0.5)	−0.65 (−0.74 to −0.55)
Eastern Europe	1349.5 (1274–1423.5)	0.5 (0.5–0.5)	1650.5 (1508.2–1807.4)	0.5 (0.4–0.5)	−0.25 (−0.6 to 0.09)
Eastern Sub-Saharan Africa	838.7 (685.7–1015.1)	1 (0.8–1.2)	1800.9 (1337.6–2485.5)	1 (0.7–1.3)	−0.18 (−0.29 to −0.07)
High SDI	6504.9 (6098.3–6798.4)	0.6 (0.5–0.6)	9730.2 (8465.5–10437.2)	0.4 (0.4–0.5)	−0.91 (−0.96 to −0.86)
High-income Asia Pacific	1235.9 (1126.1–1404.5)	0.6 (0.6–0.7)	2843.9 (2308.9–3185.8)	0.5 (0.4–0.6)	−0.82 (−0.98 to −0.65)
High-income North America	1391.8 (1285–1450.1)	0.4 (0.4–0.4)	2765.7 (2473–2948.9)	0.4 (0.4–0.4)	0.15 (0.06 to 0.23)
High-middle SDI	5609.4 (5200.9–5934.9)	0.6 (0.5–0.6)	8436.7 (7553.5–9304.5)	0.4 (0.4–0.5)	−1.03 (−1.09 to −0.97)
Low SDI	1454.5 (1199.2–1778.3)	0.6 (0.5–0.7)	3392.4 (2687.9–4328.7)	0.6 (0.5–0.8)	0.19 (0.11 to 0.28)
Low-middle SDI	3015.5 (2624–3757.1)	0.5 (0.4–0.6)	8531.2 (7410.5–9771.6)	0.6 (0.5–0.7)	0.74 (0.71 to 0.78)
Middle SDI	5275.6 (4815.1–6144.8)	0.5 (0.5–0.6)	14666.5 (12466.8–16170.9)	0.6 (0.5–0.6)	0.21 (0.16 to 0.26)
North Africa and Middle East	657.9 (546.1–936.5)	0.4 (0.3–0.6)	1935.2 (1676.1–2236.4)	0.4 (0.4–0.5)	0.64 (0.49 to 0.79)
Oceania	14.6 (10.3–19.3)	0.6 (0.4–0.7)	36.6 (23.3–50.4)	0.5 (0.4–0.7)	−0.13 (−0.16 to −0.1)
South Asia	2914.5 (2454.1–3700.4)	0.5 (0.4–0.6)	9323.8 (7794.2–10741.5)	0.6 (0.5–0.7)	0.92 (0.88 to 0.97)
Southeast Asia	2008.1 (1698.1–2303.1)	0.8 (0.7–1)	5642.9 (4564.6–6450.7)	0.9 (0.7–1)	0.32 (0.24 to 0.4)
Southern Latin America	359 (324.7–394.6)	0.8 (0.7–0.9)	485.2 (425.8–553.7)	0.6 (0.5–0.6)	−1.01 (−1.26 to −0.77)
Southern Sub-Saharan Africa	123.6 (102.4–148.3)	0.5 (0.4–0.6)	323.8 (264.5–372.4)	0.6 (0.5–0.7)	0.92 (0.65 to 1.18)
Tropical Latin America	509 (480–537.2)	0.6 (0.5–0.6)	1252.8 (1142–1,332)	0.5 (0.5–0.5)	−0.6 (−0.69 to −0.5)
Western Europe	3,951 (3667.4–4173.8)	0.7 (0.6–0.7)	3829.2 (3318.9–4190.8)	0.4 (0.3–0.4)	−1.7 (−1.77 to −1.64)
Western Sub-Saharan Africa	102.7 (77.8–122.8)	0.1 (0.1–0.1)	210.4 (165.3–263.6)	0.1 (0.1–0.1)	−0.51 (−0.59 to −0.43)
Disability-adjusted life years					
Global	646740.5 (599118.8–717,357)	15.2 (14.2–16.8)	1246484.8 (1094415.6–1375852.5)	14.6 (12.8–16.1)	−0.14 (−0.17 to −0.11)
Andean Latin America	5273.8 (4419.1–6186.5)	23 (19.3–27)	16689.8 (13207.7–20766.9)	27.5 (21.7–34.3)	0.54 (0.43 to 0.65)
Australasia	2,544 (2273.1–2867.8)	11 (9.9–12.5)	5041.1 (4103.8–6033.3)	10.6 (8.7–12.8)	0.51 (0.21 to 0.82)
Caribbean	4116.6 (3769–4556.3)	14.9 (13.7–16.5)	8690.2 (7532.9–10099.5)	16.3 (14.1–19)	0.46 (0.25 to 0.68)
Central Asia	8144.5 (7568–8817.1)	15.9 (14.7–17.2)	10272.9 (9055.5–11543.5)	11.7 (10.3–13)	−1.19 (−1.81 to −0.57)
Central Europe	34786.3 (33224.2–36474.4)	23.5 (22.4–24.6)	23433.7 (21250.4–25475.1)	11.6 (10.5–12.7)	−2.47 (−2.76 to −2.17)
Central Latin America	18293.1 (17666.1–18920.9)	19.5 (18.9–20.2)	52186.9 (47013.3–58083.4)	20.4 (18.4–22.7)	0 (−0.2 to 0.2)
Central Sub-Saharan Africa	2545.3 (1889.3–3609.5)	9.4 (6.9–13.7)	5956.3 (3862.9–9187.8)	8.9 (5.8–13.9)	−0.19 (−0.31 to −0.06)
East Asia	116582.2 (98062.3–136720.9)	12.2 (10.4–14.2)	213609.2 (173066.2–262361.7)	10.3 (8.3–12.5)	−0.56 (−0.67 to −0.46)
Eastern Europe	37,930 (35923.3–40329.4)	13.9 (13.1–14.7)	42085.6 (38218.3–46388.2)	12.9 (11.7–14.1)	−0.29 (−0.68 to 0.11)
Eastern Sub-Saharan Africa	31,046 (25044.5–38047.3)	30.1 (24.5–36.7)	66490.9 (48520.2–94804.4)	27.8 (20.6–38.4)	−0.43 (−0.55 to −0.32)
High SDI	164381.5 (155870.6–173717.9)	15.4 (14.6–16.3)	220135.9 (201450.2–237834.8)	11.8 (10.8–12.8)	−0.77 (−0.86 to −0.68)
High-income Asia Pacific	30855.9 (28505.4–35131.3)	15.4 (14.1–17.5)	50783.2 (43704.3–57571.4)	11.8 (10.5–13.7)	−0.75 (−0.99 to −0.5)
High-income North America	37,316 (34968.1–39721.4)	11.2 (10.5–12)	70641.6 (65003.7–76456.6)	12 (11–13)	0.21 (0.11 to 0.31)
High-middle SDI	158942.1 (146716.9–170152.9)	15.6 (14.4–16.6)	218961.3 (196634.7–244418.1)	11.6 (10.4–13)	−0.99 (−1.05 to −0.92)
Low SDI	52661.5 (43472.1–63947.7)	17.8 (14.6–21.6)	120143.3 (93672.8–157131.4)	18 (14.2–23.1)	−0.05 (−0.12 to 0.02)
Low-middle SDI	103647.8 (90342.8–129887.4)	13.8 (12–17.1)	269914.2 (229089.1–316826.3)	16.8 (14.3–19.4)	0.67 (0.63 to 0.7)
Middle SDI	166,177 (149966.3–190691.4)	14 (12.8–16.2)	416217.6 (350751–460888.7)	15.2 (12.8–16.8)	0.28 (0.22 to 0.34)
North Africa and Middle East	21944.4 (18189.4–30745.7)	11 (9.1–15.5)	65316.2 (55454.2–76496.3)	12.7 (10.9–14.8)	0.76 (0.62 to 0.9)
Oceania	480.3 (324.9–641.5)	13.9 (9.8–18.5)	1173.6 (724.9–1641.6)	13.5 (8.6–18.6)	−0.1 (−0.14 to −0.06)
South Asia	105302.9 (88400.4–135793.4)	14.2 (12–18.2)	302256.8 (249827.8–356789.4)	18.2 (15.1–21.4)	0.85 (0.81 to 0.88)
Southeast Asia	62694.8 (51465.3–70374.5)	21.4 (17.9–24.3)	164547.4 (130332.7–189174.4)	23.6 (18.8–27)	0.27 (0.21 to 0.34)
Southern Latin America	9491.8 (8618.3–10469.6)	20.3 (18.4–22.3)	11959.8 (10465.3–13594.3)	14.3 (12.5–16.2)	−1.02 (−1.28 to −0.75)
Southern Sub-Saharan Africa	4035.3 (3398.8–4776.4)	12.4 (10.3–14.8)	10253.2 (8481–11,926)	15.7 (12.8–18.1)	1 (0.72 to 1.27)
Tropical Latin America	15021.1 (14259–15822.7)	14.8 (14–15.6)	32680.5 (30547–34741.4)	12.7 (11.8–13.5)	−0.65 (−0.75 to −0.55)
Western Europe	94707.5 (89027.2–100543.1)	17.6 (16.6–18.8)	84464.5 (75823.1–92550.5)	10.5 (9.4–11.5)	−1.5 (−1.64 to −1.36)
Western Sub-Saharan Africa	3628.7 (2761.6–4379.4)	3.2 (2.4–3.8)	7951.2 (6109.3–10,289)	2.8 (2.2–3.5)	−0.49 (−0.56 to −0.42)

### Regional level

The global burden of thyroid cancer exhibits remarkable regional disparities, which are closely associated with the Socio–demographic Index (SDI) levels. The ASPR shows significant differences. The incidence is highest in high - SDI regions, reaching 38.2 per 100,000 population (95% UI: 36.3–40.4), while the lowest incidence is reported in low–SDI regions, at 11.1 per 100,000 population (95% UI: 8.7–14.9) (Table 1 and Figure 1).

The temporal trends of the ASPR and ASIR reveal distinct patterns across different SDI levels. Specifically, in high–SDI regions, there are two distinct phases. From 1990 to 2009, there was a marked upward trend, followed by a significant downward trend. The overall EAPC was 1.23 (95% CI: 0.93–1.53). In these regions, the ASPR gradually increased from 28.5 per 100,000 population (95% UI: 27.6–29.3) in 1990 to 44 per 100,000 population (95% UI: 42–46.3) in 2009, and then decreased to 38.2 per 100,000 population (95% UI: 36.3–40.4) in 2021. The ASIR increased from 3.5 per 100,000 population (95% UI: 3.4–3.7) in 1990 to 5.2 per 100,000 population (95% UI: 4.9–5.4) in 2009, and then declined to 4.5 per 100,000 population (95% UI: 4.3–4.7) in 2021 (Table 1 and Figure 1). In contrast, in other SDI regions, there has been a long–term upward trend. The middle - SDI regions had the highest growth rate, with an EAPC of 3.03 (95% CI: 2.95–3.12) (Table 1 and Figure 1).

**Figure 2:**
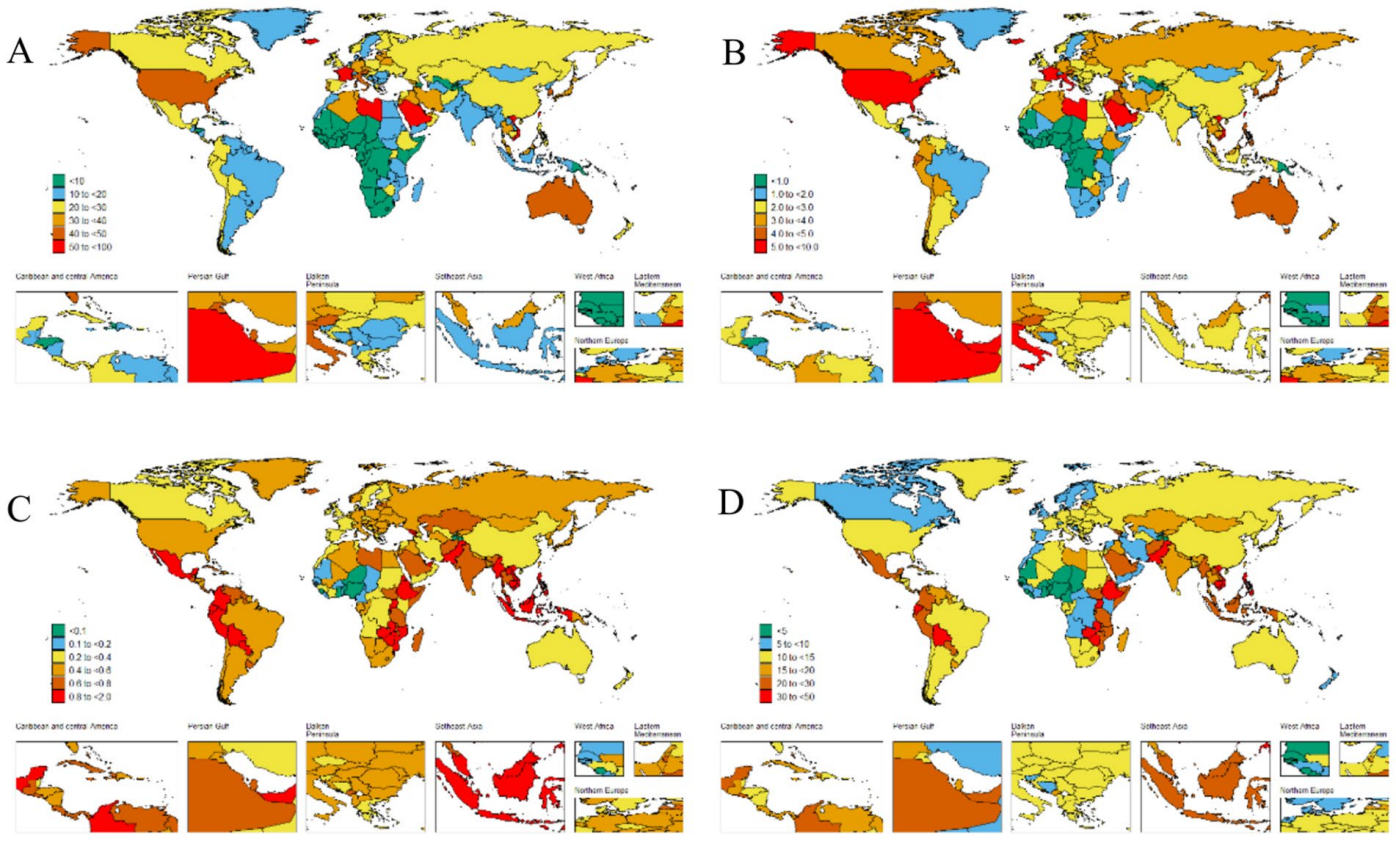
The global disease burden of thyroid cancer for both sexes in 204 countries and territories. **(A)** Prevalence rate. **(B)** Incidence rate. **(C)** Death rate. **(D)** DALYs rate.

In these regions, there is no significant difference in the ASDR. Among them, the low SDI, low - middle SDI, and middle SDI regions report relatively higher ASDRs, with the lowest SDI region having the highest. Meanwhile, the high SDI and upper–middle SDI regions have the lowest values for this indicator. Specifically, the ASDR in the low SDI region is 0.6 per 100,000 population (95% UI: 0.5–0.8), the ASDR in the low - middle SDI region is 0.6 per 100,000 population (95% UI: 0.5–0.7), and the ASDR in the middle SDI region is 0.6 per 100,000 population (95% UI: 0.5–0.6), while the ASIR in the high SDI and upper–middle SDI regions is 0.4 per 100,000 population (95% UI: 0.4–0.5) (Table 1 and Figures 1, 2C).

The disease burden quantified by the age - standardized DALY rate further emphasizes these regional differences. The burden is highest in the low SDI region, with a DALY rate of 18 per 100,000 population (95% UI: 14.2–23.1), while it is lowest in the upper - middle SDI region, at 11.6 per 100,000 population (95% UI: 10.4–13) (Table 1 and Figures 1, 2D).

Our research findings indicate that the High-income North America bear the highest burden of thyroid cancer prevalence globally. Specifically, the ASPR in the High-income North America is the highest, reaching 45.5 per 100,000 population (95% UI: 43.6–47.3), followed closely by Australasia, with 38.9 per 100,000 population (95% UI: 31.4–47.6) (Table 1 and Figure 2A). The High-income Asia Pacific also shows a relatively high ASPR, with 37.1 per 100,000 population (95% UI: 33.2–43.8), ranking third among the studied regions.

Our analysis shows that the incidence of thyroid cancer in South America and Europe warrants high attention. The research results indicate that the Andean Latin America and Western Europe are among the regions with relatively high ASIR globally. Specifically, the ASIR in the regions along the Andean Latin America is 3.9 per 100,000 population (95% UI: 3.1–4.8), and the ASIR in Western Europe is 3.8 per 100,000 population (95% UI: 3.5–4.2). In contrast, the ASIR in the Western Sub-Saharan Africa is the lowest, at 0.3 per 100,000 population (95% UI: 0.2–0.3) (Table 1 and Figure 2B).

In addition, we found that the ASDR of thyroid cancer in the Eastern Sub-Saharan Africa was significantly higher, at 1 per 100,000 population (95% UI: 0.7–1.3). From 1990 to 2021, the thyroid cancer ASDR increased the most in the Southern Sub-Saharan Africa (EAPC 0.92, 95% CI: 0.65–1.18) and decreased the most in Central Europe (EAPC –2.37, 95% CI: −2.65 to −2.1) (Table 1). Conversely, the region with the highest age - standardized DALYs rate was the Eastern Sub-Saharan Africa, at 27.8 per 100,000 population (95% UI: 20.6–38.4). From 1990 to 2021, the age - standardized DALYs incidence of thyroid cancer increased the most in Southern Sub-Saharan Africa (EAPC 1, 95% CI: 0.72 to 1.27) and decreased the most in Central Europe (EAPC -2.47, 95% CI: −2.76 to −2.17) (Table 1).

**Figure 3:**
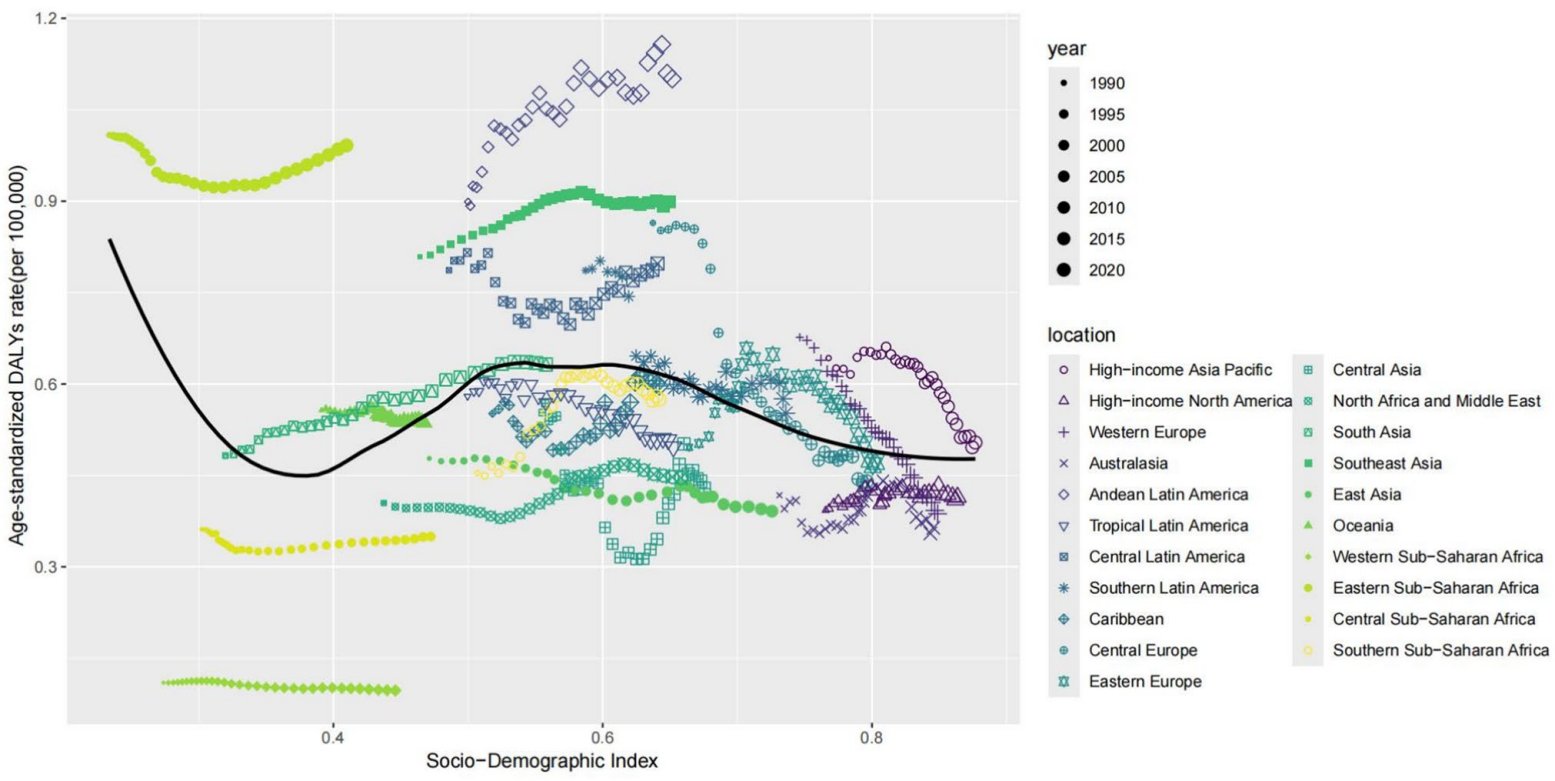
Association between age-standardized thyroid cancer DALYs rate and socio-demographic index in 21 regions.

At the regional level, we found an association between the SDI and the age–standardized DALYs rates of thyroid cancer from 1990 to 2021. The age–standardized DALYs rates decreased exponentially with the increase of the SDI, reaching a minimum of approximately 0.36, then increased to a maximum of 0.6, and then decreased again. From 1990 to 2021, the DALYs rates in the Eastern Sub-Saharan Africa, South Asia, Andean Latin America, and Central Latin America were higher than expected based on their SDI. In contrast, the burden in regions such as Central Sub - Saharan Africa, East Asia, North Africa and the Middle East, Tropical Latin America, High - income North America, Central Asia, and Central Latin America was lower than expected from 1990 to 2021 (Figure 3).

**Figure 4:**
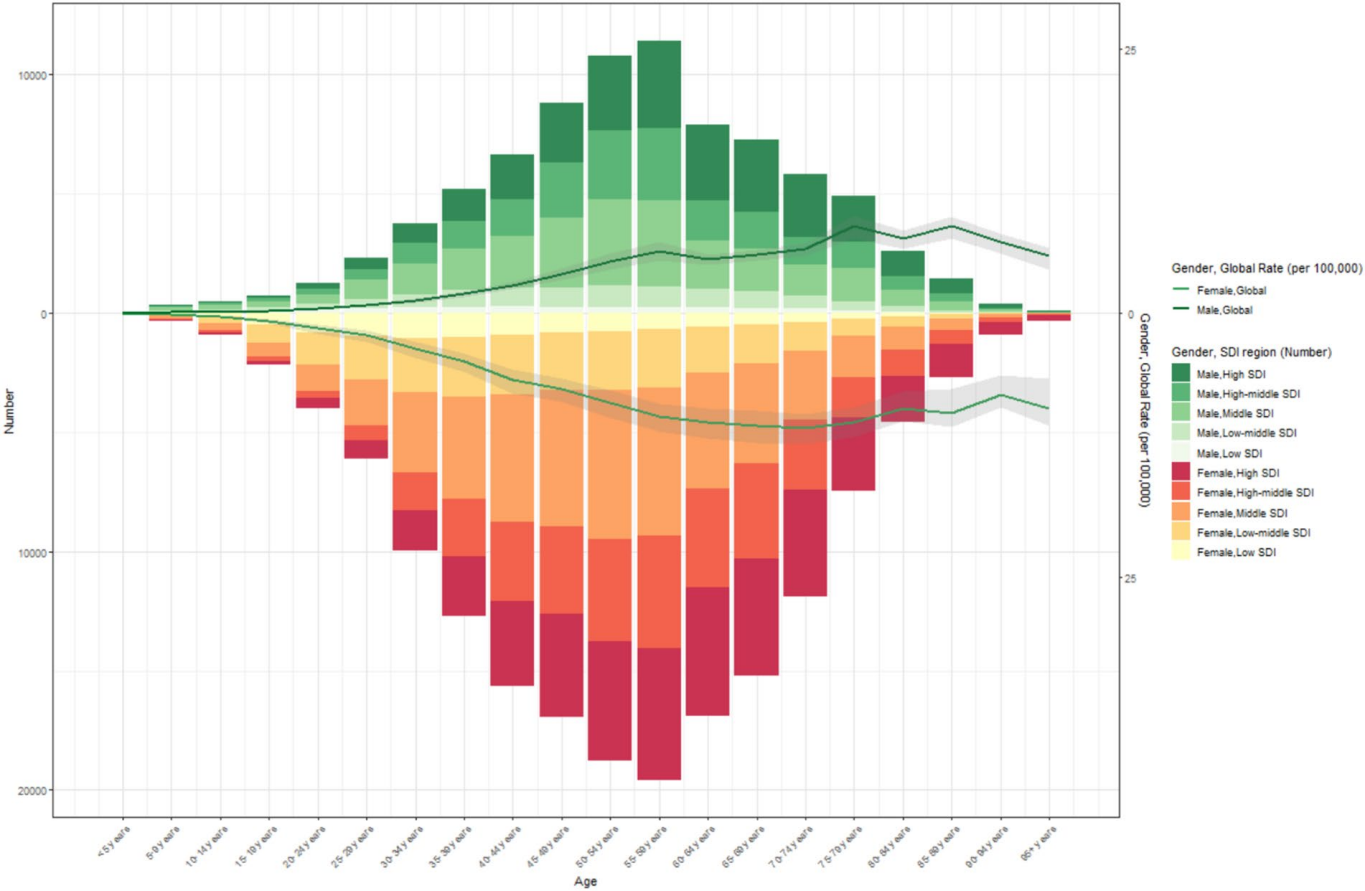
The age-specific numbers and ASIRs of thyroid cancer by SDI regions in 2021.

**Figure 5:**
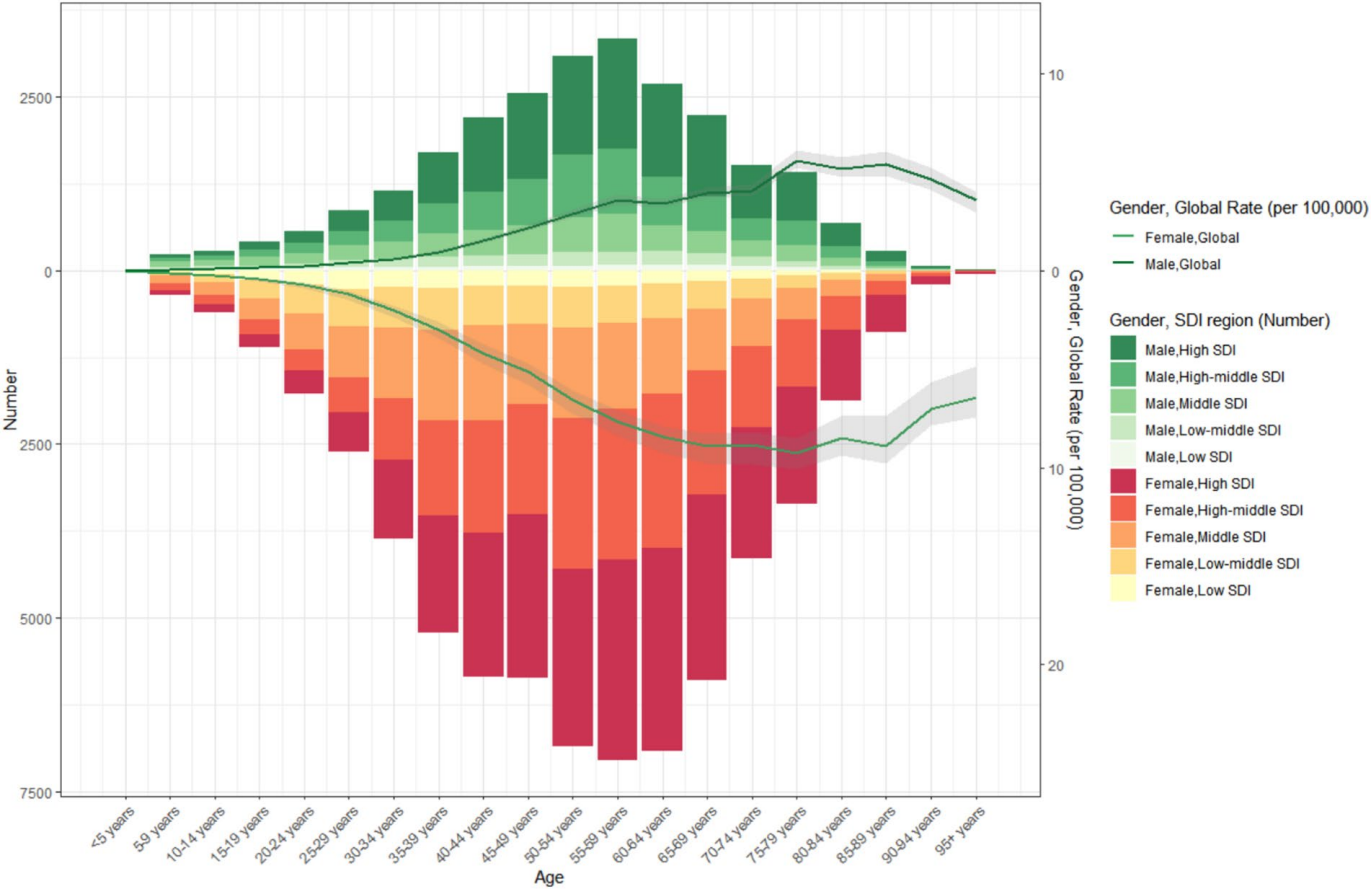
The age-specific numbers and ASIRs of thyroid cancer by SDI regions in 1990.

It is also noteworthy that in regions with a higher SDI, the proportion of thyroid cancer cases among the elderly is lower. In 1990 and 2021, the proportion of thyroid cancer cases among the young is higher in regions with a higher SDI. From 1990 to 2021, the incidence of thyroid cancer first increased gradually with age, particularly among the young population, and then decreased gradually. Across all SDI regions, the incidence of thyroid cancer in the young population is significantly higher than that in the elderly (Figures 4, 5).

### National level

The ASPR of thyroid cancer varies from approximately 0.1 to 60.1 per 100,000 population. Among all countries, Saudi Arabia (60.1 per 100,000 population; 95% UI: 44.7–79.4), France (58.8 per 100,000 population; 95% UI: 48.4–70.9), Iceland (55.2 per 100,000 population; 95% UI: 45–68.6), and Libya (51.1 per 100,000 population; 95% UI: 35.5–70.9) exhibited the highest ASPRs (Figure 2A and Supplementary Table S1). The most significant increase in the ASPR of thyroid cancer was observed in Cabo Verde (EAPC: 6.05, 95% CI 5.07–7.04) (Supplementary Table S1).

The country–specific distribution of the ASIR is presented in Figure 2B and Supplementary Table S2. Saudi Arabia had the highest ASIR of thyroid cancer (7.1 cases per 100,000 population; 95% UI: 5.4–9.3), while Tajikistan had the lowest (0 cases per 100,000 population; 95% UI: 0–0).

Analyzing the data on the ASDR and DALYs caused by thyroid cancer in 2021, we observed a significant consistency among the most severely affected countries. Ethiopia, Bolivia (Plurinational State of), and Zimbabwe stood out in both indicators. Ethiopia led in both categories, with the highest ASDR (1.6 per 100,000 population; 95% UI: 1.1–2.4) and the highest DALY s (44.2 per 100,000 population; 95% UI: 29.8–67.4). Zimbabwe ranked third in ASDR (1.4 per 100,000 population; 95% UI: 1–1.8) and second in DALYs (38.9 per 100,000 population; 95% UI: 27.4–50.7), while the Bolivia (Plurinational State of) ranked second in ASDR (1.6 per 100,000 population; 95% UI: 1–2.2) and third in DALYs (38.4 per 100,000 population; 95%: 25.3–53.3) (Figure 2D and Supplementary Tables S3, S4). Poland (EAPC –3.52, 95% CI –4.09 to −2.94), Kazakhstan (EAPC –2.69, 95% CI –3.27 to −2.1), and Cyprus (EAPC -2.63, 95% CI –2.78 to −2.48) had the largest decreases in the age–standardized DALYs numbers due to thyroid cancer (Supplementary Table S4).

**Figure 6:**
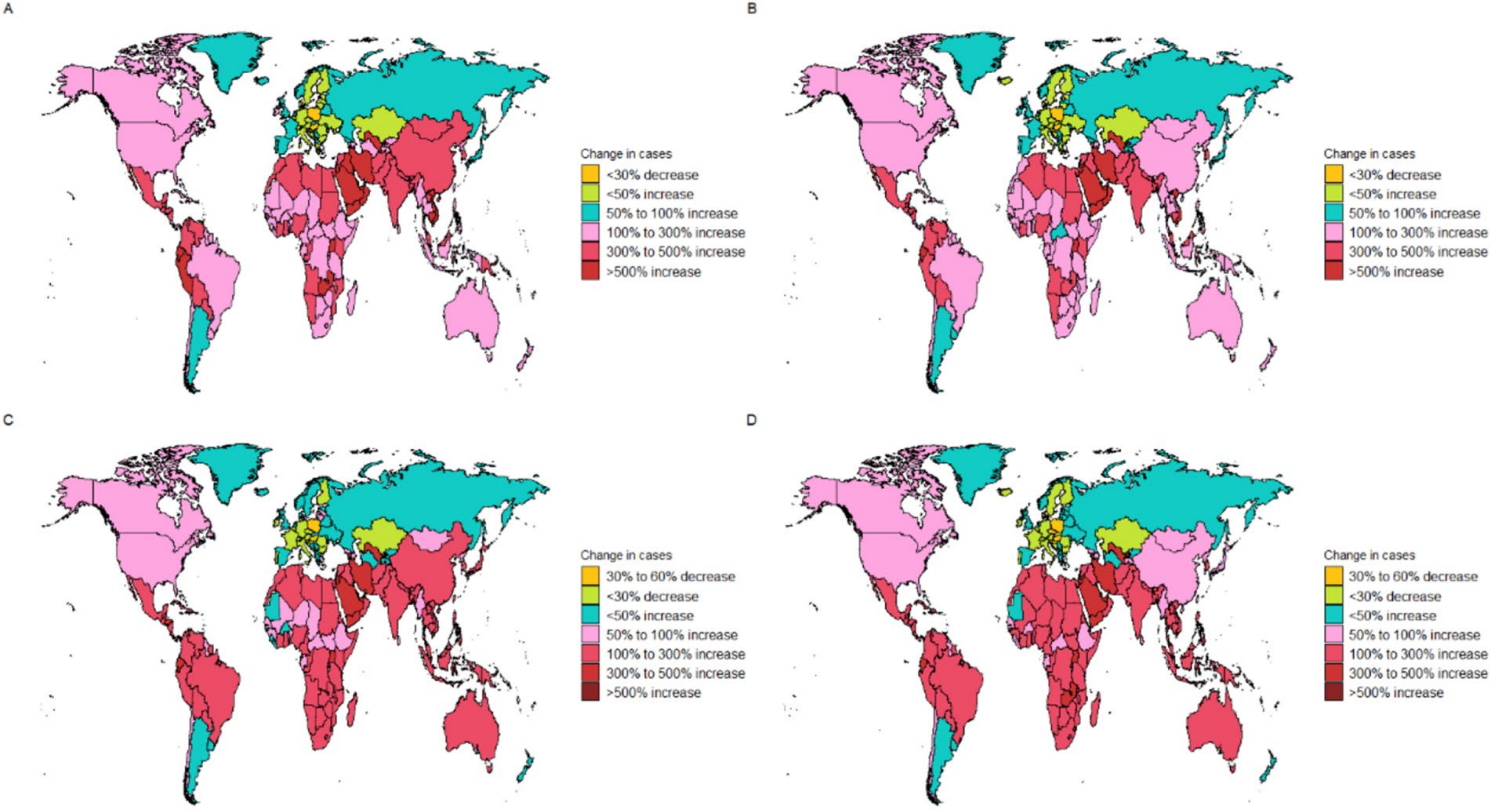
Change cases of thyroid cancer for both sexes in 204 countries and territories. **(A)** Change prevalence cases. **(B)** Change incidence cases. **(C)** Change deaths cases. **(D)** Change DALYs.

Between 1990 and 2021, the global prevalence of thyroid cancer showed an upward trend. Among them, the increase in the number of prevalence cases in Saudi Arabia was the most remarkable, with a staggering 1,401% increase, while that in Poland decreased by 7% (Figure 6A). Similarly, the United Arab Emirates had the highest increase in the number of incidence cases, rising by 1,317%, while Poland and Hungary decreased by 11 and 12%, respectively, (Figure 6B). In terms of the number of death cases, Ecuador had the largest increase, rising by 481%, while Hungary decreased by 40% (Figure 6C). Finally, Ecuador also witnessed the most significant increase in the number of DALYs, with a 410% increase, while Hungary decreased by 40% (Figure 6D).

### Age and gender patterns

**Figure 7:**
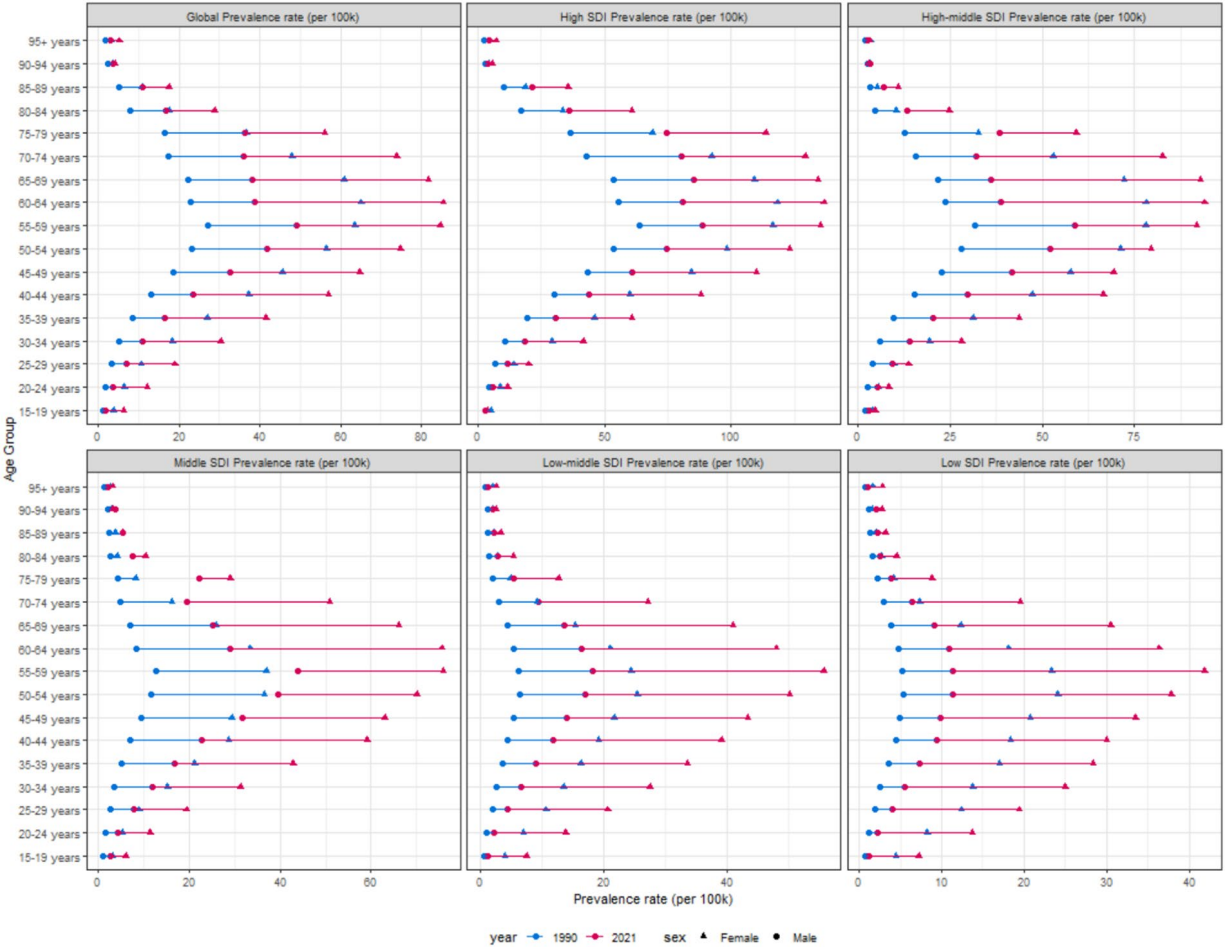
Age-standardized prevalence rates of thyroid cancer by sex, age group, and socio-demographic index, 1990 and 2021.

In 2021, globally, the ASPR of thyroid cancer gradually increased with age, peaked at the age of 55–59 years, and then gradually decreased. At all age groups, the ASPR in females was consistently higher than that in males (Figure 7 and Supplementary Figure S1). Similarly, the trend of the ASIR of global thyroid cancer in 2021 was similar to that of the ASPR (Supplementary Figure S2).

Compared with 1990, the ASDR of thyroid cancer in 2021 showed no significant changes in both males and females. In the population under 50 years old, both men and women had an extremely low ASDR for thyroid cancer, which strongly indicates that thyroid cancer has a favorable prognosis in young people. However, in the population over 50 years old, the ASDR gradually increased with age, and the ASDR in females was slightly higher than that in males (Supplementary Figure S3). The DALYs also indicated that, compared with 1990, there was a slight upward trend in 2021, and the age - standardized DALYs rates in females was still higher than that in males (Supplementary Figure S4).

### Future projections of the global burden of thyroid cancer

**Figure 8:**
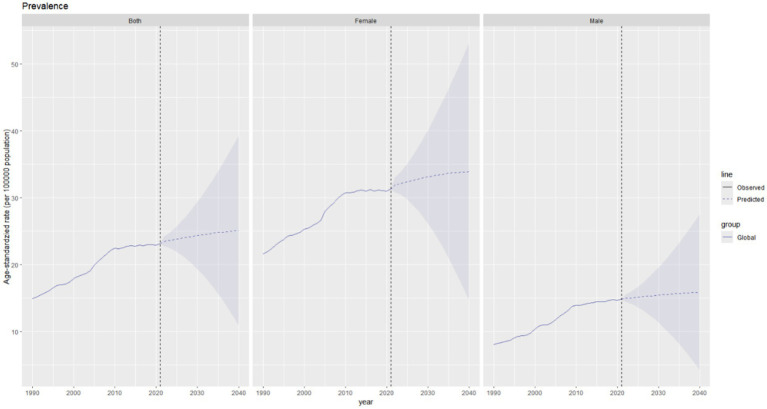
Future projections of the global burden of thyroid cancer prevalence.

It is projected that from 2021 to 2040, the global burden of thyroid cancer will continue to change, with varying trends across different indicators. The total ASPR of thyroid cancer in both men and women globally is expected to increase, rising from approximately 23 per 100,000 population in 2021 to around 25 per 100,000 population in 2040, representing an increase of approximately 9% over two decades (Figure 8). The prevalence in men is predicted to remain relatively stable, increasing slightly from about 15 per 100,000 population in 2021 to 16 per 100,000 population in 2040. In contrast, the number of female patients is expected to experience a more significant increase, rising from approximately 32 per 100,000 population in 2021 to around 35 per 100,000 population in 2040, indicating a steeper upward trend.

**Figure 9:**
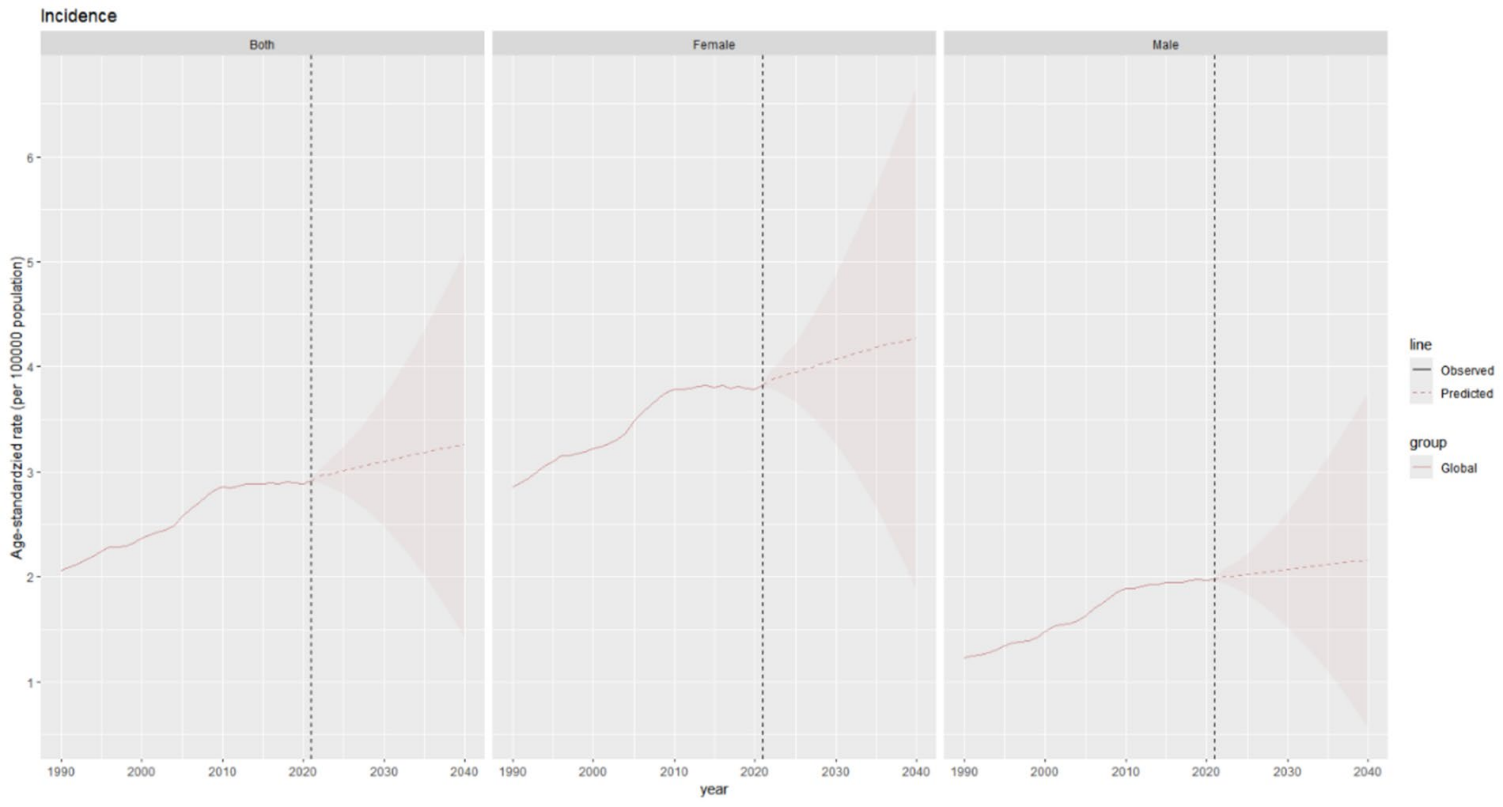
Future projections of the global burden of thyroid cancer incidence.

The global incidence of thyroid cancer shows a significant upward trend in the combined data of both sexes. The ASIR is expected to increase from approximately 2.9 per 100,000 population in 2021 to around 3.3 per 100,000 population in 2040. The upward trend of the incidence rate in females is predicted to be more pronounced than that in males (Figure 9).

**Figure 10:**
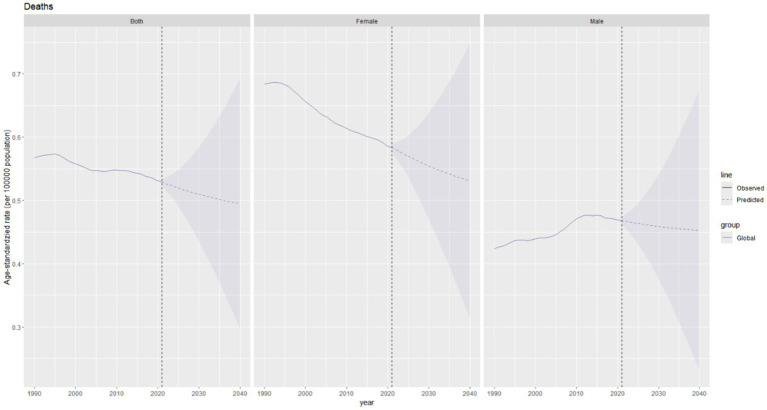
Future projections of the global burden of thyroid cancer deaths.

The total ASDR of thyroid cancer is expected to decline slightly, from approximately 0.54 per 100,000 population in 2021 to around 0.49 per 100,000 population in 2040 (Figure 10).

**Figure 11:**
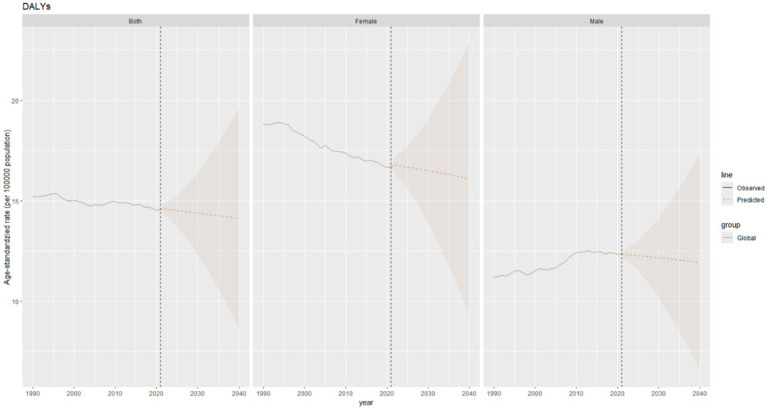
Future forecasts of global burden of thyroid cancer DALYs.

The age–standardized DALY rate of thyroid cancer is projected to decrease slightly in the combined data of both genders, declining marginally from 14.6 per 100,000 population in 2021 to approximately 14.2 per 100,000 population in 2040. The DALY rates in both males and females show a downward trend (Figure 11).

## Discussion

This study comprehensively evaluated the global burden of thyroid cancer from 1990 to 2021, clearly presenting trends in prevalence, incidence, mortality, and DALYs. Globally, the burden of thyroid cancer is remarkable, with distinct differences in regions, age groups, and genders.

Temporal trends in thyroid cancer ASPR and ASIR varied by SDI, reflecting distinct epidemiological transition stages. High-SDI regions showed a biphasic pattern: an initial upward trend followed by a decline, suggesting effective prevention/management interventions. In contrast, lower-SDI regions exhibited sustained increases, with middle-SDI areas showing the steepest rise. This trend may reflect the complex interplay of multiple factors. These include advancements in medical examination techniques, which enhance diagnostic capabilities and lead to a higher detection rate. The proactive participation in physical check-ups and cancer screenings, as well as the extension of life expectancy, expand the high-risk population. Additionally, there is an increasing exposure to risk factors associated with rapid urbanization and lifestyle changes. In modern life, increased stress, poor dietary habits, weight gain, and changes in hormone levels (such as estrogen levels) may all be related to the increased burden of thyroid cancer (19–22).

The simultaneous analysis of ASPR and ASIR provides complementary insights. ASIR, as a marker of new cases, highlights etiological drivers and intervention efficacy; for example, the post-2009 decline in ASIR in high-SDI regions suggests successful implementation of prevention strategies targeting modifiable risks (e.g., radiation exposure) and overdiagnosis reduction (23, 24). Conversely, ASPR, which integrates incidence and survival, is critical for healthcare resource planning. The plateau in ASPR in high-SDI regions may indicate a steady state of prevalent cases managed through long-term follow-up systems, whereas the continuous rise in ASPR in middle-SDI regions underscores the urgent need for expanded clinical capacity to address growing patient populations. In low-SDI regions, the discrepancy between rising ASIR and stable ASPR may reflect limited access to diagnosis and treatment, leading to shorter survival and lower prevalence despite increasing incidence. Together, these metrics reveal that high-SDI regions have transitioned from a phase of diagnostic expansion to one of quality improvement, while lower-SDI regions remain challenged by both emerging risk factors and inadequate healthcare infrastructure.

Compared with previous research based on the GBD database, this study not only updates the time span to 2021 but also adopts a more comprehensive analysis dimension. Although previous studies have indicated an upward trend in the incidence of thyroid cancer (25), this study further reveals unique change patterns in different SDI regions. In high-SDI regions, the incidence rate shows a downward trend after an initial increase, which may be attributed to effective prevention and management strategies (26, 27). In other SDI regions, the incidence rate continues to rise, indicating that these regions still face challenges in the prevention and control of thyroid cancer (5). Meanwhile, the detailed analysis of age and gender patterns in this study complements the deficiencies of previous research, clarifying the differences in the burden of thyroid cancer among different age groups and genders (28, 29). We have found that women are more likely to develop thyroid cancer, and the incidence rate in women is increasing at a faster pace. Previous studies have also shown that the thyroid cancer incidence rate in women is three times higher than that in men (30). This is largely explained by other factors commonly present in women, such as the expression of estrogen receptors (ER) in thyroid cancer tissues. Estrogen itself, or its metabolites, particularly the enhanced 2-hydroxylation reaction, may be related to the development of thyroid cancer (31, 32). Some more consistent epidemiological findings—such as autoimmune diseases (33), infertility and the use of fertility drugs (34, 35), recent pregnancy, irregular menstrual cycles (36), and hysterectomy or surgical menopause (37, 38)—may provide etiological clues. Such findings warrant further exploration.

Notably, the updated GBD 2021 results reveal critical divergences from prior GBD assessments. Compared to the GBD 2017 estimates (39), the global age-standardized incidence rate (ASIR) of thyroid cancer in 2021 (6.9 per 100,000) reflects a 24.1% increase from 2017 (5.56 per 100,000), yet this growth rate has markedly decelerated (annualized increase: 1.2% during 2017–2021 vs. 2.8% during 1990–2017). Regionally, high-SDI areas exhibited contrasting trends: the ASIR in North America declined by 7.3% between 2017 and 2021 (from 15.2 to 14.1 per 100,000), whereas GBD 2017 projected continued growth (40). Conversely, low-middle-SDI regions demonstrated accelerated incidence growth (3.5% annual increase post-2017 vs. 2.1% historically), exceeding prior predictions. Mortality patterns also diverged; the 2021 global death rate (0.5 per 100,000) showed a 12% reduction from GBD 2017 estimates (0.57 per 100,000) (41), largely driven by improved survival in high-SDI countries. These discrepancies may stem from methodological refinements in GBD 2021, including enhanced cancer registry coverage (now incorporating 48% more population-based registries in low-income regions) (8) and recalibrated DisMod-MR 2.1 models addressing overdiagnosis bias (42).

In addition, we applied advanced statistical methods to predict future trends in thyroid cancer. Over the next 20 years, the burden of thyroid cancer will exhibit a complex “two increases and two decreases” pattern. Specifically, the ASPR is expected to increase by 9%, the ASIR will rise by 13.8%, the ASDR will decrease by 9.3%, and the age - standardized DALY rate will decline by 2.7%. Policies should focus on three key areas: precise prevention and control, standardized diagnosis and treatment, and survival care. Particular attention must be given to the needs of women and long-term cancer survivors to ensure the health benefits of the entire population as the disease burden rises.

Given the ongoing global increase in the burden of thyroid cancer and the significant regional heterogeneity (with high-SDI regions facing the risk of diagnosing clinically irrelevant thyroid carcinomas, while low-SDI regions struggle with higher mortality and disease burden due to limited diagnostic and treatment resources), public health interventions must adopt a dual-track approach. In high-resource regions, risk-based screening and active monitoring strategies should be implemented, such as adhering to American Thyroid Association (ATA) guidelines to limit imaging screening and monitor low-risk microcarcinomas, in order to curb the detection of indolent tumors. In resource-limited areas, priority should be given to improving access to basic diagnosis and treatment, such as strengthening palpation skills at the grassroots level, promoting ultrasound-guided fine needle aspiration techniques, and ensuring coverage of surgery and radioactive iodine therapy, to reduce preventable deaths in high-burden regions like East Africa. At the global level, cancer registration systems need to be enhanced to track disease patterns, iodine nutrition management and radiation protection research should be strengthened, and long-term health management for high-risk female populations should be prioritized, in order to achieve precise prevention and control of the disease burden.

The strength of this study lies in the utilization of the latest GBD 2021 data, ensuring the timeliness and accuracy of the research results. Through multi - dimensional stratified analysis, the relationships between the burden of thyroid cancer and gender, age, region, and SDI are comprehensively explored, providing rich information for a deep understanding of the epidemiological characteristics of the disease. In addition, the application of advanced statistical methods to predict future trends provides a basis for formulating long - term prevention and control strategies. However, the study also has certain limitations. Since the data are sourced from the GBD database, they may be affected by errors in the data collection and collation process. Moreover, although the associations between multiple factors and the burden of thyroid cancer have been analyzed, some potential factors, such as specific gene mutations and exposure to environmental pollutants, have not been explored in depth for their impacts on the disease.

In conclusion, this study provides a comprehensive analysis of the global burden of thyroid cancer from 1990 to 2021, revealing temporal trends, geographical differences, and sex - related patterns. These findings deepen our understanding of the disease’s epidemiology and lay a foundation for targeted public health interventions.

## Data Availability

The raw data supporting the conclusions of this article will be made available by the authors, without undue reservation.
