# Supplementary material for: Temporal trends and geographic disparities in thyroid cancer burden: a global analysis from 1990 to 2021
Source: Front Nutr. 2025 Jul 16;12:1613737. doi: 10.3389/fnut.2025.1613737 (PMC12307148; doi:10.3389/fnut.2025.1613737)
Supplement: Supplementary file 1 [file Table_1.doc]

**Supplementary materials**

**Table S1:** Prevalence cases and ASPR of thyroid cancer in 1990 and 2021 and its trends, by 204 countries and territories.

**Table S2:** Incidence cases and ASIR of thyroid cancer in 1990 and 2021 and its trends, by 204 countries and territories.

**Table S3:** Deaths cases and ASDR of thyroid cancer in 1990 and 2021 and its trends, by 204 countries and territories.

**Table S4:** DALYs and age standardized DALYs rate of thyroid cancer in 1990 and 2021 and its trends, by 204 countries and territories.

**Figure S1:** Temporal trends in age- and sex-stratified prevalence rates (per 100,000 population) for thyroid cancer , comparing 1990 and 2021.

**Figure S2：**Temporal trends in age- and sex-stratified incidence rates (per 100,000 population) for thyroid cancer , comparing 1990 and 2021.

**Figure S3：**Temporal trends in age- and sex-stratified deaths rates (per 100,000 population) for thyroid cancer , comparing 1990 and 2021.

**Figure S4：**Temporal trends in age- and sex-stratified DALY rates (per 100,000 population) for thyroid cancer , comparing 1990 and 2021.

**Methods S1**: Detailed implementation of the BAPC model.

**Methods S2**: Estimation of EAPC and sensitivity analyses.

**Table S1: Prevalence cases and ASPR of thyroid cancer in 1990 and 2021 and its trends, by 204 countries and territories.**

|  | **1990** | | **2021** | |  |
| --- | --- | --- | --- | --- | --- |
| **location** | **Number** | **ASR** | **Number** | **ASR** | **EAPC_95%CI** |
| High-income North America | 100673.1 (97445.8-103313) | 32.1 (31.1-32.9) | 237732 (226609-247666.5) | 45.5 (43.6-47.3) | 1.23 (1.05 to 1.42) |
| Canada | 7603.2 (6530.5-8806.3) | 24.7 (21.2-28.5) | 15490.1 (12643.8-18782.7) | 29.1 (23.6-35.4) | 0.32 (0.04 to 0.59) |
| Greenland | 6.3 (4.1-8.8) | 11.7 (8-16.1) | 10.3 (7-14.5) | 13.7 (9.6-19) | 1.78 (1.14 to 2.41) |
| United States of America | 93061.3 (90184.3-95521.6) | 32.9 (31.9-33.7) | 222227.8 (212085.4-231109.7) | 47.4 (45.5-49.3) | 1.31 (1.12 to 1.5) |
| Australasia | 4599.9 (4076.9-5210.9) | 20.6 (18.3-23.3) | 16211.2 (13090.8-19620.8) | 38.9 (31.4-47.6) | 2.87 (2.29 to 3.47) |
| Australia | 3886.6 (3373.7-4439.9) | 20.9 (18.2-23.8) | 14705.4 (11776.1-17994.6) | 42.1 (33.5-52.3) | 3.11 (2.49 to 3.73) |
| New Zealand | 713.3 (601.7-852.3) | 19.5 (16.4-23.2) | 1505.8 (1217.6-1776.7) | 22.7 (18.4-27) | 1.16 (0.64 to 1.67) |
| High-income Asia Pacific | 55344.6 (51670.9-60632.8) | 26.9 (25.1-29.6) | 110959.1 (98931.9-129334.7) | 37.1 (33.2-43.8) | 1.42 (0.91 to 1.93) |
| Brunei Darussalam | 35.4 (24.3-52.6) | 21.1 (14.7-30.6) | 137.8 (102.5-186.1) | 28.6 (21.3-38.3) | 1.55 (1.38 to 1.72) |
| Japan | 47389.6 (44881.3-50006.4) | 28.8 (27.3-30.4) | 76047.2 (69549.5-81980.6) | 35.9 (33.5-38.6) | 0.81 (0.54 to 1.09) |
| Singapore | 673.4 (577.8-778.5) | 23 (19.7-26.6) | 2261.7 (1870.2-2807.9) | 26.7 (22.1-33.2) | 0.81 (0.3 to 1.33) |
| Republic of Korea | 7246.2 (5101.1-12383.7) | 17.8 (12.6-31) | 32512.4 (24249.4-49154.2) | 40.7 (30-61.1) | 3.47 (2.32 to 4.64) |
| Western Europe | 148253.3 (140945-155876.6) | 31.1 (29.5-32.7) | 211123.9 (193366-229245.3) | 32.7 (29.9-35.5) | 0.51 (0.09 to 0.94) |
| Andorra | 13.8 (9.5-19.4) | 22.5 (15.5-31.8) | 34.5 (22.6-48.9) | 25.4 (16.6-35.9) | 0.78 (0.46 to 1.11) |
| Austria | 4098.8 (3638-4597) | 42.1 (37.4-47.5) | 5653.1 (4670-6782.6) | 41.1 (33.7-49.4) | 0.08 (-0.17 to 0.33) |
| Belgium | 3136.8 (2694.9-3707.5) | 24.8 (21.2-29.1) | 4668.2 (3772.8-5679.4) | 28.5 (23.2-34.7) | 0.65 (0.33 to 0.98) |
| Cyprus | 153.9 (112.2-199.1) | 18.8 (13.7-24.4) | 419.8 (312-591.1) | 22.6 (16.8-32) | 0.69 (0.22 to 1.16) |
| Denmark | 851.6 (729.4-997.6) | 13.3 (11.3-15.6) | 1130.6 (942.9-1363) | 12.7 (10.7-15.4) | 0.38 (-0.19 to 0.95) |
| Finland | 1626.7 (1392.3-1895.5) | 25.8 (22.1-30.3) | 2147.6 (1753.2-2625.9) | 25.3 (20.5-30.9) | 0.52 (-0.18 to 1.23) |
| France | 30926.2 (27480.4-34702.3) | 45.7 (40.5-51.4) | 53648.4 (44287-64054.2) | 58.8 (48.4-70.9) | 1.26 (0.51 to 2.02) |
| Germany | 30876.6 (26964.3-35523) | 30.1 (26.2-34.7) | 39078.1 (31979.2-46847) | 31.2 (25.4-37.4) | 0.65 (0.21 to 1.09) |
| Greece | 2428.2 (2204.5-2666.5) | 18.2 (16.5-19.9) | 3432.8 (3017.5-3895.1) | 22 (19.4-24.9) | 0.65 (0.41 to 0.89) |
| Iceland | 166.2 (143.2-191.9) | 64 (54.9-74.2) | 251.2 (205.4-311.5) | 55.2 (45-68.6) | 0.05 (-0.62 to 0.73) |
| Ireland | 741.6 (640.8-838) | 20 (17.3-22.6) | 1548.4 (1287.3-1861.8) | 23.3 (19.3-28.2) | 1.47 (1.1 to 1.85) |
| Israel | 1058.9 (901.4-1257.4) | 23.2 (19.6-27.6) | 2809.8 (2305.5-3441.1) | 26.8 (22-32.8) | 0.76 (0.16 to 1.36) |
| Italy | 39847.1 (36862.8-43553.4) | 53.4 (49.4-58.4) | 43993.3 (39726.9-48617.3) | 45.6 (41.2-50.5) | -0.47 (-0.84 to -0.1) |
| Luxembourg | 152 (137.9-167.6) | 31.6 (28.7-34.8) | 261.7 (227.4-305.6) | 28.2 (24.6-32.8) | -0.05 (-0.42 to 0.32) |
| Malta | 93.3 (79.1-110.8) | 22 (18.7-26.2) | 179.8 (144.1-220.9) | 27.1 (21.6-33.7) | 0.89 (0.36 to 1.42) |
| Monaco | 10.9 (8.2-14.3) | 24.1 (18.2-31.5) | 20.9 (15.1-27.5) | 36.2 (25.4-49.9) | 1.51 (1.35 to 1.68) |
| Netherlands | 2217 (1967.4-2517.2) | 12.6 (11.1-14.3) | 4426.2 (3700-5214.9) | 17.4 (14.4-20.6) | 1.75 (1.43 to 2.07) |
| Norway | 982.8 (907.4-1066.7) | 18.4 (16.9-19.9) | 1796.5 (1572.8-2012.5) | 22.3 (19.6-25) | 1.12 (0.59 to 1.66) |
| Portugal | 3133.8 (2699.5-3631.9) | 24.9 (21.4-28.8) | 5219.6 (4112.4-6487.5) | 30.3 (24-37.6) | 0.69 (0.29 to 1.1) |
| San Marino | 12 (9.6-15) | 40.7 (32.7-50.7) | 17.8 (10.3-26.4) | 35.7 (20.4-52.8) | 0.48 (0.04 to 0.92) |
| Spain | 9522 (8508.1-10657.4) | 20.7 (18.5-23.1) | 15246.5 (12802.5-18026.1) | 21.2 (17.7-25.3) | 0.37 (-0.02 to 0.75) |
| Sweden | 1820.9 (1572.5-2113.2) | 15.8 (13.6-18.3) | 2344.3 (1924-2838.8) | 15.2 (12.6-18.4) | 0.66 (0.27 to 1.04) |
| Switzerland | 1731.3 (1530.5-1957.3) | 19.9 (17.6-22.4) | 2167.8 (1809.8-2556.9) | 15.6 (13-18.5) | -0.36 (-1.18 to 0.46) |
| United Kingdom | 12529.3 (12180.9-12866.4) | 17.4 (16.9-17.8) | 20441.3 (19496.9-21220.1) | 21.2 (20.3-22) | 1.27 (1.05 to 1.49) |
| Southern Latin America | 6690 (5949.1-7523.9) | 14.1 (12.6-15.9) | 15500.3 (13535.1-17754.5) | 19.7 (17.1-22.6) | 1.21 (1 to 1.42) |
| Argentina | 4329 (3768.8-5008.1) | 13.4 (11.7-15.5) | 8653.2 (7102.7-10495.7) | 16.9 (13.9-20.5) | 0.85 (0.61 to 1.09) |
| Chile | 1813.6 (1499.9-2157.4) | 15.6 (13-18.6) | 5743.6 (4873.3-6879.6) | 24.7 (20.9-29.7) | 1.71 (1.49 to 1.92) |
| Uruguay | 547 (439.5-666.7) | 16.2 (13-19.8) | 1102.7 (905.5-1354.3) | 26.4 (21.5-32.4) | 1.72 (1.52 to 1.92) |
| Eastern Europe | 50163.3 (47847.6-52997.6) | 18.9 (18.1-20) | 75906.4 (68504.6-84343.5) | 25.9 (23.3-28.8) | 1.47 (0.97 to 1.97) |
| Belarus | 2385.7 (1950.2-2906.6) | 20.1 (16.2-24.3) | 4397.4 (3418.9-5632.1) | 31.4 (24.4-40.6) | 0.93 (0.41 to 1.45) |
| Estonia | 454.1 (349.3-578.1) | 24.7 (19-31.5) | 623.2 (471.5-794.6) | 31.4 (23.5-40.1) | 0.97 (0.4 to 1.54) |
| Latvia | 641.4 (492.4-826.1) | 20.3 (15.4-26.2) | 935.6 (706-1222.6) | 31.6 (23.8-41.6) | 1.01 (0.67 to 1.35) |
| Lithuania | 1067.7 (838-1338.9) | 25.8 (20-32.5) | 1295 (1010.8-1615.6) | 30.7 (23.5-38) | 0.28 (-0.23 to 0.78) |
| Republic of Moldova | 595.3 (530.3-666.4) | 12.8 (11.4-14.2) | 986.5 (835.2-1172.9) | 18.4 (15.6-21.8) | 1.2 (0.97 to 1.43) |
| Russian Federation | 34089.9 (32999.8-35269.7) | 19.5 (18.9-20.2) | 53501.5 (48453.2-58912.7) | 26.5 (24-29.2) | 1.7 (1.11 to 2.28) |
| Ukraine | 10929.2 (9126.7-13489.3) | 17 (14.2-20.8) | 14167.1 (9646.7-20161) | 22.8 (15.3-32.5) | 0.85 (0.42 to 1.29) |
| Central Europe | 34174.4 (32484.3-36024.9) | 23.6 (22.4-24.8) | 37892.2 (34073.8-41467.7) | 21.9 (19.7-24) | -0.33 (-0.52 to -0.14) |
| Albania | 285.7 (200.7-388.8) | 10.7 (7.4-14.5) | 590.3 (398.7-870.4) | 16.2 (10.8-23.8) | 1.88 (1.58 to 2.19) |
| Bosnia and Herzegovina | 608.4 (438.8-734.7) | 12.7 (9.2-15.3) | 794.6 (578.3-1076.7) | 15.3 (11.2-20.8) | 0.66 (0.48 to 0.84) |
| Bulgaria | 1673 (1401.2-1992.3) | 14.7 (12.4-17.4) | 1828.4 (1426.8-2344.4) | 17 (13.3-21.8) | 0.86 (0.5 to 1.22) |
| Croatia | 2238.1 (1919.4-2622) | 36 (31.2-41.9) | 2009.7 (1611-2471.4) | 29.2 (23.2-35.9) | -0.5 (-0.79 to -0.21) |
| Czechia | 4265.7 (3601.2-5084.1) | 34.2 (28.9-41) | 5261.8 (3972.2-6879.2) | 32.4 (24.3-42.7) | -0.11 (-0.49 to 0.27) |
| Hungary | 3734.6 (3234.5-4259.1) | 28.6 (24.8-32.4) | 3571.9 (2917.5-4278.8) | 24.1 (19.5-28.7) | -0.94 (-1.17 to -0.71) |
| Montenegro | 168.2 (132.7-219.1) | 25.6 (20.1-33.3) | 269 (208.9-341.7) | 30.8 (24.2-39.3) | 1.03 (0.9 to 1.16) |
| North Macedonia | 293.4 (220.3-346.2) | 14.1 (10.6-16.7) | 538.9 (404.6-695.3) | 16.7 (12.5-21.8) | 0.6 (0.38 to 0.81) |
| Poland | 13443.7 (12416.9-14414.2) | 31.7 (29.3-33.9) | 12445.3 (10935.8-13985.6) | 22.6 (19.8-25.2) | -1.26 (-1.7 to -0.82) |
| Romania | 3882.7 (3282.8-4578.8) | 14.4 (12.1-17) | 5224.9 (4024-6742.8) | 18 (13.8-23.3) | 0.37 (0.1 to 0.64) |
| Serbia | 1288.8 (964.5-1757.2) | 11 (8.2-15) | 2282.8 (1623.7-3156.4) | 17.1 (12.2-23.9) | 1.45 (1.27 to 1.64) |
| Slovakia | 1174.1 (917.1-1463.9) | 20.5 (15.9-25.6) | 1845.2 (1399.5-2524.5) | 22.8 (17.1-31.5) | 0.39 (0.3 to 0.49) |
| Slovenia | 571.3 (481.3-674.3) | 24.1 (20.2-28.4) | 677.8 (490.9-911.3) | 19.2 (13.8-25.8) | -0.26 (-0.66 to 0.14) |
| Central Asia | 6945.8 (6347.8-7593.4) | 12.7 (11.6-13.9) | 13021.3 (11400.3-14782.4) | 13.4 (11.7-15.2) | 0.16 (-0.61 to 0.93) |
| Armenia | 381.6 (280.5-498.4) | 12 (8.9-15.5) | 1117.7 (836.8-1476.2) | 28.1 (20.9-37.4) | 3.4 (2.94 to 3.85) |
| Azerbaijan | 444.5 (313.8-640.5) | 7.3 (5.3-10.4) | 1196 (806.6-1719.5) | 9.6 (6.5-13.8) | 0.98 (0.69 to 1.27) |
| Georgia | 885.4 (691.7-1126.1) | 14.5 (11.4-18.5) | 1586.4 (1260.9-1979.8) | 32.4 (25.3-40.4) | 2.88 (1.67 to 4.11) |
| Kazakhstan | 3909 (3410.8-4441.2) | 26.5 (23.1-30.2) | 4808.1 (3902.2-5748.9) | 23.6 (19.1-28.2) | -0.63 (-1.45 to 0.19) |
| Kyrgyzstan | 608.3 (468.8-805.7) | 17 (13.2-22.3) | 1223.8 (856.4-1674.9) | 19.8 (14-26.9) | 0.87 (-0.11 to 1.85) |
| Mongolia | 111 (77.1-161.4) | 8.2 (5.7-12) | 446.3 (328.5-622) | 13.8 (10.2-19.1) | 1.67 (1.19 to 2.15) |
| Tajikistan | 4.1 (2.8-5.7) | 0.1 (0.1-0.2) | 8.4 (5.7-12.2) | 0.1 (0.1-0.1) | -1 (-1.27 to -0.72) |
| Turkmenistan | 299 (264.2-338.5) | 11.8 (10.5-13.4) | 688.3 (518-930.8) | 13.6 (10.2-18.3) | 0.44 (-0.54 to 1.43) |
| Uzbekistan | 303 (222.5-399.5) | 2.1 (1.6-2.8) | 1946.3 (1420.5-2674.2) | 5.7 (4.2-7.8) | 3.33 (2.58 to 4.09) |
| Central Latin America | 11472.2 (11049.9-11916.4) | 10.2 (9.8-10.6) | 58071 (51803-65647.6) | 21.8 (19.4-24.6) | 2.39 (2.28 to 2.5) |
| Colombia | 3575.8 (3238.4-3947.4) | 14.9 (13.5-16.4) | 16167.8 (12936.4-20259.7) | 29.5 (23.7-37) | 2.21 (1.94 to 2.48) |
| Costa Rica | 422.1 (375.5-479.2) | 18.8 (16.8-21.2) | 1490.6 (1243.1-1740.9) | 27.3 (22.8-31.9) | 0.61 (0.25 to 0.97) |
| Guatemala | 354.1 (327.1-384.2) | 6.8 (6.3-7.4) | 1887.4 (1588.3-2228.4) | 14.5 (12.2-17) | 2.01 (1.76 to 2.27) |
| Honduras | 82.4 (64-106.6) | 2.8 (2.2-3.6) | 463.8 (309.4-652.9) | 5.9 (4-8.3) | 2.33 (2.08 to 2.58) |
| Mexico | 5265.1 (5100-5426) | 9.2 (8.9-9.5) | 29413.9 (25547-33675.2) | 21.3 (18.5-24.4) | 2.66 (2.56 to 2.76) |
| Nicaragua | 140.9 (118.6-177.5) | 6.3 (5.3-7.9) | 785.5 (581.8-1002.2) | 13.5 (10-17.2) | 2.91 (2.7 to 3.12) |
| Panama | 292.9 (248.2-342.9) | 14.6 (12.6-16.9) | 972.2 (771.6-1220.6) | 22 (17.5-27.6) | 0.97 (0.44 to 1.51) |
| El Salvador | 356.3 (283.2-405.8) | 10 (7.7-11.4) | 1515.5 (1165.3-1939.1) | 24.9 (19.2-31.9) | 3.15 (2.99 to 3.31) |
| Venezuela (Bolivarian Republic of) | 982.6 (900.6-1064.1) | 7.6 (7-8.3) | 5374.2 (3953.9-6996) | 17.4 (12.8-22.5) | 2.6 (2.41 to 2.78) |
| Andean Latin America | 2640.4 (2211.1-3138.8) | 9.8 (8.1-11.5) | 18047.3 (13837.2-22793) | 28.1 (21.6-35.6) | 3.55 (3.34 to 3.77) |
| Bolivia (Plurinational State of) | 530.2 (330.7-743.3) | 12.2 (7.5-17.1) | 2559.7 (1725.5-3690.3) | 23.9 (16-34.4) | 2.17 (2.1 to 2.25) |
| Ecuador | 574.9 (514.2-638.4) | 8.1 (7.2-8.9) | 5186.6 (3913.1-6649.1) | 29.8 (22.5-38.1) | 4.41 (3.72 to 5.1) |
| Peru | 1535.3 (1234.7-1896.3) | 9.9 (7.9-12.2) | 10301 (7140.8-14364.4) | 28.6 (20-40.1) | 3.57 (3.41 to 3.74) |
| Caribbean | 3118.1 (2873.6-3378.8) | 10.6 (9.8-11.5) | 9357.3 (8102.5-10834.1) | 17.8 (15.4-20.6) | 1.86 (1.69 to 2.04) |
| Antigua and Barbuda | 6.6 (5.9-7.4) | 12.9 (11.5-14.5) | 26.2 (23.8-29.1) | 23.2 (21.1-25.7) | 2.32 (1.9 to 2.74) |
| Barbados | 31.8 (29-35) | 13.1 (11.9-14.5) | 88.5 (68.7-113.9) | 21 (16.4-27.1) | 1.8 (1.58 to 2.02) |
| Belize | 5.1 (4.6-5.6) | 4.4 (4-4.8) | 40.6 (35-46.5) | 10.5 (9-12) | 3.05 (2.81 to 3.29) |
| Bermuda | 13.5 (12-15.4) | 20.3 (17.9-23) | 37.2 (29.6-47.2) | 37.7 (30.2-47.7) | 2 (1.73 to 2.28) |
| Bahamas | 28.6 (25.3-32.1) | 13.8 (12.3-15.6) | 107.4 (83.8-138.3) | 23.6 (18.3-30.3) | 2.31 (2.04 to 2.58) |
| Cuba | 1418.6 (1278-1582.2) | 13.4 (12-14.9) | 4297.6 (3546.4-5103.3) | 26.1 (21.6-31) | 2.22 (1.9 to 2.55) |
| Dominica | 4.4 (3.7-5.3) | 7.8 (6.6-9.3) | 9 (6.8-11.6) | 11.1 (8.5-14.4) | 1.31 (1.16 to 1.46) |
| Dominican Republic | 328.3 (259.4-400.1) | 6.3 (5-7.7) | 1295 (943.6-1761) | 11.8 (8.6-16.1) | 2.31 (2.15 to 2.47) |
| Grenada | 9.4 (8-11) | 14.7 (12.4-17.2) | 24.9 (20.4-30.3) | 20.8 (17.1-25.3) | 1.48 (1.08 to 1.88) |
| Guyana | 30.2 (25.5-35.2) | 5.7 (4.8-6.6) | 90.3 (66.5-123.4) | 11.9 (8.8-16.2) | 2.58 (2.38 to 2.78) |
| Haiti | 276.5 (171-405.4) | 6 (3.8-8.7) | 847.7 (561.7-1253.5) | 7.9 (5.2-11.6) | 1.14 (1 to 1.29) |
| Jamaica | 163.6 (144.7-184.9) | 9.4 (8.3-10.6) | 612.3 (439.1-837.9) | 19.9 (14.3-27.3) | 2.46 (2.08 to 2.84) |
| Puerto Rico | 531.8 (468.8-601.9) | 14.9 (13.1-16.9) | 1044.9 (837.1-1284.3) | 23.4 (18.5-29) | 1.92 (1.58 to 2.26) |
| Saint Kitts and Nevis | 2.5 (2.3-2.8) | 7.9 (7.1-8.8) | 9.2 (7.4-11.3) | 11.6 (9.4-14.3) | 1.36 (1.01 to 1.7) |
| Saint Lucia | 14.4 (13.2-15.9) | 15.1 (13.7-16.7) | 54.4 (43.4-67.2) | 23.6 (18.8-29.1) | 1.43 (1.02 to 1.83) |
| Suriname | 24 (18.2-28.9) | 7.6 (5.8-9.1) | 70 (51-89.5) | 10.7 (7.9-13.6) | 1.47 (1.25 to 1.69) |
| Trinidad and Tobago | 103.3 (94.7-112.4) | 10.6 (9.7-11.5) | 337 (253.2-443.5) | 19.1 (14.3-25.2) | 2.08 (1.81 to 2.35) |
| Saint Vincent and the Grenadines | 12.1 (10.7-13.3) | 15.7 (13.9-17.4) | 39.1 (33.2-45.7) | 28.9 (24.6-33.8) | 2.14 (1.75 to 2.54) |
| United States Virgin Islands | 7.8 (6.1-9.8) | 7.4 (5.8-9.2) | 9.4 (6.7-13) | 8.7 (6.2-12.1) | 0.88 (0.73 to 1.04) |
| Tropical Latin America | 9237.1 (8742.4-9743.8) | 7.8 (7.4-8.2) | 33082.7 (31066.3-34983.6) | 12.6 (11.8-13.3) | 1.32 (1.13 to 1.51) |
| Brazil | 8945.4 (8472.7-9448.2) | 7.7 (7.3-8.2) | 31849 (29877.4-33749.4) | 12.4 (11.7-13.2) | 1.3 (1.1 to 1.5) |
| Paraguay | 291.7 (237.1-373.9) | 10.3 (8.4-13.3) | 1233.7 (897.5-1631.9) | 18.5 (13.5-24.6) | 1.93 (1.85 to 2.01) |
| East Asia | 95529.4 (76898.9-113229.6) | 8.5 (6.9-10.1) | 411402.4 (334530-513557.1) | 20.5 (16.8-25.6) | 3.16 (3 to 3.33) |
| China | 87081.7 (68622.2-104169) | 8.1 (6.4-9.7) | 388411 (311967.5-488388.4) | 20 (16.1-25.2) | 3.25 (3.07 to 3.42) |
| Democratic People's Republic of Korea | 2282 (1577.2-3150.5) | 11.2 (7.8-15.5) | 6113.5 (4082.9-8882.3) | 18.5 (12.5-26.9) | 1.84 (1.69 to 1.98) |
| Taiwan (Province of China) | 6165.7 (5562.9-6911.1) | 30.5 (27.7-33.9) | 16877.9 (14499.3-19222.4) | 50.2 (42.9-57.3) | 2.23 (1.84 to 2.63) |
| Southeast Asia | 45506 (36198.2-51840.3) | 12.7 (10.3-14.5) | 206164.7 (161849.8-244210.6) | 26.9 (21.1-31.8) | 2.33 (2.23 to 2.43) |
| Cambodia | 667.8 (411.8-897.7) | 10.5 (6.5-14) | 4042 (2477.3-5840.1) | 26 (15.9-37.5) | 2.95 (2.83 to 3.06) |
| Indonesia | 13920.1 (9317-17030.5) | 9.8 (6.6-12) | 55528.9 (35549.6-75127.1) | 18 (11.5-24.3) | 1.76 (1.64 to 1.87) |
| Lao People's Democratic Republic | 248.6 (131.7-363.3) | 9 (4.8-13.3) | 1113.2 (708-1567.2) | 17.4 (10.9-24.4) | 2.07 (2.01 to 2.13) |
| Malaysia | 2124.4 (1649.4-2603.9) | 15.8 (12.5-19.2) | 10236.3 (8425.4-12589.9) | 30.8 (25.5-37.6) | 2.16 (2.06 to 2.26) |
| Maldives | 9.3 (4.7-13.4) | 7.1 (3.7-10.2) | 50.8 (37.5-70.9) | 10 (7.4-13.1) | 1 (0.59 to 1.41) |
| Mauritius | 109.3 (99.7-118.5) | 12.1 (11-13.1) | 218 (190.8-242.1) | 12.4 (10.8-13.8) | 0.52 (-0.39 to 1.44) |
| Myanmar | 3120 (1819.9-4202.7) | 10 (6-13.4) | 10233.5 (6724.1-13916.4) | 17.9 (11.7-24.3) | 1.68 (1.57 to 1.78) |
| Philippines | 7925.7 (5899.6-9806.7) | 17.5 (12.8-21.8) | 29795.5 (23719.8-37321.9) | 28.4 (22.7-35.5) | 1.4 (1.29 to 1.5) |
| Sri Lanka | 2089.6 (1637-2530) | 14.6 (11.2-17.7) | 6653.9 (3998.8-9608.8) | 25.4 (15.3-36.5) | 1.73 (1.53 to 1.93) |
| Seychelles | 3.5 (2.8-4.5) | 6 (4.7-7.6) | 11 (8.9-13.6) | 8.5 (6.8-10.5) | 1.09 (0.75 to 1.43) |
| Thailand | 7774.4 (6153.5-10650.2) | 15.8 (12.4-21.9) | 30235.3 (21490.4-46346.7) | 31.9 (22.6-48.4) | 1.66 (1.23 to 2.1) |
| Timor-Leste | 33.3 (20-48.1) | 6.8 (4.1-9.8) | 132.6 (83.8-195.2) | 13.3 (8.2-19.8) | 2.46 (2.19 to 2.72) |
| Viet Nam | 7414.1 (5427.8-10243.6) | 14.7 (10.6-20.2) | 57626.3 (37939.1-77559.9) | 50.6 (33.6-67.4) | 4.68 (4.45 to 4.92) |
| Oceania | 308.3 (199-418.5) | 6.8 (4.5-9.3) | 960.2 (576-1382.1) | 8.6 (5.2-12.3) | 0.59 (0.48 to 0.7) |
| American Samoa | 4.5 (3.4-6.1) | 14.2 (10.6-19.2) | 13.5 (9.4-17.8) | 24.7 (17.4-32.2) | 1.92 (1.56 to 2.28) |
| Cook Islands | 1.7 (1.3-2.2) | 11.1 (8.5-13.9) | 3.7 (2.7-5.3) | 16.7 (12.1-25) | 0.72 (0.4 to 1.05) |
| Micronesia (Federated States of) | 3.8 (2.7-5.2) | 5.9 (4.2-8.1) | 10.3 (6.7-14.5) | 10.6 (6.9-15) | 1.91 (1.79 to 2.03) |
| Fiji | 114.9 (72-154.9) | 20.6 (12.8-27.7) | 244 (145.1-348) | 25.8 (15.4-36.7) | 0.55 (0.43 to 0.68) |
| Guam | 6.4 (5-11.2) | 5.9 (4.7-10.3) | 27.8 (20.9-32.8) | 14.8 (11.1-17.3) | 3.89 (2.81 to 4.99) |
| Kiribati | 0.1 (0.1-0.2) | 0.2 (0.1-0.4) | 0.3 (0.2-0.6) | 0.3 (0.2-0.6) | 0.73 (0.48 to 0.98) |
| Marshall Islands | 1.2 (1-1.6) | 4.9 (3.7-6.4) | 4.9 (3.1-7.3) | 9.3 (5.9-13.9) | 1.97 (1.8 to 2.14) |
| Nauru | 0.6 (0.4-0.8) | 7.9 (5.2-11.2) | 1.3 (0.7-1.9) | 14.9 (8.4-22) | 1.96 (1.86 to 2.06) |
| Niue | 0.2 (0.1-0.2) | 7.7 (5.4-10.6) | 0.3 (0.2-0.4) | 16.9 (11.1-22.6) | 1.92 (1.74 to 2.1) |
| Northern Mariana Islands | 2.5 (1.7-3.8) | 7.1 (5-10.2) | 7.5 (5.6-9.2) | 11.9 (9.1-14.4) | 1.82 (1.17 to 2.48) |
| Palau | 1.2 (0.9-1.7) | 9.9 (7.1-13.9) | 3.5 (2.5-4.8) | 14 (10.2-18.8) | 0.98 (0.86 to 1.11) |
| Papua New Guinea | 118.5 (62.4-190.3) | 4.2 (2.2-6.9) | 484.7 (252.7-886.2) | 5.9 (3.1-10.6) | 0.89 (0.77 to 1.01) |
| Samoa | 16.2 (11.7-21.6) | 14 (10.1-19.5) | 44.7 (31.3-62.7) | 25 (17.4-34.5) | 1.74 (1.61 to 1.86) |
| Solomon Islands | 8.1 (3.9-13) | 3.9 (1.8-6.4) | 41.9 (25-64.2) | 7.9 (4.7-12.3) | 2.15 (2.02 to 2.28) |
| Tokelau | 0.1 (0.1-0.1) | 6.7 (4.5-9.7) | 0.2 (0.2-0.3) | 16 (10.7-21.8) | 2.28 (2.08 to 2.47) |
| Tonga | 4.2 (3-5.7) | 6.2 (4.5-8.6) | 9.4 (5.8-13.7) | 10.6 (6.6-15.3) | 1.45 (1.26 to 1.64) |
| Tuvalu | 0.5 (0.3-0.6) | 5.6 (4.1-8) | 1.2 (0.8-1.7) | 10.4 (6.6-14.4) | 1.78 (1.58 to 1.99) |
| Vanuatu | 4.1 (2.7-6) | 4.3 (2.8-6.3) | 18.1 (12-24.9) | 7.4 (4.9-10.1) | 1.46 (1.35 to 1.58) |
| North Africa and Middle East | 31166 (25920.4-41990) | 13 (10.8-17.4) | 183491.2 (151632.4-216130.2) | 30.7 (25.4-36) | 3.16 (2.99 to 3.33) |
| Afghanistan | 715.1 (233.4-2022.6) | 9.6 (3.1-27.2) | 4245.9 (1868.7-7131.8) | 23.5 (10.2-39.9) | 3.31 (3.08 to 3.55) |
| Algeria | 2480 (1887.1-3208.2) | 13.8 (10.5-17.8) | 13921.1 (9564.7-18509.2) | 31 (21.5-41.1) | 2.95 (2.79 to 3.11) |
| Bahrain | 91.4 (70.8-125.6) | 29.3 (22.9-38.9) | 705.1 (520.9-958.6) | 46.4 (33.7-61.8) | 1.67 (1.53 to 1.8) |
| Egypt | 3565 (2945.6-4765.1) | 8.9 (7.3-12) | 17204.7 (12717.1-22821.4) | 19.5 (14.5-25.7) | 2.54 (2.41 to 2.67) |
| Iran (Islamic Republic of) | 3101.2 (2026.6-4032.1) | 8.2 (5.1-10.8) | 28838.3 (12164.9-36157.3) | 30.1 (12.9-37.7) | 5.33 (4.83 to 5.82) |
| Iraq | 2151.1 (1545.6-3209.1) | 18.4 (13.1-28) | 13392.3 (9601.2-18568.5) | 38.1 (27.4-52.3) | 2.85 (2.58 to 3.13) |
| Jordan | 539.8 (404.7-712.7) | 23.9 (18-32) | 3968.6 (2868.8-5553.6) | 36.3 (26.4-50.6) | 1.65 (1.29 to 2.01) |
| Kuwait | 478.8 (413.4-549.2) | 35.6 (31.1-40.6) | 2461.1 (1960.9-2930.3) | 46.5 (37.3-55.1) | 1.37 (0.39 to 2.35) |
| Lebanon | 615.6 (435.8-830.3) | 23.9 (17-32.2) | 2235.5 (1692.9-2995.8) | 37.2 (28-49.9) | 1.7 (1.54 to 1.86) |
| Libya | 746.8 (546.6-996.6) | 26.6 (19.2-36.5) | 3929.3 (2675.6-5582.5) | 51.1 (35.5-70.9) | 2.63 (2.27 to 2.98) |
| Morocco | 1714.8 (1300-2282.2) | 8.9 (6.7-11.8) | 7010 (4684.5-10279) | 17.9 (12-26.1) | 2.32 (2.26 to 2.38) |
| Palestine | 258.7 (172.7-358.5) | 23.4 (15.3-32.7) | 1191.4 (776-1507.9) | 32.8 (20.7-41.9) | 1.18 (1.05 to 1.32) |
| Oman | 122.8 (84.5-168.9) | 10 (7-13.3) | 911.1 (606.8-1227.3) | 22.2 (15.2-29.3) | 3.09 (2.76 to 3.41) |
| Qatar | 86.3 (63.4-116) | 30.1 (22.6-38.4) | 1210.8 (852.4-1675.4) | 49.1 (35.4-66.5) | 1.99 (1.62 to 2.37) |
| Saudi Arabia | 1739.8 (1132.4-2809.4) | 16 (10.6-27.2) | 26111.8 (18459.2-34798.9) | 60.1 (44.7-79.4) | 5.02 (4.73 to 5.32) |
| Sudan | 924.4 (354-2422.6) | 7 (2.7-17.9) | 5357.8 (3049-8629.8) | 16.9 (9.8-26.7) | 3.09 (2.92 to 3.27) |
| Syrian Arab Republic | 642.7 (335.8-1563.6) | 8.3 (4.3-19.8) | 3832.4 (2348.4-6090.9) | 25.3 (15.4-40.3) | 3.8 (3.62 to 3.97) |
| Tunisia | 1082.7 (805.1-1390.7) | 16.5 (12.2-21.3) | 4529.3 (3031-6120.6) | 33 (22-44.9) | 2.28 (2.16 to 2.4) |
| Turkey | 9398.8 (6785.5-12960.2) | 20.8 (15.2-28.3) | 36202.8 (26994.5-49450.2) | 37.1 (27.8-51) | 2.05 (1.87 to 2.23) |
| United Arab Emirates | 324.8 (214.5-471.6) | 33.1 (21.2-47.1) | 3275.3 (2486.8-4485.5) | 39.7 (30.8-51.4) | 1.49 (1.17 to 1.82) |
| Yemen | 368.4 (190.1-823.3) | 5.1 (2.6-11.4) | 2785.4 (1868.8-4013.6) | 12.6 (8.3-18.7) | 3.46 (3.17 to 3.76) |
| South Asia | 53580.2 (43818.6-70783.2) | 5.9 (4.9-7.8) | 282509.1 (227052.9-343508.3) | 15.3 (12.3-18.5) | 3.24 (3.16 to 3.33) |
| Bangladesh | 4626.7 (3192.4-6735.6) | 5.8 (4.1-8.4) | 23295.9 (14513.7-43137.2) | 14.1 (8.8-26) | 3.28 (3.12 to 3.44) |
| Bhutan | 26.1 (15.6-37.1) | 5.7 (3.5-8.1) | 104.5 (61.7-187.1) | 13.5 (8.1-23.3) | 2.66 (2.59 to 2.74) |
| India | 40550.3 (32766.5-54345.6) | 5.6 (4.6-7.5) | 214072.2 (170093.3-259618.3) | 14.8 (11.8-18) | 3.38 (3.26 to 3.5) |
| Nepal | 802.3 (515.1-1228.4) | 5.3 (3.5-8) | 4027.1 (2617.4-6430.6) | 13.7 (9-21.6) | 3.3 (3.16 to 3.43) |
| Pakistan | 7574.8 (5775.8-10285.6) | 9.1 (7.1-12.1) | 41009.4 (27812.5-58771.6) | 19.5 (13.4-27.5) | 2.19 (2.04 to 2.34) |
| Southern Sub-Saharan Africa | 2655.2 (2243.8-3153) | 6.9 (5.8-8.2) | 8000.6 (6622.7-9541.6) | 10.7 (8.9-12.7) | 1.8 (1.54 to 2.05) |
| Botswana | 39.5 (23.1-64.9) | 4.8 (2.8-7.9) | 137.5 (83.3-235.9) | 6.4 (3.9-10.9) | 1.54 (0.91 to 2.17) |
| Lesotho | 36.2 (23.3-59.9) | 3.4 (2.2-5.7) | 99.2 (64.1-158.7) | 7 (4.5-11) | 3.04 (2.68 to 3.41) |
| Namibia | 38.5 (27.5-51) | 4 (3-5.2) | 182.9 (115.3-273.2) | 9 (5.8-13.3) | 2.71 (2.62 to 2.8) |
| South Africa | 1844.3 (1577.6-2245.2) | 6.3 (5.3-7.6) | 5117.2 (4450.5-6498.8) | 9 (7.8-11.3) | 1.46 (1.17 to 1.75) |
| Eswatini | 26 (18.3-38.8) | 5.6 (4-8.4) | 82 (46.9-131.5) | 9.7 (5.6-15.2) | 1.82 (1.62 to 2.02) |
| Zimbabwe | 670.7 (462.6-875.5) | 10.8 (7.9-14.2) | 2381.7 (1428.5-3382.2) | 20.7 (12.9-29.1) | 2.68 (2 to 3.37) |
| Western Sub-Saharan Africa | 1674 (1212.8-2106) | 1.2 (0.9-1.5) | 6146.3 (4496.6-8323.4) | 1.8 (1.3-2.3) | 1.09 (1 to 1.18) |
| Benin | 45.1 (25.2-68.6) | 1.5 (0.8-2.3) | 156.2 (94.4-247.1) | 1.7 (1-2.6) | -0.03 (-0.18 to 0.13) |
| Burkina Faso | 114.8 (57.9-172.5) | 1.9 (0.9-2.8) | 291.8 (171.4-418.5) | 1.9 (1.1-2.7) | -0.18 (-0.31 to -0.05) |
| Cameroon | 158.2 (84.8-219.5) | 2.3 (1.2-3.1) | 577.3 (353-868.5) | 2.5 (1.5-3.8) | -0.07 (-0.28 to 0.14) |
| Cabo Verde | 1.6 (1.1-2.4) | 0.7 (0.5-1.1) | 26.7 (5.2-51.9) | 4.6 (0.9-8.9) | 6.05 (5.07 to 7.04) |
| Chad | 48.7 (26.4-74.8) | 1.3 (0.7-1.9) | 161.7 (105.8-229.9) | 1.5 (1-2.1) | 0.36 (0.26 to 0.45) |
| Cmte d'Ivoire | 294.6 (203.8-410.8) | 3.6 (2.6-4.8) | 1304 (859.1-1961.9) | 6 (4-8.7) | 1.99 (1.81 to 2.17) |
| Gambia | 12 (7.8-16.9) | 1.9 (1.3-2.6) | 58.9 (37.2-85.4) | 3.4 (2.2-4.9) | 1.72 (1.47 to 1.97) |
| Ghana | 41.7 (22.9-62) | 0.4 (0.2-0.5) | 176.5 (108.6-270.7) | 0.6 (0.4-0.9) | 1.5 (1.39 to 1.6) |
| Guinea | 110.4 (82.1-147.3) | 2.5 (1.9-3.3) | 358.6 (241.2-550.3) | 3.8 (2.6-5.8) | 1.23 (1.18 to 1.27) |
| Guinea-Bissau | 13.5 (7.4-19.1) | 2.1 (1.1-2.9) | 33.6 (22.6-48.3) | 2.3 (1.5-3.3) | 0.11 (0.02 to 0.2) |
| Liberia | 25.6 (13.2-37) | 1.5 (0.8-2.2) | 100 (61.5-145.6) | 2.4 (1.5-3.4) | 1.33 (1.2 to 1.45) |
| Mali | 369.1 (283-478.3) | 6.1 (4.8-7.7) | 1216.2 (780.7-1906.5) | 7.8 (5.2-12) | 0.77 (0.7 to 0.83) |
| Mauritania | 23.6 (12.8-31.9) | 1.7 (0.9-2.4) | 79.6 (52-121.6) | 2.6 (1.7-3.9) | 0.77 (0.58 to 0.96) |
| Niger | 63.2 (33.6-92.2) | 1.3 (0.6-1.8) | 187 (111.6-290) | 1.3 (0.8-2) | -0.18 (-0.27 to -0.08) |
| Nigeria | 181.2 (114.1-306.7) | 0.3 (0.2-0.5) | 905.7 (546.2-1581.7) | 0.6 (0.3-1) | 2.52 (2.33 to 2.71) |
| Sao Tome and Principe | 0.9 (0.2-1.8) | 0.8 (0.3-1.4) | 3.1 (1.2-5.8) | 1.5 (0.7-2.7) | 2.22 (2.09 to 2.35) |
| Senegal | 86.6 (43.3-136.3) | 1.8 (0.9-2.8) | 243.9 (152.3-398.6) | 2.1 (1.3-3.4) | 0.16 (0.01 to 0.31) |
| Sierra Leone | 39.1 (19.1-59.5) | 1.3 (0.6-2.1) | 120 (68.4-184.7) | 1.8 (1-2.8) | 0.85 (0.77 to 0.94) |
| Togo | 44 (24.3-66.6) | 1.9 (1-2.9) | 145.5 (84.3-235.2) | 2.2 (1.3-3.5) | 0.16 (-0.03 to 0.35) |
| Eastern Sub-Saharan Africa | 12047 (9301.5-15328.6) | 9.4 (7.3-12) | 48137 (34449.7-73226.7) | 15.4 (11.2-23.1) | 1.47 (1.29 to 1.66) |
| Burundi | 313.9 (204.1-474.5) | 8.3 (5.5-12.4) | 845.9 (528.3-1385.8) | 8.9 (5.7-14.5) | 0.02 (-0.17 to 0.2) |
| Comoros | 25.4 (14.1-42.6) | 7.9 (4.7-12.7) | 87.6 (53.3-147.5) | 12.8 (7.8-21.3) | 1.34 (1.09 to 1.6) |
| Djibouti | 17.7 (10.8-29.9) | 6.2 (4-10) | 127 (71.1-235.9) | 11.2 (6.4-20.2) | 1.99 (1.85 to 2.13) |
| Eritrea | 151.4 (119.8-203) | 6.7 (5.3-8.9) | 585.8 (366.2-958.7) | 11.4 (7.3-18.6) | 1.78 (1.71 to 1.84) |
| Ethiopia | 5871.9 (4084.6-8350.2) | 17.1 (12.1-24.2) | 20449.3 (13268-35627.6) | 25.6 (16.7-44.1) | 1 (0.75 to 1.25) |
| Kenya | 358.7 (240.5-562.6) | 2.4 (1.6-3.9) | 1950.1 (1361.5-3125.6) | 5 (3.6-7.8) | 2.66 (2.45 to 2.88) |
| Madagascar | 588.9 (456.5-799.9) | 7 (5.5-9.4) | 2288.9 (1516.2-3245.6) | 10.4 (6.9-14.7) | 1.28 (1.12 to 1.43) |
| Malawi | 450.6 (320.8-631.9) | 6.5 (4.6-8.8) | 1762 (1028.1-3022.2) | 12.5 (7.6-19.9) | 2.37 (2.26 to 2.49) |
| Mozambique | 589.9 (377.5-1047.5) | 6.1 (4-10.5) | 2371.9 (1388.1-4578.6) | 11.3 (6.6-21) | 2.25 (2.09 to 2.41) |
| Rwanda | 492.6 (356.1-682.3) | 9.9 (7.1-13.6) | 1396.6 (912.3-2223) | 13.1 (8.7-20.5) | 0.62 (0.31 to 0.94) |
| Somalia | 298.1 (182.2-437.5) | 5.8 (3.7-8.5) | 1002.3 (610.8-1519.2) | 7.7 (4.8-11.4) | 0.97 (0.92 to 1.01) |
| South Sudan | 226.6 (141.7-353.1) | 5.7 (3.7-8.6) | 771.3 (486.3-1257.3) | 11.2 (7.2-18.1) | 2.2 (1.86 to 2.55) |
| United Republic of Tanzania | 1449.4 (1012.4-2183.2) | 8.3 (6-12.2) | 5664.7 (3671.8-8907.9) | 13.1 (8.6-20.1) | 1.49 (1.31 to 1.67) |
| Uganda | 771.8 (529.2-1111.1) | 7.2 (5-10.2) | 6079.4 (3959.5-8693.2) | 20.9 (14.2-29.1) | 3.33 (3.17 to 3.48) |
| Zambia | 431.4 (329.3-564.4) | 8.1 (6.3-10.6) | 2712.2 (1246.1-5757.1) | 19.2 (9.3-39) | 3.13 (2.53 to 3.73) |
| Central Sub-Saharan Africa | 870.5 (619-1301.5) | 2.6 (1.8-3.9) | 3432 (2194.6-5420.7) | 4 (2.5-6.3) | 1.42 (1.14 to 1.7) |
| Angola | 146.4 (99-221.4) | 2.3 (1.5-3.4) | 844.9 (495-1396.8) | 4.3 (2.6-7.2) | 2.33 (2.12 to 2.55) |
| Central African Republic | 45.8 (32.9-67.1) | 2.7 (1.9-3.8) | 101.4 (63.1-160.5) | 2.7 (1.7-4.3) | 0.06 (-0.03 to 0.15) |
| Congo | 54.1 (38.7-79.8) | 3.7 (2.7-5.5) | 261.4 (154.2-427.2) | 6.1 (3.6-9.8) | 1.56 (1.34 to 1.77) |
| Democratic Republic of the Congo | 587.5 (395.4-925.5) | 2.5 (1.7-4.1) | 2056.1 (1240.5-3447.1) | 3.6 (2.1-6.1) | 1.14 (0.79 to 1.5) |
| Equatorial Guinea | 7 (4.7-10.5) | 2.6 (1.7-3.9) | 65.3 (33.8-110.6) | 7.4 (3.8-12.4) | 3.97 (3.73 to 4.22) |
| Gabon | 29.6 (21.4-43.3) | 4.4 (3.2-6.5) | 102.9 (64.6-160.7) | 7.1 (4.5-11) | 1.27 (1.13 to 1.42) |

**Table S2: Incidence cases and ASIR of thyroid cancer in 1990 and 2021 and its trends, by 204 countries and territories.**

|  | 1990 | | 2021 | |  |
| --- | --- | --- | --- | --- | --- |
| location | Number | ASR | Number | ASR | EAPC_95%CI |
| High-income North America | 12130.3 (11695.2-12450) | 3.8 (3.7-3.9) | 28289.1 (26782.7-29536) | 5.3 (5.1-5.5) | 1.15 (0.97 to 1.33) |
| Canada | 932.9 (803.6-1075.7) | 3 (2.6-3.5) | 1891.1 (1549.4-2289.1) | 3.4 (2.8-4.2) | 0.21 (-0.05 to 0.47) |
| Greenland | 0.8 (0.6-1.1) | 1.8 (1.3-2.4) | 1.3 (0.9-1.8) | 1.8 (1.3-2.5) | 1.15 (0.58 to 1.72) |
| United States of America | 11196.3 (10784.5-11509) | 3.9 (3.8-4) | 26396.3 (25051.5-27472.2) | 5.5 (5.3-5.7) | 1.23 (1.05 to 1.42) |
| Australasia | 585.9 (522.4-660.1) | 2.6 (2.3-2.9) | 1949.1 (1569.7-2343.2) | 4.6 (3.7-5.5) | 2.61 (2.06 to 3.16) |
| Australia | 481 (419.2-550.5) | 2.6 (2.2-2.9) | 1725.4 (1383.8-2106.4) | 4.8 (3.9-6) | 2.88 (2.3 to 3.46) |
| New Zealand | 104.9 (88.9-124.7) | 2.8 (2.4-3.3) | 223.7 (185-262.8) | 3.2 (2.6-3.7) | 0.99 (0.53 to 1.46) |
| High-income Asia Pacific | 6950.1 (6496.3-7654.3) | 3.4 (3.2-3.8) | 14277.6 (12630.3-16476.5) | 4.4 (3.9-5.2) | 1.15 (0.68 to 1.61) |
| Brunei Darussalam | 4.7 (3.3-6.7) | 3.2 (2.4-4.5) | 16.9 (12.7-22.6) | 3.9 (2.9-5) | 1.11 (0.96 to 1.26) |
| Japan | 5941.2 (5619.3-6249.8) | 3.6 (3.4-3.8) | 10066.6 (9098.6-10909.7) | 4.3 (3.9-4.6) | 0.6 (0.36 to 0.86) |
| Singapore | 83.4 (71.9-96.4) | 3 (2.6-3.5) | 271.2 (225.3-334.6) | 3.2 (2.7-4) | 0.51 (0.05 to 0.97) |
| Republic of Korea | 920.7 (660-1592.8) | 2.5 (1.8-4.4) | 3922.9 (2980.1-5824) | 4.8 (3.6-7.1) | 2.85 (1.8 to 3.91) |
| Western Europe | 19206.5 (18291.7-20159.2) | 3.9 (3.7-4.1) | 26004.5 (23787.6-28201.5) | 3.8 (3.5-4.2) | 0.28 (-0.1 to 0.67) |
| Andorra | 1.7 (1.2-2.4) | 2.9 (2-4.1) | 4.2 (2.8-5.9) | 3.1 (2-4.3) | 0.55 (0.25 to 0.85) |
| Austria | 534.3 (476.9-596.7) | 5.3 (4.7-5.9) | 689.9 (569.6-825.6) | 4.8 (4-5.8) | -0.15 (-0.37 to 0.07) |
| Belgium | 434.4 (374.1-509.2) | 3.3 (2.8-3.8) | 590.2 (479.3-716.6) | 3.4 (2.8-4.2) | 0.36 (0.07 to 0.65) |
| Cyprus | 22.5 (17.1-28.5) | 2.9 (2.2-3.7) | 52.3 (39.2-72.5) | 2.8 (2.1-3.9) | -0.03 (-0.38 to 0.32) |
| Denmark | 119 (102.7-137.3) | 1.7 (1.5-2) | 150.4 (127-180.4) | 1.6 (1.3-1.9) | 0.15 (-0.38 to 0.69) |
| Finland | 215.9 (188.6-249.6) | 3.3 (2.9-3.9) | 270.3 (221.3-329.1) | 3 (2.4-3.7) | 0.21 (-0.44 to 0.87) |
| France | 3778.8 (3360.3-4227) | 5.4 (4.8-6.1) | 6261.3 (5175-7462.7) | 6.7 (5.5-8) | 1.09 (0.37 to 1.82) |
| Germany | 4224.9 (3716.2-4851.4) | 3.9 (3.4-4.5) | 4968.6 (4104.1-5935.7) | 3.7 (3-4.4) | 0.28 (-0.12 to 0.68) |
| Greece | 325 (296.1-355.2) | 2.4 (2.2-2.6) | 451.7 (399.3-508.7) | 2.7 (2.4-3) | 0.38 (0.17 to 0.59) |
| Iceland | 21.2 (18.4-24.3) | 8 (6.9-9.2) | 30.6 (25.2-37.5) | 6.5 (5.3-8.1) | -0.12 (-0.77 to 0.53) |
| Ireland | 104.4 (91-117.6) | 2.7 (2.4-3.1) | 188 (156.6-226.3) | 2.8 (2.3-3.3) | 1.03 (0.68 to 1.39) |
| Israel | 148.6 (127.7-175.5) | 3.2 (2.7-3.8) | 354.6 (292.5-431) | 3.3 (2.7-4) | 0.31 (-0.23 to 0.86) |
| Italy | 4878.2 (4510.6-5317.7) | 6.4 (5.9-7) | 5311.7 (4772.4-5869.8) | 5.3 (4.8-5.8) | -0.6 (-0.94 to -0.25) |
| Luxembourg | 21 (19.1-23) | 4.2 (3.8-4.6) | 32.6 (28.4-37.8) | 3.4 (3-4) | -0.39 (-0.7 to -0.07) |
| Malta | 12.7 (10.8-15) | 3 (2.6-3.6) | 22.7 (18.4-28) | 3.2 (2.6-4) | 0.45 (-0.03 to 0.94) |
| Monaco | 1.5 (1.1-1.9) | 3 (2.3-3.9) | 2.7 (2-3.5) | 4.3 (3.1-5.9) | 1.34 (1.19 to 1.49) |
| Netherlands | 322.2 (286.6-363) | 1.8 (1.6-2) | 586.5 (490-688.3) | 2.2 (1.8-2.6) | 1.31 (1.04 to 1.59) |
| Norway | 137.7 (127.5-148.4) | 2.4 (2.2-2.6) | 229 (200.8-255.2) | 2.7 (2.4-3) | 0.88 (0.38 to 1.38) |
| Portugal | 418.8 (359.5-482.1) | 3.3 (2.8-3.8) | 644.6 (513.1-800.2) | 3.6 (2.8-4.4) | 0.37 (0.01 to 0.73) |
| San Marino | 1.6 (1.3-2) | 5.2 (4.2-6.4) | 2.2 (1.3-3.3) | 4.2 (2.4-6.2) | 0.24 (-0.17 to 0.65) |
| Spain | 1265 (1136-1400.4) | 2.7 (2.4-3) | 1938.6 (1619.6-2301.6) | 2.5 (2.1-3) | 0.14 (-0.21 to 0.49) |
| Sweden | 272 (234.4-318.3) | 2.1 (1.9-2.5) | 328.9 (269.3-396.6) | 1.9 (1.6-2.4) | 0.37 (0.03 to 0.7) |
| Switzerland | 229.5 (204.5-257.2) | 2.5 (2.2-2.8) | 277.5 (231.2-328.1) | 1.9 (1.6-2.2) | -0.55 (-1.32 to 0.22) |
| United Kingdom | 1699.6 (1647.1-1749) | 2.2 (2.2-2.3) | 2592.7 (2464.1-2695.1) | 2.6 (2.4-2.7) | 1.02 (0.81 to 1.22) |
| Southern Latin America | 1000.1 (896.4-1118.9) | 2.1 (1.9-2.4) | 2046.9 (1787.2-2334) | 2.5 (2.2-2.9) | 0.7 (0.48 to 0.92) |
| Argentina | 659.1 (581.6-752.2) | 2 (1.8-2.3) | 1170.4 (964.1-1414.2) | 2.2 (1.8-2.7) | 0.41 (0.16 to 0.65) |
| Chile | 258.8 (214.9-305.1) | 2.3 (1.9-2.8) | 726.3 (620.2-868.8) | 3.1 (2.6-3.7) | 1.05 (0.81 to 1.29) |
| Uruguay | 82.1 (66.9-99.3) | 2.3 (1.9-2.8) | 150.1 (124.7-183.1) | 3.4 (2.8-4.1) | 1.3 (1.13 to 1.46) |
| Eastern Europe | 6467.8 (6164.2-6831) | 2.4 (2.3-2.6) | 9617 (8698.1-10650.3) | 3.2 (2.9-3.5) | 1.27 (0.8 to 1.75) |
| Belarus | 300.3 (249.7-362.8) | 2.5 (2-3) | 545.8 (426.8-698.2) | 3.8 (3-4.9) | 0.88 (0.38 to 1.38) |
| Estonia | 58.3 (45.2-73.6) | 3.1 (2.4-4) | 80 (60.7-102.5) | 3.8 (2.9-4.9) | 0.82 (0.27 to 1.37) |
| Latvia | 82.8 (64.2-105.8) | 2.6 (2-3.3) | 124.7 (94-161.1) | 4 (3-5.2) | 1 (0.66 to 1.34) |
| Lithuania | 131.1 (104.3-162.6) | 3.1 (2.5-3.9) | 166.4 (131.9-204.9) | 3.7 (2.9-4.6) | 0.28 (-0.22 to 0.79) |
| Republic of Moldova | 80 (71.5-89.4) | 1.8 (1.6-2) | 126.4 (106.4-150.9) | 2.3 (2-2.8) | 0.87 (0.65 to 1.1) |
| Russian Federation | 4393 (4249-4543.3) | 2.5 (2.4-2.6) | 6782.1 (6175.3-7461.3) | 3.3 (3-3.6) | 1.47 (0.93 to 2.01) |
| Ukraine | 1422.3 (1181.6-1751.9) | 2.2 (1.8-2.7) | 1791.7 (1234.7-2539.7) | 2.8 (1.9-4) | 0.7 (0.28 to 1.12) |
| Central Europe | 4654.9 (4428.6-4883.6) | 3.2 (3-3.4) | 4876.2 (4406.6-5323.5) | 2.7 (2.4-2.9) | -0.65 (-0.86 to -0.45) |
| Albania | 39.5 (28.4-52.7) | 1.6 (1.2-2.1) | 76.8 (53-110.9) | 2 (1.4-2.9) | 1.21 (1 to 1.41) |
| Bosnia and Herzegovina | 80.7 (58.2-97) | 1.8 (1.3-2.1) | 103.7 (76.1-139.7) | 1.9 (1.4-2.6) | 0.25 (0.14 to 0.37) |
| Bulgaria | 246.3 (208-292.2) | 2.1 (1.8-2.5) | 250.9 (195.4-317.7) | 2.2 (1.7-2.8) | 0.49 (0.15 to 0.82) |
| Croatia | 281.5 (243.5-326.5) | 4.6 (4-5.3) | 248.6 (201.7-302.3) | 3.5 (2.8-4.3) | -0.74 (-1.04 to -0.44) |
| Czechia | 558.9 (472.4-662.9) | 4.4 (3.7-5.2) | 639.5 (486.7-828.1) | 3.8 (2.9-5) | -0.37 (-0.71 to -0.02) |
| Hungary | 503.6 (436-574.3) | 3.8 (3.2-4.3) | 448.6 (365.1-535.3) | 2.9 (2.4-3.5) | -1.19 (-1.42 to -0.97) |
| Montenegro | 20.6 (16.3-26.7) | 3.2 (2.5-4.1) | 33.7 (26.3-42.7) | 3.8 (3-4.8) | 0.98 (0.86 to 1.1) |
| North Macedonia | 40.1 (29.9-47.1) | 2 (1.5-2.4) | 69.9 (52.8-89.2) | 2.2 (1.7-2.8) | 0.23 (0.04 to 0.42) |
| Poland | 1858.8 (1738.6-1974.8) | 4.4 (4.1-4.6) | 1631.7 (1450.1-1829.7) | 2.8 (2.5-3.1) | -1.59 (-2.04 to -1.13) |
| Romania | 531.8 (457.7-621) | 2 (1.7-2.3) | 674.7 (525.1-871.3) | 2.2 (1.7-2.9) | 0.02 (-0.26 to 0.3) |
| Serbia | 183 (137.8-246.5) | 1.6 (1.2-2.2) | 308.3 (219.7-424.5) | 2.2 (1.6-3) | 0.86 (0.7 to 1.02) |
| Slovakia | 161.7 (127.8-199.6) | 2.8 (2.2-3.5) | 234.6 (179.1-318.1) | 2.8 (2.2-3.9) | 0.06 (-0.03 to 0.16) |
| Slovenia | 74 (62.6-87.1) | 3.1 (2.6-3.6) | 84.3 (61.1-113) | 2.3 (1.7-3.1) | -0.51 (-0.88 to -0.13) |
| Central Asia | 914 (840.8-996.1) | 1.7 (1.6-1.9) | 1630.9 (1432.2-1845.3) | 1.7 (1.5-2) | 0.01 (-0.73 to 0.75) |
| Armenia | 49.9 (36.9-64.4) | 1.6 (1.2-2.1) | 145.3 (109.5-190.2) | 3.6 (2.7-4.7) | 3.09 (2.65 to 3.53) |
| Azerbaijan | 60.4 (44.2-84.2) | 1.1 (0.8-1.4) | 151.2 (104.6-212.4) | 1.3 (0.9-1.8) | 0.68 (0.44 to 0.91) |
| Georgia | 116.6 (92-146.3) | 1.9 (1.5-2.4) | 207.7 (168.6-257.8) | 4.1 (3.3-5.1) | 2.75 (1.54 to 3.96) |
| Kazakhstan | 509.2 (447.3-576.1) | 3.6 (3.1-4) | 589.5 (479.8-705.3) | 3 (2.4-3.5) | -0.86 (-1.63 to -0.08) |
| Kyrgyzstan | 80.2 (62.3-104.8) | 2.3 (1.8-3) | 148 (105.3-199.8) | 2.5 (1.8-3.3) | 0.58 (-0.38 to 1.55) |
| Mongolia | 17.2 (12.4-24.6) | 1.4 (1-2) | 56.7 (42.5-77.9) | 1.9 (1.4-2.6) | 0.91 (0.43 to 1.39) |
| Tajikistan | 0.6 (0.4-0.8) | 0 (0-0) | 1.1 (0.7-1.6) | 0 (0-0) | -1.23 (-1.42 to -1.03) |
| Turkmenistan | 40.4 (36.1-45.5) | 1.7 (1.5-1.9) | 86.4 (65.6-116.2) | 1.8 (1.4-2.4) | 0.04 (-0.94 to 1.02) |
| Uzbekistan | 39.5 (29.1-52.3) | 0.3 (0.2-0.4) | 245.1 (179.4-333.8) | 0.8 (0.6-1) | 3.24 (2.53 to 3.96) |
| Central Latin America | 1712.2 (1651.2-1775.4) | 1.7 (1.7-1.8) | 7752.6 (6907.4-8701.4) | 3 (2.6-3.3) | 1.66 (1.52 to 1.8) |
| Colombia | 516 (469.6-564.8) | 2.4 (2.2-2.6) | 2087 (1686.5-2599.2) | 3.8 (3.1-4.7) | 1.37 (1.06 to 1.68) |
| Costa Rica | 55 (49.3-61.6) | 2.6 (2.3-2.9) | 189 (159.1-220.4) | 3.5 (2.9-4) | 0.4 (0.07 to 0.74) |
| Guatemala | 59 (55.1-62.9) | 1.4 (1.3-1.5) | 264.7 (223.2-311.2) | 2.1 (1.8-2.5) | 0.88 (0.6 to 1.17) |
| Honduras | 13.2 (10.2-16.5) | 0.5 (0.4-0.7) | 70.1 (48.6-96.2) | 1 (0.7-1.3) | 1.97 (1.74 to 2.19) |
| Mexico | 809.2 (783.6-833.7) | 1.6 (1.6-1.7) | 3987.8 (3477.8-4536) | 3 (2.6-3.4) | 1.91 (1.81 to 2.02) |
| Nicaragua | 20.3 (17.2-25) | 1 (0.9-1.3) | 102.3 (76.3-129.7) | 1.8 (1.4-2.3) | 2.29 (2.08 to 2.49) |
| Panama | 38.1 (32.7-43.9) | 2 (1.8-2.3) | 126.9 (101.2-158.9) | 2.9 (2.3-3.6) | 0.84 (0.41 to 1.28) |
| El Salvador | 56.8 (44.5-63.7) | 1.7 (1.3-1.9) | 200.7 (155.7-252.9) | 3.3 (2.5-4.1) | 2.2 (2.1 to 2.3) |
| Venezuela (Bolivarian Republic of) | 144.6 (133.4-155.7) | 1.3 (1.2-1.3) | 724.2 (542.2-941.5) | 2.4 (1.8-3.1) | 1.98 (1.83 to 2.13) |
| Andean Latin America | 422.2 (354-495.4) | 1.8 (1.5-2.1) | 2424 (1907.2-3044.3) | 3.9 (3.1-4.8) | 2.6 (2.44 to 2.77) |
| Bolivia (Plurinational State of) | 94.7 (59.6-128.8) | 2.6 (1.6-3.5) | 380.3 (250.8-542.3) | 3.8 (2.5-5.4) | 1.26 (1.21 to 1.31) |
| Ecuador | 87.9 (79.7-96.4) | 1.4 (1.3-1.5) | 714.1 (549.7-900.4) | 4.2 (3.2-5.3) | 3.73 (3.1 to 4.37) |
| Peru | 239.6 (194.1-293.7) | 1.7 (1.4-2.1) | 1329.6 (928.4-1839.8) | 3.8 (2.6-5.2) | 2.53 (2.34 to 2.73) |
| Caribbean | 442.8 (410.8-479.3) | 1.6 (1.5-1.7) | 1244.9 (1085.7-1427.4) | 2.4 (2.1-2.7) | 1.5 (1.32 to 1.69) |
| Antigua and Barbuda | 1 (0.9-1.1) | 1.8 (1.7-2) | 3.4 (3.1-3.7) | 3.1 (2.8-3.4) | 2.01 (1.58 to 2.44) |
| Barbados | 4.9 (4.5-5.3) | 1.9 (1.7-2) | 12.1 (9.5-15.4) | 2.8 (2.2-3.5) | 1.53 (1.32 to 1.74) |
| Belize | 0.7 (0.7-0.8) | 0.7 (0.6-0.7) | 5.2 (4.5-5.9) | 1.4 (1.2-1.6) | 2.67 (2.46 to 2.88) |
| Bermuda | 1.9 (1.7-2.1) | 2.8 (2.6-3.2) | 4.7 (3.8-5.9) | 4.6 (3.7-5.7) | 1.51 (1.19 to 1.83) |
| Bahamas | 3.8 (3.4-4.3) | 2 (1.8-2.2) | 13.7 (10.7-17.5) | 3.1 (2.4-3.9) | 1.97 (1.69 to 2.24) |
| Cuba | 189 (172.8-208.3) | 1.8 (1.6-2) | 559 (465.7-661.5) | 3.3 (2.7-3.9) | 2.01 (1.67 to 2.35) |
| Dominica | 0.8 (0.6-0.9) | 1.3 (1.1-1.6) | 1.3 (1-1.7) | 1.7 (1.3-2.1) | 0.93 (0.81 to 1.06) |
| Dominican Republic | 47 (37.5-56.1) | 1 (0.8-1.2) | 172.9 (128.4-231.1) | 1.6 (1.2-2.1) | 1.76 (1.63 to 1.89) |
| Grenada | 1.5 (1.3-1.7) | 2.2 (1.9-2.6) | 3.4 (2.8-4) | 2.9 (2.4-3.4) | 1.27 (0.88 to 1.66) |
| Guyana | 4.9 (4.2-5.6) | 1.1 (0.9-1.2) | 12.9 (9.6-17.2) | 1.8 (1.3-2.4) | 1.92 (1.74 to 2.1) |
| Haiti | 51.1 (33-71.7) | 1.3 (0.9-1.9) | 130.8 (87.9-188.8) | 1.4 (1-2) | 0.43 (0.35 to 0.52) |
| Jamaica | 24.2 (21.6-27) | 1.4 (1.2-1.5) | 80.2 (58.5-108.1) | 2.6 (1.9-3.5) | 2.14 (1.78 to 2.5) |
| Puerto Rico | 72.6 (64.5-80.7) | 2 (1.8-2.3) | 133.7 (107.5-163.5) | 2.8 (2.2-3.5) | 1.5 (1.17 to 1.84) |
| Saint Kitts and Nevis | 0.5 (0.4-0.5) | 1.4 (1.2-1.5) | 1.3 (1-1.5) | 1.7 (1.4-2.1) | 0.93 (0.59 to 1.27) |
| Saint Lucia | 2.2 (2-2.3) | 2.3 (2.2-2.5) | 7.2 (5.8-8.8) | 3.1 (2.5-3.8) | 0.82 (0.38 to 1.27) |
| Saint Vincent and the Grenadines | 1.8 (1.7-2) | 2.5 (2.2-2.7) | 5.3 (4.6-6.2) | 3.9 (3.4-4.6) | 1.73 (1.3 to 2.16) |
| Suriname | 3.6 (2.8-4.3) | 1.2 (1-1.5) | 9.7 (7.1-12.3) | 1.5 (1.1-1.9) | 1.01 (0.8 to 1.22) |
| Trinidad and Tobago | 15.3 (14.3-16.5) | 1.7 (1.5-1.8) | 44.8 (33.6-58.5) | 2.5 (1.9-3.3) | 1.44 (1.16 to 1.71) |
| United States Virgin Islands | 1.1 (0.8-1.3) | 1.1 (0.8-1.3) | 1.3 (0.9-1.8) | 1.1 (0.8-1.5) | 0.39 (0.25 to 0.54) |
| Tropical Latin America | 1371.6 (1299.4-1443.4) | 1.3 (1.2-1.3) | 4491.1 (4197.7-4757.9) | 1.7 (1.6-1.8) | 0.76 (0.6 to 0.91) |
| Brazil | 1328.6 (1256-1401.9) | 1.3 (1.2-1.3) | 4323.9 (4024.6-4580.3) | 1.7 (1.6-1.8) | 0.73 (0.57 to 0.89) |
| Paraguay | 43 (35.7-53.8) | 1.7 (1.4-2.1) | 167.1 (123.1-218.6) | 2.6 (1.9-3.4) | 1.57 (1.5 to 1.64) |
| East Asia | 13203.4 (10809.5-15460.8) | 1.3 (1.1-1.5) | 50885.2 (41562-63161.9) | 2.5 (2.1-3.1) | 2.43 (2.26 to 2.6) |
| China | 12157.4 (9714.1-14406) | 1.2 (1-1.5) | 48104.6 (38694.8-60068.1) | 2.5 (2-3.1) | 2.47 (2.29 to 2.65) |
| Democratic People's Republic of Korea | 302.1 (210.7-413.9) | 1.6 (1.1-2.2) | 776.4 (531.8-1105) | 2.4 (1.6-3.4) | 1.44 (1.32 to 1.57) |
| Taiwan (Province of China) | 743.8 (675-828.3) | 3.8 (3.5-4.2) | 2004.2 (1726.5-2263.7) | 5.9 (5-6.7) | 1.98 (1.61 to 2.34) |
| Southeast Asia | 6440.6 (5205.8-7276.5) | 2 (1.7-2.3) | 26559 (20898.8-31183.5) | 3.6 (2.9-4.2) | 1.82 (1.74 to 1.9) |
| Cambodia | 105 (67.4-138.7) | 1.9 (1.2-2.5) | 544.3 (340.6-771.8) | 3.8 (2.4-5.3) | 2.17 (2.03 to 2.31) |
| Indonesia | 2003.6 (1395.2-2416.8) | 1.6 (1.1-1.9) | 7343.3 (4749.2-9783) | 2.6 (1.7-3.4) | 1.37 (1.27 to 1.48) |
| Lao People's Democratic Republic | 43.8 (24.8-62.6) | 1.8 (1.1-2.6) | 154.2 (98.7-212.8) | 2.7 (1.7-3.7) | 1.17 (1.1 to 1.24) |
| Malaysia | 288.1 (231-345.4) | 2.4 (2-2.9) | 1279.6 (1061.4-1548) | 4 (3.3-4.8) | 1.62 (1.52 to 1.73) |
| Maldives | 1.4 (0.7-1.9) | 1.2 (0.7-1.7) | 6.2 (4.6-8.4) | 1.3 (1-1.7) | 0.12 (-0.15 to 0.38) |
| Mauritius | 14.9 (13.7-16.1) | 1.8 (1.6-1.9) | 28.3 (24.8-31.4) | 1.6 (1.4-1.8) | 0.14 (-0.76 to 1.05) |
| Myanmar | 496.7 (314.5-659.7) | 1.8 (1.2-2.4) | 1399.6 (941.1-1860.4) | 2.6 (1.8-3.4) | 0.94 (0.83 to 1.05) |
| Philippines | 1123.3 (839.4-1383.6) | 2.8 (2.1-3.5) | 4113.5 (3321.4-5119.6) | 4.2 (3.4-5.2) | 1.2 (1.12 to 1.27) |
| Sri Lanka | 288.7 (226.7-344.5) | 2.2 (1.7-2.7) | 835.4 (507.6-1203.3) | 3.2 (2-4.6) | 1.08 (0.9 to 1.27) |
| Seychelles | 0.5 (0.4-0.6) | 0.9 (0.7-1.1) | 1.4 (1.2-1.8) | 1.1 (0.9-1.4) | 0.76 (0.44 to 1.07) |
| Thailand | 1035.6 (829.2-1408.7) | 2.3 (1.9-3.2) | 3801 (2727.4-5756.9) | 3.9 (2.8-5.9) | 1.14 (0.77 to 1.51) |
| Timor-Leste | 5 (3.1-7.1) | 1.3 (0.8-1.8) | 19.6 (12.5-28.3) | 2.1 (1.3-3) | 1.68 (1.48 to 1.89) |
| Viet Nam | 1024.8 (755.7-1388.6) | 2.2 (1.6-3) | 6995.6 (4791.2-9263.3) | 6.3 (4.4-8.3) | 4.1 (3.88 to 4.32) |
| Oceania | 44.6 (29.9-60.2) | 1.2 (0.8-1.6) | 131.1 (80.2-185.4) | 1.4 (0.9-1.9) | 0.33 (0.24 to 0.42) |
| American Samoa | 0.6 (0.5-0.8) | 2.4 (1.8-3.1) | 1.8 (1.3-2.4) | 3.6 (2.5-4.7) | 1.5 (1.18 to 1.83) |
| Cook Islands | 0.3 (0.2-0.3) | 1.7 (1.3-2.2) | 0.5 (0.4-0.7) | 2.2 (1.6-3.2) | 0.09 (-0.25 to 0.43) |
| Micronesia (Federated States of) | 0.6 (0.5-0.9) | 1.1 (0.8-1.5) | 1.4 (1-2) | 1.7 (1.1-2.3) | 1.26 (1.18 to 1.35) |
| Fiji | 15.9 (10-21.3) | 3.3 (2.1-4.4) | 33.5 (20.1-47.7) | 3.9 (2.4-5.5) | 0.31 (0.19 to 0.42) |
| Guam | 0.8 (0.7-1.4) | 0.9 (0.7-1.5) | 3.5 (2.7-4.1) | 1.8 (1.4-2.2) | 3.31 (2.3 to 4.33) |
| Kiribati | 0 (0-0) | 0 (0-0.1) | 0 (0-0.1) | 0.1 (0-0.1) | 0.27 (0.06 to 0.48) |
| Marshall Islands | 0.2 (0.2-0.2) | 0.9 (0.8-1.2) | 0.7 (0.4-1) | 1.5 (1-2.1) | 1.38 (1.22 to 1.54) |
| Nauru | 0.1 (0.1-0.1) | 1.4 (0.9-1.9) | 0.2 (0.1-0.3) | 2.3 (1.3-3.3) | 1.57 (1.51 to 1.62) |
| Niue | 0 (0-0) | 1.3 (0.9-1.7) | 0 (0-0.1) | 2.3 (1.5-3) | 1.45 (1.3 to 1.6) |
| Northern Mariana Islands | 0.3 (0.2-0.5) | 1.1 (0.8-1.5) | 1 (0.7-1.2) | 1.6 (1.3-2) | 1.63 (1.01 to 2.25) |
| Palau | 0.2 (0.1-0.2) | 1.6 (1.2-2.2) | 0.5 (0.3-0.6) | 2.1 (1.5-2.7) | 0.7 (0.6 to 0.8) |
| Papua New Guinea | 17.9 (10.2-28.2) | 0.8 (0.4-1.2) | 66.7 (35.6-118.6) | 1 (0.5-1.7) | 0.59 (0.49 to 0.69) |
| Samoa | 2.2 (1.7-3) | 2.1 (1.6-3.1) | 5.6 (4-7.8) | 3.3 (2.3-4.7) | 1.27 (1.16 to 1.38) |
| Solomon Islands | 1.3 (0.6-2) | 0.8 (0.4-1.1) | 5.7 (3.5-8.7) | 1.3 (0.8-1.9) | 1.59 (1.5 to 1.69) |
| Tokelau | 0 (0-0) | 1.2 (0.8-1.6) | 0 (0-0) | 2.2 (1.4-2.9) | 1.6 (1.45 to 1.76) |
| Tonga | 0.6 (0.4-0.8) | 1 (0.7-1.3) | 1.3 (0.8-1.9) | 1.5 (0.9-2.2) | 1.22 (1.04 to 1.4) |
| Tuvalu | 0.1 (0.1-0.1) | 1.1 (0.8-1.5) | 0.2 (0.1-0.2) | 1.6 (1-2.1) | 1.12 (0.97 to 1.27) |
| Vanuatu | 0.6 (0.4-0.9) | 0.8 (0.6-1.1) | 2.6 (1.7-3.5) | 1.2 (0.8-1.6) | 1.14 (1.05 to 1.22) |
| North Africa and Middle East | 3792 (3150.6-5143.1) | 1.7 (1.4-2.3) | 21222.4 (17602-24974.6) | 3.7 (3.1-4.3) | 2.89 (2.72 to 3.06) |
| Afghanistan | 98.3 (33.4-275.9) | 1.4 (0.5-3.8) | 509.3 (227.3-855.1) | 3.1 (1.4-5.2) | 3.07 (2.86 to 3.28) |
| Algeria | 293.6 (224.3-378.3) | 1.7 (1.3-2.2) | 1596.9 (1105.4-2117.5) | 3.6 (2.6-4.8) | 2.75 (2.59 to 2.92) |
| Bahrain | 10.9 (8.6-14.9) | 4 (3.2-5.3) | 80.3 (59.2-108.7) | 5.7 (4.1-7.5) | 1.3 (1.15 to 1.45) |
| Egypt | 446.6 (369.4-603.9) | 1.2 (1-1.7) | 2055 (1530.1-2710.1) | 2.5 (1.9-3.2) | 2.34 (2.21 to 2.47) |
| Iran (Islamic Republic of) | 359.5 (235.9-462.8) | 1 (0.6-1.3) | 3291 (1397.7-4115.7) | 3.5 (1.5-4.4) | 5.2 (4.7 to 5.7) |
| Iraq | 258.3 (185.6-386.9) | 2.3 (1.6-3.6) | 1544.8 (1111.4-2119.4) | 4.6 (3.3-6.2) | 2.68 (2.41 to 2.94) |
| Jordan | 63 (47.6-82.8) | 3 (2.3-4) | 451.3 (327.1-631) | 4.3 (3.1-5.9) | 1.44 (1.09 to 1.79) |
| Kuwait | 53.9 (46.7-61.7) | 4.2 (3.7-4.8) | 274.7 (219.4-326.9) | 5.4 (4.3-6.4) | 1.29 (0.32 to 2.27) |
| Lebanon | 73.4 (52.8-98.1) | 2.9 (2.1-3.9) | 260.9 (198-348) | 4.3 (3.3-5.8) | 1.55 (1.39 to 1.7) |
| Libya | 87 (64-115.9) | 3.2 (2.3-4.4) | 446.1 (306.9-629.7) | 5.9 (4.2-8.2) | 2.54 (2.19 to 2.9) |
| Morocco | 211.8 (162-277.2) | 1.1 (0.9-1.5) | 834.4 (564.4-1204.1) | 2.2 (1.5-3.1) | 2.12 (2.05 to 2.18) |
| Palestine | 32.2 (21.6-44.3) | 3.1 (2-4.2) | 139 (90-175.5) | 4 (2.5-5.1) | 1 (0.89 to 1.12) |
| Oman | 14.6 (10.1-19.8) | 1.3 (0.9-1.7) | 103.1 (68.7-137.9) | 2.7 (1.8-3.5) | 2.88 (2.57 to 3.19) |
| Qatar | 9.9 (7.3-13.3) | 4.1 (3.1-5.2) | 134.7 (94.9-185.8) | 6 (4.3-7.9) | 1.64 (1.28 to 2) |
| Saudi Arabia | 207.7 (136.3-334.8) | 2.1 (1.4-3.5) | 2942.6 (2097.7-3917.1) | 7.1 (5.4-9.3) | 4.67 (4.38 to 4.97) |
| Sudan | 117.1 (46-305.2) | 0.9 (0.4-2.4) | 625.8 (360.5-998.3) | 2.1 (1.2-3.2) | 2.79 (2.62 to 2.96) |
| Syrian Arab Republic | 76.7 (40.5-186) | 1 (0.6-2.5) | 445 (274.8-701.7) | 3 (1.8-4.8) | 3.62 (3.46 to 3.78) |
| Tunisia | 127.4 (95.5-163.1) | 2 (1.5-2.6) | 522.5 (351.4-707.5) | 3.8 (2.6-5.2) | 2.13 (2.02 to 2.25) |
| Turkey | 1162.4 (853.6-1593.6) | 2.7 (2-3.7) | 4240 (3188.9-5745.7) | 4.4 (3.3-6) | 1.72 (1.55 to 1.89) |
| United Arab Emirates | 38.2 (25.5-55.1) | 4.5 (2.9-6.3) | 371.7 (282.6-509.4) | 5.2 (4.1-6.7) | 1.68 (1.28 to 2.08) |
| Yemen | 47.4 (24.8-106.5) | 0.7 (0.4-1.6) | 333.5 (226.5-482.6) | 1.6 (1.1-2.4) | 3.13 (2.86 to 3.4) |
| South Asia | 7853.1 (6476.2-10284.5) | 1 (0.8-1.3) | 37335.6 (30262.7-44931.4) | 2.1 (1.7-2.6) | 2.54 (2.46 to 2.62) |
| Bangladesh | 683.6 (489.8-973) | 1 (0.8-1.4) | 2983.1 (1875-5392.6) | 1.9 (1.2-3.3) | 2.24 (2.12 to 2.36) |
| Bhutan | 3.8 (2.3-5.3) | 1 (0.7-1.5) | 13.8 (8.6-23.3) | 1.9 (1.2-3.1) | 1.81 (1.73 to 1.88) |
| India | 5855.6 (4761.3-7738.9) | 0.9 (0.8-1.2) | 28334.1 (22851.2-34107.1) | 2.1 (1.7-2.5) | 2.73 (2.62 to 2.85) |
| Nepal | 119.9 (80.7-177.8) | 0.9 (0.6-1.4) | 528.5 (351.4-822.5) | 1.9 (1.3-2.9) | 2.46 (2.29 to 2.63) |
| Pakistan | 1190.2 (947.9-1556.4) | 1.6 (1.3-2.1) | 5476 (3849.3-7662.2) | 3 (2.1-4) | 1.68 (1.52 to 1.85) |
| Southern Sub-Saharan Africa | 376.9 (317.8-447.9) | 1.1 (0.9-1.3) | 1116.8 (934.8-1315.9) | 1.6 (1.3-1.9) | 1.54 (1.3 to 1.79) |
| Botswana | 6.2 (3.9-9.9) | 0.9 (0.6-1.4) | 19.7 (12.3-32.4) | 1.1 (0.7-1.7) | 0.97 (0.55 to 1.4) |
| Lesotho | 6.5 (4.4-10.5) | 0.7 (0.5-1.1) | 16.4 (11-25.5) | 1.3 (0.9-2) | 2.85 (2.42 to 3.28) |
| Namibia | 5.8 (4.5-7.6) | 0.7 (0.6-0.9) | 24.4 (15.9-35.6) | 1.3 (0.9-1.9) | 2.11 (1.99 to 2.22) |
| South Africa | 254.2 (214.3-304.2) | 1 (0.8-1.2) | 716.5 (614.7-880) | 1.4 (1.1-1.6) | 1.33 (1.16 to 1.5) |
| Eswatini | 4.1 (2.9-5.9) | 1.1 (0.8-1.6) | 12.1 (7.1-19) | 1.7 (1-2.5) | 1.53 (1.18 to 1.89) |
| Zimbabwe | 100 (73.7-128.4) | 1.9 (1.5-2.4) | 327.7 (205.9-456.7) | 3.3 (2.2-4.4) | 2.25 (1.58 to 2.93) |
| Western Sub-Saharan Africa | 254.3 (186.2-312.4) | 0.2 (0.2-0.3) | 803.8 (597.7-1068.9) | 0.3 (0.2-0.3) | 0.52 (0.45 to 0.58) |
| Benin | 7.2 (4-10.4) | 0.3 (0.2-0.4) | 20.5 (12.8-31.8) | 0.3 (0.2-0.4) | -0.69 (-0.85 to -0.53) |
| Burkina Faso | 18 (9.1-26) | 0.3 (0.2-0.5) | 38.7 (23.4-54.2) | 0.3 (0.2-0.4) | -0.83 (-0.99 to -0.67) |
| Cameroon | 23.6 (12.7-31.6) | 0.4 (0.2-0.5) | 74.3 (46.6-110.3) | 0.4 (0.2-0.5) | -0.63 (-0.77 to -0.48) |
| Cabo Verde | 0.2 (0.2-0.3) | 0.1 (0.1-0.2) | 3.2 (0.7-6.1) | 0.6 (0.1-1.1) | 5.65 (4.77 to 6.54) |
| Chad | 8.1 (4.5-12) | 0.2 (0.1-0.4) | 21.9 (14.7-30.5) | 0.2 (0.2-0.3) | -0.28 (-0.39 to -0.18) |
| Cmte d'Ivoire | 40.6 (29.2-54.9) | 0.6 (0.5-0.8) | 163.1 (109.9-238.6) | 0.9 (0.6-1.2) | 1.25 (1.13 to 1.36) |
| Gambia | 1.7 (1.1-2.3) | 0.3 (0.2-0.4) | 7.6 (5-10.8) | 0.5 (0.3-0.7) | 1.29 (1.07 to 1.51) |
| Ghana | 6.5 (3.5-9.5) | 0.1 (0-0.1) | 24.8 (15.8-36.3) | 0.1 (0.1-0.1) | 1.02 (0.9 to 1.13) |
| Guinea | 19.1 (14.8-24.8) | 0.5 (0.4-0.6) | 50.9 (35.1-75.3) | 0.6 (0.4-0.9) | 0.72 (0.69 to 0.76) |
| Guinea-Bissau | 2.1 (1.2-3) | 0.4 (0.2-0.5) | 4.4 (3-6.2) | 0.4 (0.2-0.5) | -0.53 (-0.64 to -0.43) |
| Liberia | 4.2 (2.2-5.9) | 0.3 (0.1-0.4) | 12.5 (7.9-18) | 0.3 (0.2-0.5) | 0.25 (0.11 to 0.39) |
| Mali | 55.8 (44.1-70.1) | 1.1 (0.9-1.3) | 160.6 (106.7-244) | 1.2 (0.8-1.7) | 0.28 (0.21 to 0.36) |
| Mauritania | 3.9 (2.1-5.2) | 0.3 (0.2-0.4) | 10.2 (6.9-15.3) | 0.4 (0.2-0.5) | -0.27 (-0.47 to -0.07) |
| Niger | 9.7 (5.1-13.6) | 0.2 (0.1-0.3) | 25.6 (15.9-38.8) | 0.2 (0.1-0.3) | -0.87 (-0.99 to -0.75) |
| Nigeria | 28.1 (17.9-49.6) | 0 (0-0.1) | 118.8 (73.3-206.9) | 0.1 (0.1-0.1) | 1.85 (1.69 to 2.02) |
| Sao Tome and Principe | 0.1 (0-0.2) | 0.1 (0.1-0.2) | 0.4 (0.2-0.7) | 0.2 (0.1-0.3) | 1.8 (1.69 to 1.91) |
| Senegal | 12.8 (6.5-19.5) | 0.3 (0.2-0.4) | 31.8 (20.5-50.7) | 0.3 (0.2-0.5) | -0.41 (-0.56 to -0.26) |
| Sierra Leone | 6.3 (3.1-9.3) | 0.2 (0.1-0.4) | 15.7 (9.3-23.5) | 0.3 (0.2-0.4) | 0.03 (-0.09 to 0.14) |
| Togo | 6.2 (3.4-9) | 0.3 (0.2-0.5) | 18.7 (11.2-29.6) | 0.3 (0.2-0.5) | -0.35 (-0.47 to -0.22) |
| Eastern Sub-Saharan Africa | 1908.4 (1516.6-2387.8) | 1.8 (1.5-2.2) | 6384.4 (4629.9-9478.7) | 2.4 (1.8-3.5) | 0.76 (0.6 to 0.92) |
| Burundi | 49.9 (34.2-72.9) | 1.6 (1.1-2.2) | 115.3 (74.2-184.9) | 1.5 (1-2.3) | -0.54 (-0.71 to -0.36) |
| Comoros | 3.7 (2.2-6) | 1.4 (0.9-2.1) | 12 (7.5-19.7) | 1.9 (1.2-3.2) | 1 (0.79 to 1.21) |
| Djibouti | 2.5 (1.6-4.1) | 1.1 (0.8-1.7) | 16.9 (9.8-30.3) | 1.8 (1.1-3) | 1.52 (1.43 to 1.61) |
| Eritrea | 23.4 (18.8-30.5) | 1.4 (1.1-1.7) | 81.3 (52.5-130.6) | 1.9 (1.3-2.9) | 1.26 (1.19 to 1.32) |
| Ethiopia | 970.8 (711.1-1320.7) | 3.5 (2.6-4.7) | 2701.3 (1775.1-4539.8) | 3.9 (2.6-6.4) | -0.01 (-0.26 to 0.24) |
| Kenya | 50.7 (34.2-78.8) | 0.4 (0.3-0.6) | 253.4 (179.5-397.4) | 0.7 (0.5-1.1) | 2.29 (2.1 to 2.48) |
| Madagascar | 87.1 (68.1-114.7) | 1.2 (1-1.6) | 296.5 (198.6-410.9) | 1.6 (1.1-2.2) | 0.85 (0.71 to 1) |
| Malawi | 66.3 (47.8-89.5) | 1.2 (0.9-1.5) | 230.5 (140.9-382) | 1.9 (1.2-2.9) | 1.73 (1.57 to 1.89) |
| Mozambique | 91.6 (60.8-151.5) | 1.1 (0.8-1.8) | 328.8 (197.9-607.9) | 1.9 (1.2-3.3) | 1.93 (1.8 to 2.07) |
| Rwanda | 75.8 (56.5-102.8) | 1.8 (1.4-2.5) | 186.7 (125.4-290.1) | 2 (1.4-3) | -0.23 (-0.51 to 0.05) |
| Somalia | 45.8 (29.1-64.2) | 1.2 (0.8-1.7) | 144.7 (90.8-213.6) | 1.4 (0.9-2.1) | 0.69 (0.65 to 0.74) |
| South Sudan | 36.6 (24.6-54.3) | 1.1 (0.8-1.5) | 105.6 (68.7-165.5) | 1.8 (1.2-2.7) | 1.66 (1.38 to 1.94) |
| United Republic of Tanzania | 215.1 (156.7-309.9) | 1.4 (1.1-2) | 749.4 (497.4-1149.9) | 2 (1.4-2.9) | 1.01 (0.88 to 1.14) |
| Uganda | 125.1 (89.9-172) | 1.4 (1.1-2) | 803.8 (535.8-1127.6) | 3.3 (2.3-4.5) | 2.59 (2.42 to 2.75) |
| Zambia | 62.6 (48.9-80.4) | 1.4 (1.1-1.8) | 352.6 (168.5-720.8) | 2.9 (1.5-5.5) | 2.52 (2.07 to 2.98) |
| Central Sub-Saharan Africa | 153.7 (111.5-225.9) | 0.6 (0.4-0.8) | 495.7 (319.2-770.7) | 0.7 (0.4-1.1) | 0.63 (0.42 to 0.83) |
| Angola | 25.4 (17.4-37.3) | 0.5 (0.4-0.8) | 118.4 (71-192.1) | 0.7 (0.4-1.2) | 1.28 (1.13 to 1.43) |
| Central African Republic | 8.7 (6.4-12.5) | 0.6 (0.5-0.9) | 16.8 (10.9-25.9) | 0.6 (0.4-0.9) | -0.29 (-0.34 to -0.24) |
| Congo | 9.7 (7-14.1) | 0.8 (0.6-1.1) | 36.2 (21.9-57.5) | 1 (0.6-1.5) | 0.67 (0.5 to 0.85) |
| Democratic Republic of the Congo | 103.3 (70.6-159.5) | 0.5 (0.4-0.9) | 301.6 (182.4-499) | 0.6 (0.4-1.1) | 0.47 (0.21 to 0.72) |
| Equatorial Guinea | 1.3 (0.9-1.9) | 0.6 (0.4-0.8) | 8.4 (4.5-14.1) | 1.1 (0.6-1.8) | 2.45 (2.25 to 2.64) |
| Gabon | 5.3 (3.9-7.5) | 0.9 (0.6-1.2) | 14.3 (9.3-21.9) | 1.1 (0.7-1.7) | 0.6 (0.49 to 0.71) |

**Table S3: Deaths cases and ASDR of thyroid cancer in 1990 and 2021 and its trends, by 204 countries and territories.**

|  | 1990 | | 2021 | |  |
| --- | --- | --- | --- | --- | --- |
| location | Number | ASR | Number | ASR | EAPC_95%CI |
| High-income North America | 1391.8 (1285-1450.1) | 0.4 (0.4-0.4) | 2765.7 (2473-2948.9) | 0.4 (0.4-0.4) | 0.15 (0.06 to 0.23) |
| Canada | 140.8 (123.3-159.1) | 0.4 (0.4-0.5) | 270.3 (224.8-324.1) | 0.4 (0.3-0.4) | -0.75 (-0.89 to -0.6) |
| Greenland | 0.2 (0.2-0.3) | 0.6 (0.5-0.8) | 0.3 (0.2-0.4) | 0.5 (0.3-0.6) | -0.39 (-0.85 to 0.07) |
| United States of America | 1250.8 (1149.9-1302.9) | 0.4 (0.4-0.4) | 2495.2 (2237.1-2640.7) | 0.4 (0.4-0.4) | 0.24 (0.16 to 0.33) |
| Australasia | 97.6 (87.2-109.1) | 0.4 (0.4-0.5) | 199.1 (161.3-237.4) | 0.4 (0.3-0.4) | 0.14 (-0.13 to 0.41) |
| Australia | 77.7 (68.1-88.2) | 0.4 (0.4-0.5) | 170.3 (135.8-206.5) | 0.4 (0.3-0.4) | 0.34 (0.09 to 0.6) |
| New Zealand | 19.9 (16.9-23.2) | 0.5 (0.4-0.6) | 28.8 (23.4-34.2) | 0.3 (0.3-0.4) | -0.78 (-1.17 to -0.4) |
| High-income Asia Pacific | 1235.9 (1126.1-1404.5) | 0.6 (0.6-0.7) | 2843.9 (2308.9-3185.8) | 0.5 (0.4-0.6) | -0.82 (-0.98 to -0.65) |
| Brunei Darussalam | 1.1 (0.8-1.4) | 1.2 (0.9-1.6) | 2.8 (2.2-3.6) | 1 (0.8-1.2) | -0.14 (-0.33 to 0.05) |
| Japan | 1034.3 (950.6-1080.5) | 0.6 (0.6-0.7) | 2292.3 (1815.7-2571.5) | 0.5 (0.4-0.5) | -0.87 (-0.96 to -0.77) |
| Singapore | 14.3 (12.2-16.5) | 0.7 (0.6-0.8) | 36.5 (29.9-44.3) | 0.4 (0.4-0.5) | -1.33 (-1.56 to -1.1) |
| Republic of Korea | 186.2 (138.1-339.5) | 0.7 (0.5-1.4) | 512.3 (407.1-689.2) | 0.6 (0.4-0.7) | -0.9 (-1.47 to -0.33) |
| Western Europe | 3951 (3667.4-4173.8) | 0.7 (0.6-0.7) | 3829.2 (3318.9-4190.8) | 0.4 (0.3-0.4) | -1.7 (-1.77 to -1.64) |
| Andorra | 0.3 (0.2-0.4) | 0.6 (0.4-0.8) | 0.6 (0.4-0.8) | 0.4 (0.3-0.5) | -1.18 (-1.34 to -1.02) |
| Austria | 111.9 (100.1-122.9) | 0.9 (0.8-1) | 98.4 (81.7-117.2) | 0.5 (0.4-0.6) | -1.92 (-2.04 to -1.8) |
| Belgium | 118.3 (100.8-137.9) | 0.8 (0.6-0.9) | 108 (87.3-131) | 0.4 (0.4-0.5) | -1.47 (-1.68 to -1.25) |
| Cyprus | 7.4 (5.8-9.2) | 1.1 (0.8-1.4) | 8.9 (6.8-11.4) | 0.4 (0.4-0.6) | -3.1 (-3.21 to -2.99) |
| Denmark | 33.9 (29.6-37.9) | 0.4 (0.4-0.5) | 35.2 (29.3-41.7) | 0.3 (0.2-0.3) | -1.12 (-1.47 to -0.78) |
| Finland | 51 (45.1-57.1) | 0.7 (0.6-0.8) | 48.3 (38.8-58.4) | 0.4 (0.3-0.4) | -1.86 (-2.22 to -1.5) |
| France | 570 (509-625.4) | 0.7 (0.6-0.7) | 527.6 (423.1-630.3) | 0.4 (0.3-0.4) | -2.04 (-2.43 to -1.66) |
| Germany | 1061.8 (939.8-1197.9) | 0.8 (0.7-0.9) | 860.8 (716.3-1013.9) | 0.4 (0.4-0.5) | -2.16 (-2.37 to -1.96) |
| Greece | 80.1 (73.9-86.3) | 0.5 (0.5-0.6) | 99.9 (86.8-110.5) | 0.4 (0.3-0.4) | -1.2 (-1.29 to -1.11) |
| Iceland | 4.2 (3.7-4.7) | 1.4 (1.3-1.6) | 4.4 (3.6-5.2) | 0.7 (0.6-0.9) | -1.53 (-2.06 to -0.99) |
| Ireland | 29.8 (26.3-33.6) | 0.7 (0.6-0.8) | 26.9 (21.8-32.2) | 0.3 (0.3-0.4) | -1.68 (-2.01 to -1.36) |
| Israel | 44.5 (38.2-52.1) | 0.9 (0.8-1.1) | 68.4 (56.8-81.9) | 0.5 (0.5-0.6) | -1.84 (-2.17 to -1.5) |
| Italy | 694.3 (638.3-739.6) | 0.8 (0.7-0.8) | 652.7 (556.7-723.1) | 0.4 (0.4-0.5) | -1.99 (-2.07 to -1.91) |
| Luxembourg | 5.8 (5.3-6.3) | 1.1 (1-1.2) | 5.6 (4.9-6.5) | 0.5 (0.4-0.6) | -2.19 (-2.29 to -2.1) |
| Malta | 3.5 (3-4) | 0.8 (0.7-1) | 4.2 (3.5-5.1) | 0.4 (0.3-0.5) | -2.06 (-2.4 to -1.71) |
| Monaco | 0.4 (0.3-0.5) | 0.6 (0.4-0.7) | 0.5 (0.4-0.6) | 0.5 (0.4-0.6) | -0.25 (-0.27 to -0.22) |
| Netherlands | 101.6 (90.8-113.2) | 0.5 (0.4-0.6) | 137.9 (113.7-157.6) | 0.4 (0.3-0.4) | -0.61 (-0.74 to -0.48) |
| Norway | 38.7 (35.2-41.5) | 0.5 (0.5-0.6) | 43.8 (37.4-48.6) | 0.4 (0.4-0.4) | -0.63 (-0.89 to -0.37) |
| Portugal | 101.2 (89.3-115.2) | 0.8 (0.7-0.9) | 97.2 (79-118.2) | 0.4 (0.3-0.5) | -2.07 (-2.22 to -1.93) |
| San Marino | 0.4 (0.3-0.5) | 1 (0.8-1.3) | 0.4 (0.3-0.6) | 0.5 (0.3-0.7) | -1.36 (-1.68 to -1.04) |
| Spain | 311.8 (281.9-341.3) | 0.6 (0.5-0.6) | 366.1 (298.8-440.9) | 0.3 (0.3-0.4) | -1.45 (-1.54 to -1.37) |
| Sweden | 90 (76.9-104.8) | 0.6 (0.5-0.7) | 92 (73.9-109.8) | 0.4 (0.3-0.5) | -0.92 (-1.15 to -0.7) |
| Switzerland | 60.4 (54.1-67.3) | 0.6 (0.5-0.6) | 60 (49.3-71.5) | 0.3 (0.3-0.4) | -1.9 (-2.33 to -1.48) |
| United Kingdom | 426.6 (402.5-440) | 0.5 (0.4-0.5) | 478.3 (430.1-504.1) | 0.4 (0.3-0.4) | -0.63 (-0.77 to -0.49) |
| Southern Latin America | 359 (324.7-394.6) | 0.8 (0.7-0.9) | 485.2 (425.8-553.7) | 0.6 (0.5-0.6) | -1.01 (-1.26 to -0.77) |
| Argentina | 247.9 (222.8-272.6) | 0.8 (0.7-0.9) | 305.7 (255.1-359.7) | 0.5 (0.5-0.6) | -0.98 (-1.25 to -0.7) |
| Chile | 81.9 (69.3-95) | 0.8 (0.7-1) | 139.6 (119.1-166) | 0.5 (0.5-0.6) | -1.34 (-1.57 to -1.1) |
| Uruguay | 29.2 (24.2-34.8) | 0.8 (0.6-0.9) | 39.9 (33.4-47.7) | 0.7 (0.6-0.8) | -0.22 (-0.31 to -0.13) |
| Eastern Europe | 1349.5 (1274-1423.5) | 0.5 (0.5-0.5) | 1650.5 (1508.2-1807.4) | 0.5 (0.4-0.5) | -0.25 (-0.6 to 0.09) |
| Belarus | 57.7 (47.3-69.9) | 0.5 (0.4-0.6) | 83.6 (65.8-106.5) | 0.5 (0.4-0.7) | 0.06 (-0.39 to 0.52) |
| Estonia | 11.7 (9.2-14.7) | 0.6 (0.5-0.7) | 15.5 (11.8-19.9) | 0.5 (0.4-0.7) | -0.42 (-0.79 to -0.05) |
| Latvia | 17.1 (13.7-21.4) | 0.5 (0.4-0.6) | 27.7 (21.3-34.9) | 0.7 (0.5-0.8) | 0.85 (0.47 to 1.23) |
| Lithuania | 19.6 (16.5-22.9) | 0.4 (0.4-0.5) | 30.8 (24.8-37.5) | 0.5 (0.4-0.6) | 0.33 (-0.11 to 0.77) |
| Republic of Moldova | 20.4 (18.2-22.8) | 0.5 (0.4-0.5) | 24.7 (20.8-29.3) | 0.4 (0.4-0.5) | -0.51 (-0.72 to -0.31) |
| Russian Federation | 920.6 (880.4-952.9) | 0.5 (0.5-0.5) | 1142.4 (1049.9-1243.6) | 0.5 (0.4-0.5) | -0.35 (-0.73 to 0.03) |
| Ukraine | 302.3 (248.7-373.2) | 0.4 (0.4-0.5) | 325.9 (236-438.1) | 0.4 (0.3-0.6) | -0.21 (-0.52 to 0.11) |
| Central Europe | 1266.4 (1213.2-1316.6) | 0.9 (0.8-0.9) | 985.7 (898-1064.6) | 0.4 (0.4-0.5) | -2.37 (-2.65 to -2.1) |
| Albania | 13.2 (9.7-16.4) | 0.7 (0.5-0.8) | 18.6 (13.4-25.8) | 0.4 (0.3-0.6) | -1.28 (-1.53 to -1.04) |
| Bosnia and Herzegovina | 22.5 (16.1-26.4) | 0.6 (0.4-0.7) | 23.9 (17.8-30.6) | 0.4 (0.3-0.5) | -1.72 (-2 to -1.44) |
| Bulgaria | 85.7 (73.2-99.6) | 0.7 (0.6-0.9) | 66.7 (53-84.6) | 0.5 (0.4-0.6) | -1.02 (-1.28 to -0.76) |
| Croatia | 51.4 (46.3-57.3) | 0.9 (0.8-1) | 37.5 (32.1-43.9) | 0.4 (0.3-0.5) | -2.48 (-2.8 to -2.16) |
| Czechia | 115.5 (98.5-135.9) | 0.8 (0.7-1) | 83 (66.3-102.1) | 0.4 (0.3-0.5) | -2.39 (-2.51 to -2.27) |
| Hungary | 136.9 (118.7-155.9) | 1 (0.8-1.1) | 81.9 (66.8-100.5) | 0.4 (0.3-0.5) | -2.71 (-2.97 to -2.45) |
| Montenegro | 3.6 (2.9-4.6) | 0.6 (0.5-0.8) | 6.1 (4.9-7.5) | 0.6 (0.5-0.8) | 0.22 (0.05 to 0.38) |
| North Macedonia | 12.2 (9.3-14.1) | 0.7 (0.5-0.8) | 15.6 (12.4-19.3) | 0.5 (0.4-0.6) | -1.25 (-1.39 to -1.11) |
| Poland | 517.3 (492.7-538) | 1.2 (1.1-1.2) | 347.6 (310.9-381.1) | 0.5 (0.4-0.5) | -3.27 (-3.81 to -2.74) |
| Romania | 164.9 (146.2-184.5) | 0.6 (0.5-0.7) | 151.5 (121.2-186.5) | 0.4 (0.3-0.5) | -1.76 (-2.06 to -1.47) |
| Serbia | 60.9 (46.9-79.8) | 0.6 (0.5-0.8) | 77.6 (57-102.4) | 0.5 (0.3-0.6) | -1.26 (-1.43 to -1.1) |
| Slovakia | 45 (36.9-54.2) | 0.8 (0.6-0.9) | 46.7 (37.3-60.4) | 0.5 (0.4-0.6) | -1.59 (-1.69 to -1.48) |
| Slovenia | 16.9 (14.3-19.6) | 0.7 (0.6-0.8) | 14.7 (10.7-19.1) | 0.3 (0.2-0.4) | -2.32 (-2.54 to -2.11) |
| Central Asia | 253.3 (235.5-274.3) | 0.5 (0.5-0.6) | 339.3 (301.7-378.5) | 0.4 (0.4-0.5) | -0.81 (-1.43 to -0.18) |
| Armenia | 13.2 (10.1-16.9) | 0.5 (0.4-0.6) | 34.9 (26.7-45) | 0.8 (0.6-1) | 1.81 (1.4 to 2.21) |
| Azerbaijan | 19.2 (14.5-25.4) | 0.4 (0.3-0.5) | 34 (25.1-43.7) | 0.3 (0.2-0.4) | -0.36 (-0.47 to -0.26) |
| Georgia | 30.9 (25.4-37.7) | 0.5 (0.4-0.6) | 53 (42.6-65.1) | 0.9 (0.7-1.1) | 2.4 (1.21 to 3.61) |
| Kazakhstan | 136.3 (121.5-153.5) | 1.1 (0.9-1.2) | 105.3 (86.6-125.1) | 0.6 (0.5-0.7) | -2.33 (-2.91 to -1.75) |
| Kyrgyzstan | 22.4 (17.5-28) | 0.8 (0.6-0.9) | 26.4 (20.2-33.7) | 0.5 (0.4-0.7) | -0.67 (-1.54 to 0.21) |
| Mongolia | 7.2 (5.4-9.9) | 0.7 (0.5-0.9) | 13 (10-17.1) | 0.6 (0.4-0.7) | -0.95 (-1.38 to -0.53) |
| Tajikistan | 0.2 (0.1-0.3) | 0 (0-0) | 0.3 (0.2-0.4) | 0 (0-0) | -1.72 (-1.9 to -1.54) |
| Turkmenistan | 12.8 (11.6-14.1) | 0.6 (0.6-0.7) | 19 (14.7-24.6) | 0.5 (0.4-0.6) | -1.3 (-2.26 to -0.33) |
| Uzbekistan | 11.1 (8.4-14.6) | 0.1 (0.1-0.1) | 53.5 (40-71.4) | 0.2 (0.2-0.3) | 2.55 (1.93 to 3.18) |
| Central Latin America | 635.6 (613.9-657.6) | 0.8 (0.8-0.8) | 1964.6 (1748.8-2165.4) | 0.8 (0.7-0.9) | -0.07 (-0.26 to 0.11) |
| Colombia | 178.2 (163.6-193.8) | 1 (0.9-1.1) | 467.5 (383.8-572.9) | 0.8 (0.7-1) | -0.88 (-1.22 to -0.54) |
| Costa Rica | 13.1 (11.9-14.3) | 0.7 (0.7-0.8) | 38.4 (32.9-44.3) | 0.7 (0.6-0.8) | -0.6 (-0.84 to -0.36) |
| Guatemala | 27.1 (25.7-28.5) | 0.8 (0.8-0.9) | 79.7 (68.7-91.7) | 0.7 (0.6-0.8) | -0.92 (-1.27 to -0.57) |
| Honduras | 5.7 (4.3-6.9) | 0.3 (0.2-0.3) | 25.3 (17.9-34.5) | 0.4 (0.3-0.6) | 1.33 (1.13 to 1.53) |
| Mexico | 318.3 (307-328.6) | 0.8 (0.8-0.8) | 1054.2 (936-1177.5) | 0.9 (0.8-1) | 0.25 (0.11 to 0.4) |
| Nicaragua | 7.2 (6-8.6) | 0.5 (0.4-0.6) | 25 (18.6-31.2) | 0.5 (0.4-0.7) | 0.65 (0.47 to 0.83) |
| Panama | 9.4 (8.4-10.4) | 0.6 (0.5-0.7) | 29.4 (23.2-36.1) | 0.7 (0.5-0.8) | 0.31 (0.14 to 0.49) |
| El Salvador | 24.1 (18.4-27) | 0.8 (0.6-0.9) | 49.3 (38.7-61) | 0.8 (0.6-1) | -0.1 (-0.29 to 0.09) |
| Venezuela (Bolivarian Republic of) | 52.6 (49-56) | 0.6 (0.5-0.6) | 195.7 (150.3-253.1) | 0.7 (0.5-0.9) | 0.38 (0.24 to 0.52) |
| Andean Latin America | 182.3 (153.4-211.5) | 0.9 (0.8-1) | 641.2 (507.9-794.5) | 1.1 (0.9-1.4) | 0.66 (0.54 to 0.77) |
| Bolivia (Plurinational State of) | 48.4 (30.7-66.2) | 1.5 (1-2.1) | 138.3 (90.2-191.9) | 1.6 (1-2.2) | 0.07 (0.03 to 0.11) |
| Ecuador | 35.2 (32.3-38.4) | 0.7 (0.6-0.7) | 204.6 (160.6-253.8) | 1.3 (1-1.6) | 2.36 (1.85 to 2.87) |
| Peru | 98.7 (79.7-119.3) | 0.8 (0.7-1) | 298.2 (205-400.6) | 0.9 (0.6-1.2) | 0.04 (-0.16 to 0.23) |
| Caribbean | 142.5 (131.7-156) | 0.6 (0.5-0.6) | 321.3 (280.7-367.5) | 0.6 (0.5-0.7) | 0.42 (0.2 to 0.65) |
| Antigua and Barbuda | 0.3 (0.3-0.4) | 0.6 (0.6-0.7) | 0.8 (0.7-0.8) | 0.8 (0.7-0.8) | 0.91 (0.45 to 1.38) |
| Barbados | 1.8 (1.7-1.9) | 0.6 (0.6-0.7) | 3.4 (2.7-4.2) | 0.7 (0.5-0.8) | 0.55 (0.35 to 0.74) |
| Belize | 0.3 (0.2-0.3) | 0.3 (0.2-0.3) | 1.2 (1-1.3) | 0.4 (0.3-0.4) | 1.33 (1.1 to 1.57) |
| Bermuda | 0.5 (0.5-0.6) | 0.9 (0.8-1) | 0.9 (0.7-1.1) | 0.7 (0.5-0.8) | -0.88 (-1.35 to -0.4) |
| Bahamas | 1.1 (1-1.2) | 0.7 (0.6-0.7) | 3 (2.4-3.7) | 0.8 (0.6-0.9) | 0.81 (0.48 to 1.15) |
| Cuba | 48.3 (44.8-52.1) | 0.5 (0.4-0.5) | 127.6 (107.2-148.5) | 0.6 (0.5-0.8) | 1.11 (0.72 to 1.49) |
| Dominica | 0.4 (0.3-0.5) | 0.6 (0.5-0.8) | 0.5 (0.4-0.6) | 0.6 (0.5-0.7) | 0.15 (0.05 to 0.25) |
| Dominican Republic | 17 (13.7-20) | 0.5 (0.4-0.5) | 48.3 (36.5-62.6) | 0.5 (0.4-0.6) | 0.51 (0.36 to 0.65) |
| Grenada | 0.6 (0.6-0.7) | 0.9 (0.8-1) | 0.9 (0.8-1.1) | 0.9 (0.7-1) | 0.55 (0.15 to 0.94) |
| Guyana | 2.2 (1.9-2.5) | 0.6 (0.5-0.6) | 4.3 (3.2-5.6) | 0.7 (0.5-0.9) | 0.86 (0.69 to 1.03) |
| Haiti | 27.3 (18.9-37.6) | 0.8 (0.6-1.2) | 52.8 (37.1-72.1) | 0.7 (0.5-1) | -0.28 (-0.35 to -0.22) |
| Jamaica | 8.7 (7.7-9.6) | 0.5 (0.4-0.5) | 20 (15.2-25.8) | 0.6 (0.5-0.8) | 1.01 (0.66 to 1.36) |
| Puerto Rico | 20.3 (18.6-22.1) | 0.6 (0.5-0.6) | 28.1 (23-33.5) | 0.4 (0.3-0.5) | -0.79 (-1.09 to -0.48) |
| Saint Kitts and Nevis | 0.2 (0.2-0.3) | 0.7 (0.6-0.7) | 0.4 (0.3-0.4) | 0.6 (0.5-0.7) | 0.02 (-0.33 to 0.38) |
| Saint Lucia | 0.8 (0.7-0.9) | 1 (0.9-1) | 1.9 (1.5-2.2) | 0.8 (0.7-0.9) | -1.04 (-1.61 to -0.47) |
| Saint Vincent and the Grenadines | 0.7 (0.7-0.8) | 1 (1-1.1) | 1.5 (1.3-1.7) | 1.1 (1-1.2) | 0.54 (0.05 to 1.03) |
| Suriname | 1.4 (1.1-1.6) | 0.6 (0.4-0.6) | 3 (2.2-3.9) | 0.5 (0.3-0.6) | -0.01 (-0.19 to 0.17) |
| Trinidad and Tobago | 5.5 (5.2-5.9) | 0.7 (0.6-0.7) | 11.5 (8.9-14.7) | 0.6 (0.5-0.8) | -0.47 (-0.72 to -0.21) |
| United States Virgin Islands | 0.3 (0.2-0.4) | 0.4 (0.3-0.5) | 0.4 (0.3-0.5) | 0.2 (0.2-0.3) | -1.42 (-1.59 to -1.26) |
| Tropical Latin America | 509 (480-537.2) | 0.6 (0.5-0.6) | 1252.8 (1142-1332) | 0.5 (0.5-0.5) | -0.6 (-0.69 to -0.5) |
| Brazil | 493.2 (464.2-521.3) | 0.6 (0.5-0.6) | 1206.3 (1100.6-1281.5) | 0.5 (0.4-0.5) | -0.63 (-0.73 to -0.53) |
| Paraguay | 15.8 (13.4-19.1) | 0.7 (0.6-0.9) | 46.5 (33.7-59.9) | 0.8 (0.6-1.1) | 0.57 (0.49 to 0.65) |
| East Asia | 3780.9 (3211.5-4380.1) | 0.5 (0.4-0.6) | 8063.8 (6456.3-9800.1) | 0.4 (0.3-0.5) | -0.65 (-0.74 to -0.55) |
| China | 3599.3 (3037.6-4182.3) | 0.5 (0.4-0.5) | 7692.2 (6122.5-9428.8) | 0.4 (0.3-0.5) | -0.66 (-0.76 to -0.57) |
| Democratic People's Republic of Korea | 76.9 (55.3-101.5) | 0.5 (0.4-0.7) | 149.1 (107.2-202.9) | 0.5 (0.3-0.6) | -0.18 (-0.27 to -0.08) |
| Taiwan (Province of China) | 104.7 (96.9-112.9) | 0.7 (0.6-0.7) | 222.4 (191-247.2) | 0.5 (0.5-0.6) | -0.39 (-0.57 to -0.2) |
| Southeast Asia | 2008.1 (1698.1-2303.1) | 0.8 (0.7-1) | 5642.9 (4564.6-6450.7) | 0.9 (0.7-1) | 0.32 (0.24 to 0.4) |
| Cambodia | 45.4 (31-58.7) | 1 (0.7-1.3) | 153.7 (98.6-209.9) | 1.3 (0.9-1.7) | 0.69 (0.56 to 0.83) |
| Indonesia | 681.6 (502-799.9) | 0.7 (0.5-0.8) | 1844.5 (1231.1-2421) | 0.8 (0.6-1.1) | 0.42 (0.29 to 0.55) |
| Lao People's Democratic Republic | 21.4 (12.9-30.3) | 1 (0.7-1.5) | 45.4 (29.8-62.3) | 1 (0.7-1.4) | -0.19 (-0.24 to -0.15) |
| Malaysia | 80 (67-98.1) | 0.9 (0.7-1.1) | 229.4 (195.1-287.4) | 0.8 (0.7-1.1) | -0.23 (-0.39 to -0.08) |
| Maldives | 0.5 (0.3-0.6) | 0.6 (0.4-0.8) | 1 (0.8-1.2) | 0.3 (0.2-0.4) | -2.23 (-2.36 to -2.09) |
| Mauritius | 4.2 (3.9-4.5) | 0.6 (0.6-0.7) | 6.3 (5.7-6.8) | 0.3 (0.3-0.4) | -1.21 (-2.03 to -0.38) |
| Myanmar | 205.3 (141.5-270.2) | 0.9 (0.6-1.2) | 402.3 (281.9-531.1) | 0.9 (0.6-1.1) | -0.38 (-0.49 to -0.26) |
| Philippines | 309.1 (235.5-381.8) | 1.1 (0.8-1.3) | 1018.8 (827.1-1226.9) | 1.3 (1.1-1.5) | 0.75 (0.71 to 0.79) |
| Sri Lanka | 85 (65.5-99.9) | 0.8 (0.7-1) | 152.1 (98-214.3) | 0.6 (0.4-0.8) | -1.43 (-1.63 to -1.23) |
| Seychelles | 0.2 (0.2-0.2) | 0.3 (0.3-0.4) | 0.3 (0.3-0.4) | 0.3 (0.2-0.3) | -0.51 (-0.8 to -0.22) |
| Thailand | 262.1 (214.8-356.6) | 0.8 (0.7-1.1) | 689.4 (512.3-966.7) | 0.6 (0.5-0.9) | -1.14 (-1.36 to -0.92) |
| Timor-Leste | 1.9 (1.2-2.7) | 0.7 (0.5-1) | 6.7 (4.3-9.6) | 0.8 (0.5-1.1) | 0.47 (0.34 to 0.61) |
| Viet Nam | 308.5 (229-464.5) | 0.8 (0.6-1.2) | 1085.1 (816.8-1422.2) | 1.2 (0.9-1.5) | 1.78 (1.59 to 1.98) |
| Oceania | 14.6 (10.3-19.3) | 0.6 (0.4-0.7) | 36.6 (23.3-50.4) | 0.5 (0.4-0.7) | -0.13 (-0.16 to -0.1) |
| American Samoa | 0.2 (0.1-0.2) | 1 (0.7-1.3) | 0.5 (0.3-0.6) | 1.1 (0.7-1.5) | 0.48 (0.23 to 0.74) |
| Cook Islands | 0.1 (0.1-0.1) | 0.7 (0.5-0.8) | 0.1 (0.1-0.2) | 0.5 (0.3-0.6) | -1.91 (-2.3 to -1.51) |
| Micronesia (Federated States of) | 0.3 (0.2-0.4) | 0.6 (0.5-0.8) | 0.4 (0.3-0.6) | 0.6 (0.4-0.9) | 0.13 (0.08 to 0.19) |
| Fiji | 4.7 (3.1-6.1) | 1.4 (0.9-1.8) | 9.4 (6-12.8) | 1.4 (0.9-1.8) | -0.23 (-0.36 to -0.09) |
| Guam | 0.2 (0.1-0.3) | 0.3 (0.3-0.5) | 0.7 (0.5-0.8) | 0.3 (0.2-0.4) | 0.87 (0.17 to 1.57) |
| Kiribati | 0 (0-0) | 0 (0-0.1) | 0 (0-0) | 0 (0-0.1) | -0.13 (-0.29 to 0.04) |
| Marshall Islands | 0.1 (0.1-0.1) | 0.5 (0.4-0.7) | 0.2 (0.1-0.3) | 0.6 (0.4-0.8) | 0.36 (0.2 to 0.52) |
| Nauru | 0 (0-0) | 0.6 (0.5-0.8) | 0 (0-0.1) | 0.8 (0.5-1.2) | 0.83 (0.67 to 1) |
| Niue | 0 (0-0) | 0.5 (0.4-0.7) | 0 (0-0) | 0.6 (0.4-0.8) | 0.22 (0.14 to 0.3) |
| Palau | 0.1 (0-0.1) | 0.7 (0.5-1) | 0.1 (0.1-0.2) | 0.7 (0.5-0.9) | 0.07 (0 to 0.14) |
| Papua New Guinea | 6.4 (3.9-9.4) | 0.4 (0.2-0.6) | 19.4 (10.8-32.1) | 0.4 (0.2-0.7) | 0.25 (0.19 to 0.32) |
| Samoa | 0.7 (0.5-1.1) | 0.8 (0.6-1.4) | 1.1 (0.8-1.7) | 0.8 (0.6-1.3) | -0.21 (-0.26 to -0.16) |
| Solomon Islands | 0.5 (0.3-0.8) | 0.4 (0.3-0.6) | 1.7 (1.1-2.5) | 0.5 (0.3-0.8) | 0.74 (0.68 to 0.8) |
| Tokelau | 0 (0-0) | 0.5 (0.4-0.7) | 0 (0-0) | 0.6 (0.4-0.7) | -0.03 (-0.12 to 0.06) |
| Tonga | 0.2 (0.1-0.3) | 0.4 (0.3-0.6) | 0.4 (0.2-0.5) | 0.5 (0.3-0.7) | 0.62 (0.45 to 0.8) |
| Tuvalu | 0 (0-0) | 0.6 (0.5-0.8) | 0.1 (0-0.1) | 0.6 (0.4-0.8) | -0.03 (-0.11 to 0.04) |
| Northern Mariana Islands | 0 (0-0.1) | 0.4 (0.3-0.5) | 0.2 (0.1-0.2) | 0.5 (0.3-0.5) | 1.21 (0.66 to 1.77) |
| Vanuatu | 0.2 (0.2-0.3) | 0.4 (0.3-0.6) | 0.8 (0.6-1.1) | 0.5 (0.4-0.7) | 0.64 (0.57 to 0.72) |
| North Africa and Middle East | 657.9 (546.1-936.5) | 0.4 (0.3-0.6) | 1935.2 (1676.1-2236.4) | 0.4 (0.4-0.5) | 0.64 (0.49 to 0.79) |
| Afghanistan | 31.2 (11.8-85.5) | 0.4 (0.2-1.2) | 80.6 (39-132.1) | 0.8 (0.4-1.2) | 2.06 (1.91 to 2.21) |
| Algeria | 41.7 (33.4-57.7) | 0.4 (0.3-0.5) | 136 (102.3-171.2) | 0.4 (0.3-0.5) | 0.9 (0.71 to 1.1) |
| Bahrain | 1.5 (1.2-1.8) | 1 (0.8-1.3) | 5.3 (3.8-7) | 0.8 (0.6-1) | -0.74 (-1 to -0.48) |
| Egypt | 93.7 (78.1-133.9) | 0.4 (0.3-0.6) | 252.6 (196.3-314.7) | 0.4 (0.4-0.6) | 1.01 (0.8 to 1.21) |
| Iran (Islamic Republic of) | 38.5 (27.2-46) | 0.2 (0.1-0.2) | 230 (104.2-278.6) | 0.3 (0.1-0.4) | 3.55 (2.94 to 4.16) |
| Iraq | 39.8 (29-61.2) | 0.5 (0.3-0.7) | 131.2 (95.8-170.8) | 0.6 (0.4-0.7) | 0.75 (0.56 to 0.94) |
| Jordan | 7.4 (5.8-9.8) | 0.6 (0.4-0.7) | 32.4 (24.3-43.6) | 0.5 (0.4-0.6) | -0.52 (-0.79 to -0.26) |
| Kuwait | 3.2 (2.8-3.5) | 0.5 (0.4-0.6) | 12.4 (10.2-14.9) | 0.5 (0.4-0.5) | 0.41 (-0.48 to 1.3) |
| Lebanon | 10.1 (7.7-13.3) | 0.5 (0.4-0.6) | 25.3 (20.2-32.1) | 0.4 (0.3-0.5) | -0.31 (-0.48 to -0.15) |
| Libya | 10.3 (7.7-14.4) | 0.5 (0.4-0.8) | 31.8 (23.9-42) | 0.6 (0.5-0.8) | 0.9 (0.72 to 1.08) |
| Morocco | 41.1 (32.9-50.4) | 0.3 (0.2-0.4) | 110.2 (82.7-140.4) | 0.3 (0.2-0.4) | 0.67 (0.59 to 0.74) |
| Palestine | 6.1 (3.9-8.2) | 0.7 (0.5-1) | 14.1 (8.7-17.4) | 0.6 (0.4-0.8) | -0.35 (-0.53 to -0.16) |
| Oman | 1.9 (1.3-2.4) | 0.3 (0.2-0.4) | 5.8 (4-7.4) | 0.3 (0.2-0.4) | 1.04 (0.78 to 1.31) |
| Qatar | 0.9 (0.7-1.1) | 1 (0.8-1.3) | 5.5 (3.9-7.5) | 0.7 (0.5-0.9) | -0.78 (-1.17 to -0.39) |
| Saudi Arabia | 31.9 (22.7-51.7) | 0.5 (0.4-0.9) | 144.7 (110.8-184.3) | 0.7 (0.6-0.9) | 1.23 (0.99 to 1.48) |
| Sudan | 25.6 (11.7-64) | 0.3 (0.1-0.7) | 71.7 (48.2-103.8) | 0.3 (0.2-0.5) | 1.23 (1.06 to 1.41) |
| Syrian Arab Republic | 11.6 (6.6-26.8) | 0.2 (0.1-0.5) | 42.9 (28.9-64.4) | 0.3 (0.2-0.5) | 1.63 (1.54 to 1.72) |
| Tunisia | 16.7 (13.1-21.7) | 0.3 (0.3-0.5) | 45.9 (32.3-64.2) | 0.4 (0.3-0.5) | 0.21 (0.12 to 0.29) |
| Turkey | 228.1 (182.2-290.2) | 0.7 (0.5-0.9) | 476.5 (376.3-602.9) | 0.5 (0.4-0.7) | -0.8 (-1 to -0.6) |
| United Arab Emirates | 5.1 (3.4-7.1) | 1.2 (0.8-1.7) | 28 (21.8-36.1) | 1.1 (0.9-1.4) | 1.77 (1.13 to 2.41) |
| Yemen | 11.2 (6.1-25.9) | 0.2 (0.1-0.6) | 50.3 (34.2-73.3) | 0.4 (0.2-0.5) | 1.73 (1.54 to 1.92) |
| South Asia | 2914.5 (2454.1-3700.4) | 0.5 (0.4-0.6) | 9323.8 (7794.2-10741.5) | 0.6 (0.5-0.7) | 0.92 (0.88 to 0.97) |
| Bangladesh | 264.2 (206.1-357.7) | 0.5 (0.4-0.7) | 683.8 (451.6-1075) | 0.5 (0.3-0.8) | -0.03 (-0.16 to 0.1) |
| Bhutan | 1.4 (1-2) | 0.5 (0.4-0.8) | 3.6 (2.5-5.3) | 0.6 (0.4-0.9) | 0.13 (0.07 to 0.19) |
| India | 2134.1 (1780.9-2785.2) | 0.4 (0.4-0.6) | 7156.2 (5975.9-8237.2) | 0.6 (0.5-0.7) | 1.17 (1.1 to 1.25) |
| Nepal | 47.5 (34-67.7) | 0.5 (0.3-0.7) | 137.3 (97.8-193.6) | 0.6 (0.4-0.8) | 0.81 (0.62 to 1) |
| Pakistan | 467.3 (381.9-594.4) | 0.8 (0.6-1) | 1342.9 (1014.7-1751.9) | 1 (0.8-1.3) | 0.63 (0.41 to 0.85) |
| Southern Sub-Saharan Africa | 123.6 (102.4-148.3) | 0.5 (0.4-0.6) | 323.8 (264.5-372.4) | 0.6 (0.5-0.7) | 0.92 (0.65 to 1.18) |
| Botswana | 2.6 (1.7-3.9) | 0.5 (0.3-0.7) | 6.3 (4.1-9.6) | 0.5 (0.3-0.7) | 0.05 (-0.22 to 0.33) |
| Lesotho | 3.4 (2.4-5.2) | 0.4 (0.3-0.7) | 7 (4.9-10.2) | 0.7 (0.5-1) | 2.38 (1.97 to 2.79) |
| Namibia | 2.3 (1.8-3) | 0.4 (0.3-0.5) | 6.5 (4.6-8.8) | 0.5 (0.3-0.6) | 0.97 (0.83 to 1.11) |
| South Africa | 77 (62.8-95.6) | 0.4 (0.3-0.5) | 200.9 (164.8-227.1) | 0.5 (0.4-0.5) | 0.73 (0.5 to 0.95) |
| Eswatini | 1.7 (1.3-2.3) | 0.6 (0.5-0.8) | 4 (2.6-6) | 0.7 (0.5-1.1) | 0.84 (0.48 to 1.21) |
| Zimbabwe | 36.6 (28.7-45.2) | 0.9 (0.8-1.2) | 99.1 (70.1-128.9) | 1.4 (1-1.8) | 1.83 (1.35 to 2.31) |
| Western Sub-Saharan Africa | 102.7 (77.8-122.8) | 0.1 (0.1-0.1) | 210.4 (165.3-263.6) | 0.1 (0.1-0.1) | -0.51 (-0.59 to -0.43) |
| Benin | 3.2 (1.8-4.4) | 0.1 (0.1-0.2) | 5.7 (3.9-8.1) | 0.1 (0.1-0.1) | -1.76 (-1.94 to -1.59) |
| Burkina Faso | 7.8 (4-10.7) | 0.2 (0.1-0.2) | 11.6 (7.8-15.6) | 0.1 (0.1-0.2) | -1.75 (-1.95 to -1.56) |
| Cameroon | 9.2 (5-12) | 0.2 (0.1-0.3) | 18.4 (12.6-26.4) | 0.1 (0.1-0.2) | -1.7 (-1.87 to -1.54) |
| Cabo Verde | 0.1 (0.1-0.1) | 0 (0-0) | 0.5 (0.1-1) | 0.1 (0-0.2) | 3.86 (3.3 to 4.41) |
| Chad | 3.9 (2.2-5.4) | 0.1 (0.1-0.2) | 7.1 (5.1-9.2) | 0.1 (0.1-0.1) | -1.15 (-1.34 to -0.95) |
| Gambia | 0.6 (0.4-0.7) | 0.2 (0.1-0.2) | 1.9 (1.4-2.5) | 0.2 (0.1-0.2) | 0.31 (0.14 to 0.48) |
| Ghana | 2.9 (1.5-4.1) | 0 (0-0.1) | 8.3 (5.1-11.4) | 0 (0-0.1) | 0.31 (0.22 to 0.4) |
| Guinea | 9.4 (7.5-11.8) | 0.3 (0.2-0.3) | 17.3 (12.5-23.5) | 0.3 (0.2-0.4) | 0.02 (-0.04 to 0.07) |
| Guinea-Bissau | 0.9 (0.5-1.3) | 0.2 (0.1-0.3) | 1.3 (0.9-1.7) | 0.1 (0.1-0.2) | -1.49 (-1.65 to -1.33) |
| Liberia | 2 (1-2.6) | 0.2 (0.1-0.2) | 2.8 (1.9-3.9) | 0.1 (0.1-0.2) | -1.52 (-1.67 to -1.37) |
| Mali | 22.1 (18-26.7) | 0.5 (0.4-0.6) | 44 (31.6-62.6) | 0.5 (0.3-0.6) | -0.54 (-0.62 to -0.45) |
| Mauritania | 1.8 (1-2.4) | 0.2 (0.1-0.2) | 2.4 (1.7-3.5) | 0.1 (0.1-0.2) | -2.13 (-2.31 to -1.96) |
| Niger | 4.2 (2.3-5.7) | 0.1 (0.1-0.2) | 8.3 (5.5-11.8) | 0.1 (0.1-0.1) | -1.86 (-2.04 to -1.69) |
| Nigeria | 11.1 (7.2-20.8) | 0 (0-0) | 27.7 (18.4-50.3) | 0 (0-0.1) | 0.52 (0.42 to 0.62) |
| Sao Tome and Principe | 0 (0-0.1) | 0 (0-0.1) | 0.1 (0-0.1) | 0.1 (0-0.1) | 0.45 (0.37 to 0.54) |
| Senegal | 5.1 (2.7-7.4) | 0.2 (0.1-0.2) | 8.8 (6.1-13.1) | 0.1 (0.1-0.2) | -1.46 (-1.6 to -1.32) |
| Sierra Leone | 2.9 (1.4-4) | 0.1 (0.1-0.2) | 4.2 (2.7-5.9) | 0.1 (0.1-0.1) | -1.26 (-1.44 to -1.09) |
| Togo | 2.1 (1.1-3) | 0.2 (0.1-0.2) | 4.7 (3-7) | 0.1 (0.1-0.2) | -1.34 (-1.48 to -1.2) |
| Cmte d'Ivoire | 13.3 (10-16.9) | 0.3 (0.2-0.4) | 35.4 (25.3-48.6) | 0.3 (0.2-0.4) | -0.15 (-0.24 to -0.05) |
| Eastern Sub-Saharan Africa | 838.7 (685.7-1015.1) | 1 (0.8-1.2) | 1800.9 (1337.6-2485.5) | 1 (0.7-1.3) | -0.18 (-0.29 to -0.07) |
| Burundi | 22.3 (16.1-30.8) | 0.9 (0.6-1.2) | 37.6 (24.6-55.8) | 0.7 (0.5-1) | -1.08 (-1.24 to -0.92) |
| Comoros | 1.5 (1-2.1) | 0.7 (0.5-0.9) | 3.8 (2.6-5.9) | 0.8 (0.5-1.1) | 0.28 (0.15 to 0.4) |
| Djibouti | 0.9 (0.6-1.3) | 0.6 (0.4-0.8) | 4.8 (3.1-7.7) | 0.7 (0.5-1.1) | 0.71 (0.65 to 0.77) |
| Eritrea | 9.8 (8.1-12.3) | 0.8 (0.6-1) | 26.8 (18.7-39) | 0.9 (0.7-1.3) | 0.63 (0.58 to 0.69) |
| Ethiopia | 459.7 (348-607.7) | 2.1 (1.6-2.8) | 753.6 (510.6-1130.3) | 1.6 (1.1-2.4) | -1.28 (-1.47 to -1.1) |
| Kenya | 16.7 (11.4-24.7) | 0.2 (0.1-0.3) | 65.9 (48.8-97.3) | 0.3 (0.2-0.4) | 1.83 (1.59 to 2.06) |
| Madagascar | 33.8 (26.9-42.5) | 0.6 (0.5-0.8) | 79.4 (54.9-107.3) | 0.6 (0.4-0.8) | 0.18 (0.04 to 0.31) |
| Malawi | 25.2 (19.1-32) | 0.6 (0.5-0.7) | 61.3 (40.6-89.2) | 0.8 (0.5-1) | 0.87 (0.68 to 1.07) |
| Mozambique | 40 (28.3-59.8) | 0.6 (0.5-0.9) | 106 (68.8-177.9) | 0.9 (0.6-1.4) | 1.44 (1.26 to 1.62) |
| Rwanda | 32.3 (24.8-42.9) | 1 (0.8-1.3) | 53.1 (37.6-75.4) | 0.8 (0.6-1.1) | -1.34 (-1.58 to -1.09) |
| Somalia | 19.4 (13-27) | 0.7 (0.5-1) | 54.8 (35.6-76.8) | 0.8 (0.5-1.1) | 0.49 (0.43 to 0.54) |
| South Sudan | 16.4 (11.7-22.7) | 0.6 (0.4-0.8) | 32.3 (22.3-46.7) | 0.8 (0.6-1.1) | 0.84 (0.64 to 1.04) |
| United Republic of Tanzania | 82.7 (61.9-111.3) | 0.7 (0.5-0.9) | 209 (148.5-289.6) | 0.8 (0.6-1) | 0.27 (0.18 to 0.36) |
| Uganda | 53.9 (40-72.9) | 0.8 (0.6-1.1) | 218.2 (155.2-292.1) | 1.3 (1-1.7) | 1.63 (1.45 to 1.82) |
| Zambia | 23.5 (19.1-29) | 0.7 (0.6-0.9) | 92.6 (46.8-166.7) | 1.1 (0.6-1.9) | 1.63 (1.34 to 1.93) |
| Central Sub-Saharan Africa | 78.4 (57.7-112.8) | 0.4 (0.3-0.5) | 182.8 (118.4-284) | 0.3 (0.2-0.6) | -0.11 (-0.23 to 0.01) |
| Angola | 12.9 (9-18.4) | 0.3 (0.2-0.5) | 40.6 (24.8-64.2) | 0.4 (0.2-0.6) | 0.26 (0.17 to 0.34) |
| Central African Republic | 4.8 (3.7-6.8) | 0.4 (0.3-0.6) | 8.1 (5.4-12) | 0.4 (0.3-0.6) | -0.5 (-0.54 to -0.45) |
| Congo | 4.9 (3.7-6.8) | 0.5 (0.4-0.7) | 11.4 (7.4-16.9) | 0.4 (0.3-0.6) | -0.46 (-0.58 to -0.35) |
| Democratic Republic of the Congo | 52.2 (36.3-79.1) | 0.3 (0.2-0.5) | 116.2 (70.2-189.6) | 0.3 (0.2-0.6) | -0.1 (-0.26 to 0.05) |
| Equatorial Guinea | 0.7 (0.5-1) | 0.4 (0.3-0.6) | 2 (1.2-3.2) | 0.4 (0.2-0.6) | 0.27 (0.1 to 0.43) |
| Gabon | 2.8 (2.1-3.8) | 0.5 (0.4-0.7) | 4.5 (3.1-6.4) | 0.5 (0.3-0.7) | -0.51 (-0.61 to -0.42) |

**TableS4: DALYs and age standardized DALYs rate of thyroid cancer in 1990 and 2021 and its trends, by 204 countries and territories.**

|  | 1990 | | 2021 | |  |
| --- | --- | --- | --- | --- | --- |
| location | Number | ASR | Number | ASR | EAPC_95%CI |
| High-income North America | 37316 (34968.1-39721.4) | 11.2 (10.5-12) | 70641.6 (65003.7-76456.6) | 12 (11-13) | 0.21 (0.11 to 0.31) |
| Canada | 3676.4 (3190.9-4193.4) | 11.6 (10-13.2) | 6155.9 (5054.3-7395) | 9.6 (7.8-11.6) | -0.81 (-1 to -0.63) |
| Greenland | 6.6 (4.7-8.7) | 16.5 (12.7-20.9) | 7.8 (5.3-10.5) | 11 (7.6-14.8) | -0.51 (-1 to -0.02) |
| United States of America | 33632.2 (31573.2-35773.7) | 11.2 (10.5-11.9) | 64476.8 (59384.2-69967.2) | 12.3 (11.3-13.4) | 0.31 (0.21 to 0.42) |
| Australasia | 2544 (2273.1-2867.8) | 11 (9.9-12.5) | 5041.1 (4103.8-6033.3) | 10.6 (8.7-12.8) | 0.51 (0.21 to 0.82) |
| Australia | 2037.1 (1784.6-2327.5) | 10.6 (9.3-12.1) | 4318 (3466.6-5259.4) | 10.8 (8.7-13.3) | 0.75 (0.45 to 1.06) |
| New Zealand | 507 (434.8-596.4) | 13.2 (11.3-15.5) | 723.1 (590.5-843.8) | 9.5 (7.8-11.1) | -0.62 (-0.99 to -0.24) |
| High-income Asia Pacific | 30855.9 (28505.4-35131.3) | 15.4 (14.1-17.5) | 50783.2 (43704.3-57571.4) | 11.8 (10.5-13.7) | -0.75 (-0.99 to -0.5) |
| Brunei Darussalam | 32.1 (23.9-42.8) | 27.9 (20.8-36.5) | 81.6 (63.7-103.9) | 22.3 (17.4-28.2) | -0.24 (-0.39 to -0.09) |
| Japan | 24888.6 (23406-26350.5) | 14.8 (13.9-15.7) | 38321.1 (32739.9-42214.3) | 11.5 (10.2-12.5) | -0.87 (-0.98 to -0.75) |
| Singapore | 406.3 (347.2-467.4) | 17.1 (14.6-19.8) | 848.5 (707.7-1036.3) | 10 (8.4-12.3) | -1.52 (-1.76 to -1.29) |
| Republic of Korea | 5528.8 (4125.5-9408.9) | 17.3 (12.9-30.6) | 11532 (9175-16858.4) | 13 (10.3-18.9) | -0.66 (-1.31 to -0.01) |
| Western Europe | 94707.5 (89027.2-100543.1) | 17.6 (16.6-18.8) | 84464.5 (75823.1-92550.5) | 10.5 (9.4-11.5) | -1.5 (-1.64 to -1.36) |
| Andorra | 8.6 (6.1-11.9) | 14.7 (10.4-20.2) | 14.1 (9.5-20.3) | 9.6 (6.5-13.9) | -1.03 (-1.2 to -0.87) |
| Austria | 2606.2 (2346.9-2869.2) | 23.6 (21.2-26.2) | 2260.2 (1874.7-2671.4) | 14 (11.5-16.6) | -1.68 (-1.75 to -1.61) |
| Belgium | 2721.7 (2344.7-3161.2) | 18.9 (16.3-21.9) | 2412.2 (1982.5-2910.4) | 12 (9.8-14.5) | -1.29 (-1.49 to -1.08) |
| Cyprus | 169.5 (132.8-211.7) | 22.3 (17.6-27.4) | 206.2 (156.6-275.9) | 10.5 (8-13.9) | -2.63 (-2.78 to -2.48) |
| Denmark | 799.2 (695.7-907.4) | 10.9 (9.5-12.3) | 737.9 (617.1-876) | 6.8 (5.6-8) | -1.33 (-1.71 to -0.95) |
| Finland | 1243 (1099.2-1408.2) | 18.3 (16.1-20.8) | 1042.4 (858.8-1255) | 9.7 (8-11.8) | -1.67 (-2.1 to -1.24) |
| France | 14236.2 (12841.3-15724.9) | 18.9 (17-20.9) | 12915.5 (10513.5-15389.7) | 11.4 (9.2-13.7) | -1.41 (-1.95 to -0.85) |
| Germany | 24311.6 (21483.3-27655.1) | 20.3 (18-23.1) | 18569.4 (15500-22064.7) | 11.2 (9.3-13.4) | -1.79 (-2.01 to -1.57) |
| Greece | 1855.2 (1714.7-2016.8) | 12.7 (11.7-13.8) | 2059.8 (1813.6-2305.6) | 10.1 (9-11.4) | -0.82 (-0.89 to -0.75) |
| Iceland | 103.4 (91.1-117) | 37.6 (33.1-42.5) | 102.6 (85.8-124.1) | 19.5 (16.2-23.7) | -1.54 (-2.05 to -1.03) |
| Ireland | 694.6 (616.3-778.8) | 17.4 (15.4-19.5) | 643.6 (533.3-772.8) | 8.7 (7.2-10.4) | -1.43 (-1.74 to -1.13) |
| Israel | 1066.9 (924.3-1254.5) | 22.3 (19.3-26.3) | 1538.4 (1274-1855.7) | 13.2 (10.9-15.9) | -1.72 (-2.08 to -1.36) |
| Italy | 18162.5 (16824.2-19562) | 22 (20.4-23.8) | 14512.6 (12904-16260) | 11.8 (10.6-13.3) | -1.95 (-2.07 to -1.84) |
| Luxembourg | 141.2 (128.7-153.8) | 27.1 (24.7-29.6) | 128.6 (112.7-146.4) | 12.7 (11.1-14.5) | -2.26 (-2.38 to -2.14) |
| Malta | 84 (72.3-97.8) | 19.7 (17-22.9) | 93.7 (77.4-113.4) | 11.2 (9.1-13.6) | -1.71 (-2.06 to -1.37) |
| Monaco | 8.6 (6.5-11) | 14.4 (11-18.4) | 10.9 (8.3-13.8) | 13.7 (10.1-17.8) | -0.09 (-0.13 to -0.05) |
| Netherlands | 2330.1 (2091.6-2598.1) | 12.1 (10.9-13.5) | 2942.8 (2455.1-3413.4) | 9.3 (7.7-10.9) | -0.51 (-0.66 to -0.36) |
| Norway | 829.5 (772.8-885.4) | 13.2 (12.3-14.1) | 896.9 (791-998.5) | 9.5 (8.5-10.6) | -0.73 (-1.04 to -0.42) |
| Portugal | 2430.5 (2140-2815.3) | 18.4 (16.2-21.1) | 2128.2 (1720.7-2630.8) | 10.1 (8-12.5) | -1.78 (-1.95 to -1.61) |
| San Marino | 8.4 (6.8-10.3) | 25.4 (20.4-31.1) | 8.8 (5.5-12.6) | 13.9 (8.4-20.1) | -1.07 (-1.39 to -0.76) |
| Spain | 7640.2 (6939.9-8375.3) | 15 (13.6-16.5) | 7707.2 (6510.4-9239.9) | 8.8 (7.5-10.5) | -1.52 (-1.66 to -1.38) |
| Sweden | 1852.9 (1598.9-2139.2) | 13.2 (11.4-15.2) | 1723.6 (1404.8-2081.2) | 8.7 (7.2-10.4) | -0.86 (-1.11 to -0.6) |
| Switzerland | 1403.3 (1262.3-1557.5) | 14.6 (13.2-16.2) | 1207.3 (1009.6-1423.8) | 7.2 (6.1-8.5) | -2.09 (-2.58 to -1.6) |
| United Kingdom | 9922.3 (9515.9-10280.7) | 12.1 (11.6-12.6) | 10527.2 (9827.8-11129.8) | 9.2 (8.7-9.7) | -0.51 (-0.66 to -0.35) |
| Southern Latin America | 9491.8 (8618.3-10469.6) | 20.3 (18.4-22.3) | 11959.8 (10465.3-13594.3) | 14.3 (12.5-16.2) | -1.02 (-1.28 to -0.75) |
| Argentina | 6508.9 (5852.1-7258.6) | 20 (18-22.3) | 7615.6 (6430.9-8934.5) | 14.1 (11.9-16.6) | -0.98 (-1.27 to -0.68) |
| Chile | 2261.5 (1916-2622.2) | 21.3 (18.1-24.6) | 3431.5 (2962.2-4038.9) | 13.9 (12-16.3) | -1.34 (-1.62 to -1.07) |
| Uruguay | 721 (601.1-864.1) | 19.4 (16-23.3) | 912.1 (764.1-1096.4) | 18.5 (15.5-22.3) | -0.14 (-0.23 to -0.04) |
| Eastern Europe | 37930 (35923.3-40329.4) | 13.9 (13.1-14.7) | 42085.6 (38218.3-46388.2) | 12.9 (11.7-14.1) | -0.29 (-0.68 to 0.11) |
| Belarus | 1630.6 (1361.1-1958.3) | 13.2 (11-15.7) | 2159.6 (1692.9-2770.7) | 14.2 (11.1-18.2) | -0.28 (-0.81 to 0.25) |
| Estonia | 311.2 (244-395.6) | 16 (12.6-20.3) | 331.1 (252.6-420.7) | 13.6 (10.3-17.3) | -0.72 (-1.13 to -0.3) |
| Latvia | 469.4 (370.7-592.8) | 14 (11-17.7) | 613.6 (470.9-780.6) | 17.3 (13.2-22.3) | 0.41 (0.02 to 0.8) |
| Lithuania | 569.4 (477.3-690.9) | 13.2 (11-16.1) | 719.9 (586.9-876.6) | 14.3 (11.6-17.3) | 0.01 (-0.48 to 0.49) |
| Republic of Moldova | 584.4 (523.5-648.5) | 13 (11.6-14.4) | 660.8 (557.1-783.3) | 11.5 (9.8-13.7) | -0.36 (-0.6 to -0.11) |
| Russian Federation | 25896.8 (24803.7-27028.6) | 14.6 (13.9-15.2) | 28639.2 (26154.1-31382.5) | 12.8 (11.7-14) | -0.37 (-0.8 to 0.07) |
| Ukraine | 8468.3 (7069.5-10307.6) | 12.3 (10.3-14.8) | 8961.5 (6370.3-12155.1) | 12.8 (9-17.2) | -0.14 (-0.49 to 0.2) |
| Central Europe | 34786.3 (33224.2-36474.4) | 23.5 (22.4-24.6) | 23433.7 (21250.4-25475.1) | 11.6 (10.5-12.7) | -2.47 (-2.76 to -2.17) |
| Albania | 385.7 (279.3-488.5) | 16.8 (12.1-21) | 444 (318.8-631.1) | 10.9 (7.7-15.4) | -1.29 (-1.5 to -1.09) |
| Bosnia and Herzegovina | 656.3 (470.3-775.8) | 15.1 (10.8-17.7) | 570.7 (428.2-738.7) | 9.7 (7.3-12.7) | -1.8 (-2.04 to -1.56) |
| Bulgaria | 2325.4 (1967.8-2734.3) | 19.6 (16.6-23.1) | 1633.4 (1291.7-2071) | 13.2 (10.4-16.7) | -0.93 (-1.21 to -0.66) |
| Croatia | 1410.2 (1255.9-1602.1) | 23.2 (20.6-26.2) | 877.5 (738.4-1041.3) | 10.9 (9.1-13) | -2.42 (-2.73 to -2.1) |
| Czechia | 2935.2 (2495.3-3470) | 22 (18.7-26) | 2020.5 (1579-2538.4) | 10.5 (8.1-13.3) | -2.19 (-2.34 to -2.04) |
| Hungary | 3699.7 (3246-4175.8) | 26.5 (23.3-29.8) | 2039.5 (1662.4-2490.7) | 11.8 (9.7-14.2) | -2.8 (-3.07 to -2.53) |
| Montenegro | 106 (85.2-134.6) | 16.5 (13.3-21) | 156.2 (123.3-195.2) | 16.7 (13.3-20.9) | 0 (-0.14 to 0.15) |
| North Macedonia | 345.1 (261.2-402) | 17.7 (13.5-20.6) | 397.2 (310.2-504.4) | 12.2 (9.6-15.4) | -1.49 (-1.61 to -1.36) |
| Poland | 14357.3 (13754.8-14938.7) | 33.1 (31.7-34.5) | 7938.9 (7126.5-8700) | 12 (10.8-13.2) | -3.52 (-4.09 to -2.94) |
| Romania | 4717.1 (4142.3-5396.5) | 17.1 (15-19.5) | 3695.6 (2905-4596.4) | 11.1 (8.6-13.8) | -1.91 (-2.23 to -1.6) |
| Serbia | 1639.5 (1254.6-2155.4) | 14.9 (11.5-19.7) | 1825.2 (1328.2-2426) | 11.9 (8.6-15.8) | -1.04 (-1.2 to -0.88) |
| Slovakia | 1205.4 (975.3-1456.9) | 20.5 (16.6-24.9) | 1167.6 (912.4-1529.2) | 13.1 (10.2-17.2) | -1.61 (-1.71 to -1.5) |
| Slovenia | 447.1 (378.1-521.5) | 18.5 (15.6-21.5) | 326.4 (239-428.1) | 8.1 (5.9-10.6) | -2.47 (-2.71 to -2.22) |
| Central Asia | 8144.5 (7568-8817.1) | 15.9 (14.7-17.2) | 10272.9 (9055.5-11543.5) | 11.7 (10.3-13) | -1.19 (-1.81 to -0.57) |
| Armenia | 396.9 (299.7-509.5) | 13.7 (10.4-17.5) | 891.9 (676.3-1144.2) | 21.2 (16-27.3) | 1.71 (1.29 to 2.13) |
| Azerbaijan | 611 (462.1-826.3) | 11 (8.3-14.7) | 1056.2 (768.1-1421) | 9.4 (6.9-12.4) | -0.57 (-0.68 to -0.46) |
| Georgia | 909.9 (749.5-1100.4) | 14.6 (12-17.7) | 1403.5 (1152-1700.4) | 25.8 (21.3-31.5) | 2.28 (1.09 to 3.48) |
| Kazakhstan | 4485.7 (4000.8-5041.7) | 32.3 (28.7-36.5) | 3221.8 (2644-3826.1) | 16.9 (13.9-20.1) | -2.69 (-3.27 to -2.1) |
| Kyrgyzstan | 705.7 (553-885) | 21.5 (16.9-26.8) | 866.4 (655.1-1130.7) | 15.7 (12-20.4) | -0.79 (-1.63 to 0.05) |
| Mongolia | 232.7 (173.2-328.5) | 19.1 (14.2-26.6) | 436.2 (333.8-576.1) | 15.9 (12.1-20.9) | -1.02 (-1.45 to -0.58) |
| Tajikistan | 5.8 (4-8) | 0.2 (0.1-0.3) | 8.8 (6-12) | 0.1 (0.1-0.2) | -1.69 (-1.83 to -1.56) |
| Turkmenistan | 432.6 (390.9-479.5) | 19.1 (17.3-21.1) | 637.7 (487.9-831.9) | 13.9 (10.7-17.9) | -1.23 (-2.17 to -0.27) |
| Uzbekistan | 364.1 (274.8-478.4) | 2.8 (2.1-3.7) | 1750.4 (1290.9-2371.1) | 5.8 (4.3-7.8) | 2.39 (1.75 to 3.03) |
| Central Latin America | 18293.1 (17666.1-18920.9) | 19.5 (18.9-20.2) | 52186.9 (47013.3-58083.4) | 20.4 (18.4-22.7) | 0 (-0.2 to 0.2) |
| Colombia | 5304.9 (4897.9-5758.5) | 26.5 (24.4-28.7) | 12032 (9855.6-14969.1) | 21.9 (17.9-27.2) | -0.86 (-1.22 to -0.49) |
| Costa Rica | 374.8 (342.2-410.3) | 19.3 (17.6-21.1) | 995.9 (852.1-1159.6) | 18.1 (15.5-21.1) | -0.71 (-0.99 to -0.42) |
| Guatemala | 847.1 (808.7-888.6) | 20.6 (19.6-21.7) | 2262 (1941.8-2613.8) | 19.2 (16.5-22.1) | -0.74 (-1.12 to -0.37) |
| Honduras | 176.9 (139.7-217) | 7.3 (5.6-8.9) | 693.2 (491-945.1) | 10.2 (7.2-13.9) | 1.09 (0.92 to 1.25) |
| Mexico | 8879.2 (8595.7-9172.5) | 18.7 (18.1-19.3) | 28243.3 (25014.3-31948.6) | 21.7 (19.2-24.5) | 0.42 (0.27 to 0.57) |
| Nicaragua | 219.1 (186.7-264.7) | 12 (10.1-14.4) | 706.2 (533-886.1) | 13.6 (10.2-17) | 0.66 (0.5 to 0.82) |
| Panama | 297.9 (263.5-335) | 17.1 (15.1-19.1) | 754 (591.1-938.6) | 17.1 (13.4-21.3) | -0.05 (-0.36 to 0.26) |
| Venezuela (Bolivarian Republic of) | 1511 (1421.7-1613.4) | 14.1 (13.1-15) | 5210 (3929.3-6793) | 17.2 (13-22.4) | 0.42 (0.27 to 0.58) |
| El Salvador | 682.3 (530.8-757.6) | 21.2 (16.3-23.6) | 1290.4 (1004.5-1605.4) | 21.1 (16.3-26.2) | -0.02 (-0.22 to 0.18) |
| Andean Latin America | 5273.8 (4419.1-6186.5) | 23 (19.3-27) | 16689.8 (13207.7-20766.9) | 27.5 (21.7-34.3) | 0.54 (0.43 to 0.65) |
| Bolivia (Plurinational State of) | 1426.5 (937-1920.5) | 39.4 (25.3-53.6) | 3695.5 (2426.9-5128.5) | 38.4 (25.3-53.3) | -0.15 (-0.19 to -0.11) |
| Ecuador | 1019.2 (940.2-1108.4) | 17 (15.6-18.6) | 5195.4 (4037.5-6571.7) | 31.1 (24.2-39.4) | 2.14 (1.64 to 2.65) |
| Peru | 2828 (2290.4-3466.8) | 21.3 (17-25.9) | 7798.8 (5516.8-10575.1) | 22.8 (16-30.9) | 0.03 (-0.17 to 0.22) |
| Caribbean | 4116.6 (3769-4556.3) | 14.9 (13.7-16.5) | 8690.2 (7532.9-10099.5) | 16.3 (14.1-19) | 0.46 (0.25 to 0.68) |
| Antigua and Barbuda | 8.2 (7.5-8.9) | 15.6 (14.2-16.9) | 21 (19.5-22.4) | 19.3 (18-20.5) | 0.87 (0.4 to 1.33) |
| Barbados | 43.2 (40.2-46.5) | 15.9 (14.8-17.1) | 80.7 (63.4-100.5) | 16.7 (13.2-20.9) | 0.45 (0.25 to 0.65) |
| Belize | 7.3 (6.9-8) | 7 (6.6-7.6) | 37.1 (32.6-41.8) | 10.8 (9.5-12.2) | 1.49 (1.26 to 1.72) |
| Bermuda | 13.6 (12.3-15) | 21.4 (19.4-23.6) | 20.9 (17.1-26.2) | 17.4 (14.2-21.8) | -0.68 (-1.15 to -0.2) |
| Bahamas | 33.8 (30.6-37) | 19 (17.2-20.8) | 91.6 (73.4-115) | 21.2 (17.1-26.5) | 0.78 (0.45 to 1.12) |
| Cuba | 1340 (1233.6-1449.3) | 12.9 (11.8-13.9) | 3155.8 (2662.9-3717) | 17.2 (14.4-20.2) | 1.01 (0.62 to 1.4) |
| Dominica | 8.9 (7.5-10.8) | 15 (12.6-18.1) | 12.8 (10.1-16) | 15.5 (12.3-19.3) | 0.3 (0.18 to 0.42) |
| Dominican Republic | 538.2 (438.8-633.9) | 12.3 (9.9-14.5) | 1401 (1057.7-1823.3) | 13.4 (10.1-17.5) | 0.63 (0.5 to 0.76) |
| Grenada | 16.3 (14.3-18.4) | 23.7 (20.6-26.7) | 25.3 (21.6-29.9) | 21.9 (18.7-25.7) | 0.28 (-0.13 to 0.7) |
| Guyana | 69.1 (60.5-77.9) | 15.6 (13.7-17.5) | 132.8 (99.1-174.9) | 18.8 (14.1-24.6) | 1.01 (0.82 to 1.19) |
| Haiti | 886.3 (588.6-1232.8) | 23.1 (16-31.9) | 1711.1 (1179.3-2385.5) | 19.8 (13.9-27.2) | -0.32 (-0.39 to -0.25) |
| Jamaica | 218.7 (197-241.5) | 12.3 (11-13.5) | 523.3 (391.4-688.6) | 17 (12.7-22.3) | 1.08 (0.72 to 1.45) |
| Puerto Rico | 539.3 (493.5-586.9) | 15 (13.7-16.3) | 668.1 (545.2-810.8) | 11.8 (9.6-14.5) | -0.48 (-0.78 to -0.17) |
| Saint Kitts and Nevis | 6.1 (5.6-6.7) | 17.2 (15.6-18.7) | 10.4 (8.5-12.6) | 14.3 (11.8-17.2) | -0.3 (-0.69 to 0.11) |
| Saint Lucia | 21.6 (20.1-23.2) | 23.9 (22.3-25.7) | 48.7 (40.2-58.8) | 20.7 (17.1-24.9) | -0.71 (-1.26 to -0.17) |
| Saint Vincent and the Grenadines | 19 (17.5-20.5) | 25.8 (23.7-27.8) | 41.6 (36.3-48) | 29.8 (26.1-34.3) | 0.72 (0.26 to 1.19) |
| Suriname | 41.5 (33-48.7) | 14.8 (11.9-17.2) | 84.4 (62.6-108.4) | 13.1 (9.7-16.8) | -0.01 (-0.19 to 0.17) |
| Trinidad and Tobago | 157.1 (148.6-166.7) | 17.7 (16.7-18.7) | 320.4 (242.5-416) | 17.2 (13-22.3) | -0.21 (-0.45 to 0.04) |
| United States Virgin Islands | 8.9 (7-11) | 9.6 (7.7-11.9) | 9.1 (6.8-12) | 6.6 (4.9-8.7) | -0.86 (-1 to -0.73) |
| Tropical Latin America | 15021.1 (14259-15822.7) | 14.8 (14-15.6) | 32680.5 (30547-34741.4) | 12.7 (11.8-13.5) | -0.65 (-0.75 to -0.55) |
| Brazil | 14581.7 (13822.4-15388.3) | 14.7 (13.9-15.5) | 31441.6 (29326.9-33410.5) | 12.5 (11.6-13.3) | -0.69 (-0.79 to -0.58) |
| Paraguay | 439.3 (375.4-538.9) | 17.9 (15.2-21.8) | 1238.8 (917.4-1606.5) | 20.4 (15-26.3) | 0.52 (0.45 to 0.59) |
| East Asia | 116582.2 (98062.3-136720.9) | 12.2 (10.4-14.2) | 213609.2 (173066.2-262361.7) | 10.3 (8.3-12.5) | -0.56 (-0.67 to -0.46) |
| China | 110736 (92142.7-130508.8) | 12.1 (10.1-14.1) | 203324.8 (163131.4-251788.9) | 10.1 (8.1-12.4) | -0.58 (-0.69 to -0.47) |
| Democratic People's Republic of Korea | 2450.1 (1722.3-3326.1) | 13.9 (9.9-18.7) | 4350.1 (3110.4-6010.2) | 13.1 (9.5-18.1) | -0.08 (-0.15 to -0.01) |
| Taiwan (Province of China) | 3396 (3118.1-3701) | 19.3 (17.8-20.8) | 5934.2 (5142.1-6740.7) | 15.6 (13.5-17.8) | -0.13 (-0.36 to 0.1) |
| Southeast Asia | 62694.8 (51465.3-70374.5) | 21.4 (17.9-24.3) | 164547.4 (130332.7-189174.4) | 23.6 (18.8-27) | 0.27 (0.21 to 0.34) |
| Cambodia | 1431 (945-1874) | 27.2 (18.3-35.4) | 4459.1 (2843.8-6151.8) | 33.1 (21.3-45.4) | 0.49 (0.36 to 0.62) |
| Indonesia | 21967.9 (15720.3-26122.7) | 19 (13.9-22.4) | 55406.6 (36366-72736.2) | 21 (14-27.3) | 0.23 (0.11 to 0.36) |
| Lao People's Democratic Republic | 664.6 (399.4-961) | 28.2 (17.1-40.2) | 1378.1 (889.1-1922.2) | 26.4 (17.3-36.3) | -0.35 (-0.4 to -0.3) |
| Malaysia | 2436.5 (2029.2-2867.5) | 22.9 (19.1-28) | 6609.2 (5600.6-8034.4) | 22 (18.6-27.1) | -0.27 (-0.38 to -0.16) |
| Maldives | 15 (8.7-20) | 14.5 (9-19.2) | 28.7 (21.9-37) | 7.6 (5.7-9.7) | -2.43 (-2.55 to -2.3) |
| Mauritius | 118.7 (111.2-127) | 15.3 (14.4-16.4) | 165.8 (150.2-181.1) | 9 (8.2-9.8) | -1.14 (-1.94 to -0.33) |
| Myanmar | 6519.6 (4318.3-8597.9) | 24.5 (16.8-32.1) | 11496.1 (7886.3-15296.1) | 22.2 (15.4-29.5) | -0.61 (-0.74 to -0.48) |
| Philippines | 9965.7 (7533.8-12203.3) | 28.2 (21.4-35) | 30428.3 (24389.3-36937) | 33.7 (27.3-40.7) | 0.68 (0.64 to 0.71) |
| Sri Lanka | 2479.4 (1921.8-2921.7) | 21 (16.1-24.7) | 4043.6 (2524.1-5799.5) | 15 (9.4-21.4) | -1.36 (-1.57 to -1.15) |
| Seychelles | 4.8 (3.9-5.8) | 8.3 (6.8-10.1) | 8.6 (6.9-10.3) | 7.1 (5.8-8.5) | -0.55 (-0.83 to -0.27) |
| Thailand | 7827.6 (6437.3-10636.6) | 19.8 (16.1-27.1) | 17719.9 (13111-26321.3) | 17.1 (12.7-25.4) | -0.99 (-1.24 to -0.74) |
| Timor-Leste | 64.6 (40-92.1) | 18.6 (11.9-26.1) | 186.1 (119.7-266.4) | 20.3 (13-29.2) | 0.38 (0.21 to 0.54) |
| Viet Nam | 9108.7 (6838.8-12595.4) | 20.5 (15.2-29.5) | 32388 (23960.6-42160.1) | 31.1 (23.4-40.2) | 1.9 (1.69 to 2.12) |
| Oceania | 480.3 (324.9-641.5) | 13.9 (9.8-18.5) | 1173.6 (724.9-1641.6) | 13.5 (8.6-18.6) | -0.1 (-0.14 to -0.06) |
| American Samoa | 5.2 (4-6.9) | 22.6 (16.9-29.1) | 12.4 (8.3-15.9) | 25.5 (17-32.9) | 0.67 (0.4 to 0.95) |
| Cook Islands | 2.2 (1.7-2.8) | 16.5 (12.3-20.6) | 2.9 (2.2-4) | 11.8 (9-17) | -1.78 (-2.16 to -1.39) |
| Micronesia (Federated States of) | 8.3 (6.3-10.9) | 15.3 (11.4-20.1) | 13.1 (8.6-18.3) | 16.2 (10.6-22.2) | 0.14 (0.07 to 0.2) |
| Fiji | 155 (99.8-201.7) | 36.4 (23.7-47.2) | 275.3 (173.7-384) | 34.1 (21.9-46.7) | -0.36 (-0.48 to -0.24) |
| Guam | 5.4 (4.3-9.3) | 6.9 (5.6-11.5) | 19.4 (14.6-22.9) | 9.6 (7.3-11.3) | 2.01 (1.26 to 2.76) |
| Kiribati | 0.3 (0.2-0.5) | 0.7 (0.5-1.3) | 0.6 (0.4-1.1) | 0.7 (0.5-1.3) | -0.27 (-0.44 to -0.09) |
| Marshall Islands | 2.4 (2-3) | 12.6 (10.2-15.9) | 6.3 (4.2-8.9) | 15.4 (10.3-21.2) | 0.57 (0.39 to 0.76) |
| Nauru | 0.9 (0.6-1.3) | 16.8 (11.7-22.4) | 1.4 (0.8-2.1) | 21.1 (12.9-30.3) | 0.75 (0.58 to 0.93) |
| Niue | 0.3 (0.2-0.4) | 12.9 (9.6-16.7) | 0.3 (0.2-0.4) | 16.1 (10.9-20.7) | 0.34 (0.24 to 0.44) |
| Northern Mariana Islands | 1.7 (1.2-2.7) | 8.2 (6.2-11.5) | 5.5 (4.3-6.5) | 10.3 (8-12) | 1.24 (0.66 to 1.83) |
| Palau | 1.6 (1.2-2.2) | 16 (11.9-21.7) | 3.4 (2.4-4.5) | 15.6 (11.1-19.9) | -0.01 (-0.07 to 0.05) |
| Papua New Guinea | 213.7 (125-323.4) | 9.8 (6-14.7) | 652.3 (352.7-1084.8) | 10.6 (5.9-17.6) | 0.23 (0.17 to 0.29) |
| Samoa | 20.7 (15.5-29.8) | 21.2 (15.5-33) | 34.3 (25.1-48.9) | 21.5 (15.7-32.1) | -0.02 (-0.07 to 0.03) |
| Solomon Islands | 17.3 (9.5-26.3) | 10.6 (6-16) | 56.1 (35.3-82.7) | 13.7 (8.6-20.3) | 0.8 (0.72 to 0.88) |
| Tokelau | 0.2 (0.1-0.2) | 13.4 (9.5-18.8) | 0.2 (0.1-0.3) | 15.2 (10-20) | 0.11 (0 to 0.23) |
| Tonga | 5.8 (4.3-7.9) | 9.7 (7.2-13.3) | 9.6 (6-13.5) | 11.6 (7.3-16.3) | 0.54 (0.36 to 0.71) |
| Tuvalu | 1 (0.8-1.3) | 14.4 (11.1-19.2) | 1.6 (1-2.1) | 14.5 (9.7-19.2) | -0.02 (-0.1 to 0.06) |
| Vanuatu | 7.6 (5.3-10.8) | 10.5 (7.3-14.5) | 26.1 (17.4-34.5) | 13.3 (9-17.5) | 0.67 (0.59 to 0.75) |
| North Africa and Middle East | 21944.4 (18189.4-30745.7) | 11 (9.1-15.5) | 65316.2 (55454.2-76496.3) | 12.7 (10.9-14.8) | 0.76 (0.62 to 0.9) |
| Afghanistan | 1014.9 (365.6-2818.3) | 13.7 (5-38.2) | 3097.5 (1459.4-5106.2) | 22.4 (10.7-36.2) | 1.92 (1.78 to 2.06) |
| Algeria | 1496.6 (1187.2-1898) | 9.9 (7.9-13.2) | 4578.9 (3315-5894.3) | 11.6 (8.6-14.8) | 0.87 (0.71 to 1.03) |
| Bahrain | 49.2 (39.6-63.2) | 23.8 (19.4-29.7) | 191.6 (138.8-256.6) | 18.7 (13.4-24.4) | -0.77 (-0.94 to -0.6) |
| Egypt | 3140.2 (2665.6-4263.9) | 9.8 (8.2-13.8) | 8329.8 (6474.8-10542.5) | 11.7 (9.2-14.6) | 0.89 (0.73 to 1.06) |
| Iran (Islamic Republic of) | 1383.6 (1002.6-1667.6) | 4.4 (3.1-5.4) | 7843.3 (3432.7-9779.9) | 9.3 (4.1-11.5) | 3.8 (3.19 to 4.41) |
| Iraq | 1415.3 (1039.5-2152.6) | 14.2 (10.3-22) | 4747.9 (3482.9-6435.1) | 16.4 (12-21.6) | 0.69 (0.52 to 0.87) |
| Jordan | 277 (216.5-363.5) | 15.6 (12.4-20.6) | 1171.3 (850.1-1611.9) | 13.1 (9.8-17.8) | -0.53 (-0.8 to -0.26) |
| Kuwait | 138.1 (121.5-158) | 14.6 (13-16.5) | 497.7 (399.5-621.1) | 12.8 (10.4-15.7) | 0.28 (-0.62 to 1.19) |
| Lebanon | 326.9 (246.2-429.7) | 13.9 (10.5-18.2) | 695.8 (551.2-915.5) | 11.6 (9.2-15.3) | -0.33 (-0.48 to -0.18) |
| Libya | 368.7 (275-494.7) | 15.5 (11.4-21.6) | 1213.6 (865.6-1658.1) | 18.8 (13.8-25.1) | 1.04 (0.86 to 1.22) |
| Morocco | 1387.1 (1106.1-1730.2) | 8.2 (6.6-10) | 3472.1 (2535.7-4659.6) | 9.5 (7-12.6) | 0.61 (0.54 to 0.68) |
| Palestine | 179.2 (119.4-243.4) | 18.7 (12.1-25.4) | 452.6 (285.5-561.2) | 15.8 (9.8-19.6) | -0.39 (-0.53 to -0.25) |
| Oman | 67.1 (48.3-89.6) | 7.4 (5.4-9.8) | 240.2 (160.2-315) | 8.4 (5.8-10.7) | 1.08 (0.81 to 1.35) |
| Qatar | 35.2 (26.4-44.6) | 23.1 (17.7-28.3) | 255.9 (173.1-361.8) | 18.1 (13-24.3) | -0.43 (-0.75 to -0.11) |
| Saudi Arabia | 1168.2 (806.7-1809.3) | 14.3 (10.1-23.6) | 6725.7 (4968.7-8948) | 21.6 (16.6-27.4) | 1.67 (1.43 to 1.9) |
| Sudan | 883.5 (385.2-2237.9) | 7.7 (3.5-19.2) | 2658.8 (1707.3-4050.3) | 10.3 (6.8-15.3) | 1.24 (1.08 to 1.41) |
| Syrian Arab Republic | 406.1 (226.1-945.4) | 6.2 (3.5-14.3) | 1392.4 (927.8-2093.2) | 9.9 (6.7-14.9) | 1.65 (1.55 to 1.75) |
| Tunisia | 559.3 (427.7-722.4) | 9.7 (7.4-12.6) | 1429.3 (997-1963.9) | 10.7 (7.5-14.5) | 0.36 (0.29 to 0.44) |
| Turkey | 7070 (5523-9241.8) | 18.2 (14.4-23.5) | 13413.8 (10490.5-17275) | 14.1 (11.1-18.3) | -0.82 (-0.98 to -0.67) |
| United Arab Emirates | 191.6 (132.4-266.9) | 31.4 (21.3-43.6) | 1117.4 (843.9-1470.1) | 25.4 (20-32.3) | 0.94 (0.44 to 1.44) |
| Yemen | 374.7 (204.9-838.9) | 6.3 (3.4-14.5) | 1729.6 (1181.1-2456.5) | 9.8 (6.6-14.2) | 1.78 (1.58 to 1.99) |
| South Asia | 105302.9 (88400.4-135793.4) | 14.2 (12-18.2) | 302256.8 (249827.8-356789.4) | 18.2 (15.1-21.4) | 0.85 (0.81 to 0.88) |
| Bangladesh | 9753.4 (7403.4-13331.4) | 15.1 (11.7-20.4) | 22091.5 (13982-37366.4) | 14.5 (9.3-24) | -0.01 (-0.09 to 0.08) |
| Bhutan | 54.2 (34.6-75.4) | 15.5 (10.3-22.2) | 111.3 (72.9-173.1) | 16.3 (10.9-24.8) | -0.03 (-0.1 to 0.04) |
| India | 77650.2 (64371.2-102706.5) | 13 (10.9-17.1) | 225287.8 (186616.9-264478.5) | 17.2 (14.2-20) | 1 (0.94 to 1.05) |
| Nepal | 1744.3 (1215.5-2469.5) | 14.2 (10.1-20.2) | 4464.6 (3038-6631.4) | 16.9 (11.7-24.6) | 0.72 (0.53 to 0.91) |
| Pakistan | 16100.7 (12901.9-20531) | 22.9 (18.8-29) | 50301.6 (36508.7-67210.1) | 30.2 (22.6-39.6) | 0.63 (0.39 to 0.86) |
| Southern Sub-Saharan Africa | 4035.3 (3398.8-4776.4) | 12.4 (10.3-14.8) | 10253.2 (8481-11926) | 15.7 (12.8-18.1) | 1 (0.72 to 1.27) |
| Botswana | 84.2 (55.3-130.8) | 12.6 (8.4-19.4) | 196.2 (124.1-309.6) | 11.5 (7.6-17.6) | 0.02 (-0.26 to 0.31) |
| Lesotho | 94.9 (65.9-147.4) | 10.3 (7.2-16.2) | 215.4 (146.2-320) | 17.7 (12.2-26.1) | 2.58 (2.14 to 3.02) |
| Namibia | 78.1 (60.7-99.7) | 9.8 (7.6-12.8) | 216.4 (147.4-304.1) | 12.9 (9.1-17.7) | 0.91 (0.75 to 1.07) |
| South Africa | 2554.5 (2150.2-3097.4) | 10.3 (8.5-12.7) | 5940.5 (5046.4-6946.9) | 11.9 (9.9-13.6) | 0.55 (0.36 to 0.73) |
| Eswatini | 54.9 (40.9-76.4) | 15.4 (11.6-21.4) | 132.5 (82.1-196.3) | 19.6 (12.5-28.9) | 0.97 (0.55 to 1.39) |
| Zimbabwe | 1168.7 (892-1464.4) | 23.9 (18.7-29.8) | 3552.1 (2371.9-4763.1) | 38.9 (27.4-50.7) | 2.29 (1.69 to 2.89) |
| Western Sub-Saharan Africa | 3628.7 (2761.6-4379.4) | 3.2 (2.4-3.8) | 7951.2 (6109.3-10289) | 2.8 (2.2-3.5) | -0.49 (-0.56 to -0.42) |
| Benin | 107.9 (62.9-151.3) | 4.2 (2.4-5.9) | 218.5 (144-319.7) | 2.9 (2-4.1) | -1.63 (-1.81 to -1.46) |
| Burkina Faso | 260.8 (137.3-362) | 4.9 (2.5-6.9) | 430.4 (272.2-597.3) | 3.3 (2.2-4.5) | -1.58 (-1.77 to -1.4) |
| Cameroon | 321.5 (178.3-425) | 5.6 (3.1-7.3) | 714.6 (473.1-1035.1) | 3.9 (2.6-5.5) | -1.54 (-1.69 to -1.39) |
| Cabo Verde | 2.4 (1.9-3.3) | 1 (0.8-1.4) | 20.4 (4.1-40.6) | 3.8 (0.8-7.5) | 4.4 (3.62 to 5.18) |
| Chad | 127.9 (72.6-179.8) | 3.8 (2.1-5.3) | 287.8 (202.6-396.8) | 3.1 (2.2-4.1) | -0.9 (-1.07 to -0.72) |
| Gambia | 20.6 (15.1-27.5) | 4.2 (3.1-5.5) | 68.8 (47.5-94.4) | 5.1 (3.6-6.9) | 0.37 (0.16 to 0.58) |
| Ghana | 112.8 (55.7-170.3) | 1.2 (0.6-1.7) | 301.8 (184.5-459) | 1.3 (0.8-1.9) | 0.21 (0.11 to 0.32) |
| Guinea | 308.5 (242.1-393.3) | 7.9 (6.3-10) | 625.1 (438.3-892.9) | 8.1 (5.8-11.2) | 0.06 (0.02 to 0.1) |
| Guinea-Bissau | 34.8 (19.4-48.8) | 6.4 (3.4-8.9) | 52.2 (37.6-72.8) | 4.5 (3.2-6) | -1.43 (-1.56 to -1.29) |
| Liberia | 65.7 (34.6-89.1) | 4.6 (2.3-6.3) | 112.7 (73.1-160.4) | 3.4 (2.3-4.7) | -1.24 (-1.4 to -1.07) |
| Mali | 799.4 (641.4-968.6) | 15.7 (12.7-18.9) | 1657.3 (1137.6-2415.7) | 13.2 (9.3-18.9) | -0.58 (-0.67 to -0.48) |
| Mauritania | 57.3 (31.8-76.3) | 4.9 (2.6-6.5) | 83.7 (57.6-120.2) | 3.1 (2.1-4.5) | -1.94 (-2.1 to -1.78) |
| Niger | 159.5 (89.2-217.6) | 4 (2.1-5.4) | 323.9 (203.4-472.2) | 2.6 (1.7-3.7) | -1.78 (-1.95 to -1.6) |
| Nigeria | 371.9 (242.3-669.7) | 0.7 (0.4-1.2) | 1006.2 (646-1797.1) | 0.8 (0.5-1.4) | 0.59 (0.47 to 0.71) |
| Sao Tome and Principe | 2.3 (0.7-4.4) | 2 (0.8-3.3) | 3.7 (1.5-6.6) | 2 (1-3.3) | 0.24 (0.15 to 0.34) |
| Senegal | 179.9 (95.7-265.4) | 4.3 (2.2-6.2) | 315.1 (209.6-475.2) | 3.1 (2.1-4.6) | -1.32 (-1.45 to -1.19) |
| Sierra Leone | 93.3 (47-131.6) | 3.7 (1.8-5.3) | 161 (98.4-237.5) | 2.9 (1.9-4.2) | -0.93 (-1.07 to -0.78) |
| Togo | 80.2 (44.5-115.9) | 4.5 (2.3-6.3) | 177.3 (113.1-270.9) | 3.3 (2.2-5) | -1.24 (-1.36 to -1.12) |
| Cmte d'Ivoire | 521.8 (381.1-672.1) | 8.6 (6.5-10.8) | 1390.8 (962.3-1981) | 8.3 (5.9-11.5) | 0.01 (-0.08 to 0.1) |
| Eastern Sub-Saharan Africa | 31046 (25044.5-38047.3) | 30.1 (24.5-36.7) | 66490.9 (48520.2-94804.4) | 27.8 (20.6-38.4) | -0.43 (-0.55 to -0.32) |
| Burundi | 808.9 (578.8-1134.9) | 26 (18.6-35.9) | 1440.5 (922.5-2229.2) | 19.9 (13-29.5) | -1.22 (-1.4 to -1.05) |
| Comoros | 53.8 (33.9-82.2) | 20.2 (13.7-29.5) | 129 (84.6-204.7) | 21.9 (14.6-34.3) | 0.08 (-0.12 to 0.28) |
| Djibouti | 34.8 (22.7-52.3) | 16.8 (11.5-24.6) | 174.3 (104.6-292.1) | 20.2 (12.9-32.1) | 0.66 (0.58 to 0.74) |
| Eritrea | 385.1 (310.6-493.4) | 22.9 (19-28.9) | 992.6 (660.7-1518.6) | 25.7 (17.9-37) | 0.5 (0.44 to 0.55) |
| Ethiopia | 17175 (12842.7-23141.3) | 62.9 (47.6-83.4) | 27067.5 (18221.8-42991.6) | 44.2 (29.8-67.4) | -1.54 (-1.73 to -1.34) |
| Kenya | 597.3 (404.5-889.3) | 5.2 (3.6-7.8) | 2343.1 (1703-3530.6) | 7.7 (5.7-11.3) | 1.78 (1.55 to 2.02) |
| Madagascar | 1258.2 (1006.1-1595) | 18.2 (14.6-22.9) | 3180.8 (2163.2-4358.1) | 18.5 (12.7-25.3) | 0.13 (-0.01 to 0.27) |
| Malawi | 960.6 (716-1263.1) | 17.4 (13.2-22.1) | 2325.5 (1483.7-3628.8) | 21.4 (14.2-31) | 0.73 (0.53 to 0.92) |
| Mozambique | 1439 (972.9-2247.6) | 18.4 (12.8-27.8) | 4050.9 (2550.8-7148.6) | 24.8 (16.1-41.7) | 1.42 (1.24 to 1.59) |
| Rwanda | 1252.2 (952-1658.3) | 31 (23.7-41.2) | 1899.8 (1295.1-2834.2) | 22.5 (15.8-32.3) | -1.7 (-1.97 to -1.43) |
| Somalia | 764.6 (507.1-1073.5) | 20.7 (13.9-28.9) | 2148.1 (1383.6-3069) | 22.5 (14.8-31.6) | 0.36 (0.31 to 0.41) |
| South Sudan | 557.9 (387.9-795.9) | 17.1 (12.2-24) | 1208.2 (818.6-1791.5) | 22.4 (15.4-32.3) | 0.88 (0.64 to 1.13) |
| United Republic of Tanzania | 2964.4 (2182.9-4094) | 20.6 (15.4-28) | 7527.2 (5070.6-10971) | 21.7 (15.2-30.7) | 0.22 (0.12 to 0.32) |
| Uganda | 1851.1 (1343.9-2515) | 21.9 (16.1-29.5) | 8249.7 (5658.4-11488.6) | 37.6 (26.7-50.8) | 1.6 (1.39 to 1.82) |
| Zambia | 921.1 (741.2-1147.3) | 21.8 (17.6-26.9) | 3695.9 (1725.2-7110.8) | 34.1 (17.3-61) | 1.62 (1.29 to 1.96) |
| Central Sub-Saharan Africa | 2545.3 (1889.3-3609.5) | 9.4 (6.9-13.7) | 5956.3 (3862.9-9187.8) | 8.9 (5.8-13.9) | -0.19 (-0.31 to -0.06) |
| Angola | 438.9 (312.9-613.1) | 8.9 (6.2-12.8) | 1362.6 (835.3-2151.1) | 9.2 (5.6-14.5) | 0.18 (0.09 to 0.28) |
| Central African Republic | 160.3 (119.4-228) | 11.6 (8.8-16.3) | 274.3 (180.7-411.9) | 9.9 (6.6-14.7) | -0.59 (-0.64 to -0.53) |
| Congo | 155.3 (116.6-219.3) | 12.7 (9.4-17.5) | 377.4 (237-569.8) | 11.4 (7.4-16.9) | -0.49 (-0.62 to -0.36) |
| Democratic Republic of the Congo | 1688.8 (1187.2-2524.1) | 9 (6.3-13.8) | 3735.3 (2319.9-6050.6) | 8.5 (5.1-13.9) | -0.21 (-0.37 to -0.04) |
| Equatorial Guinea | 23.1 (16.3-33.2) | 10.2 (7.2-14.8) | 69.9 (38.7-113.2) | 10.5 (6-16.6) | 0.16 (-0.01 to 0.34) |
| Gabon | 79 (59.5-107.2) | 12.9 (9.7-17.5) | 136.9 (93.9-199.3) | 11.5 (8-16.5) | -0.57 (-0.67 to -0.46) |


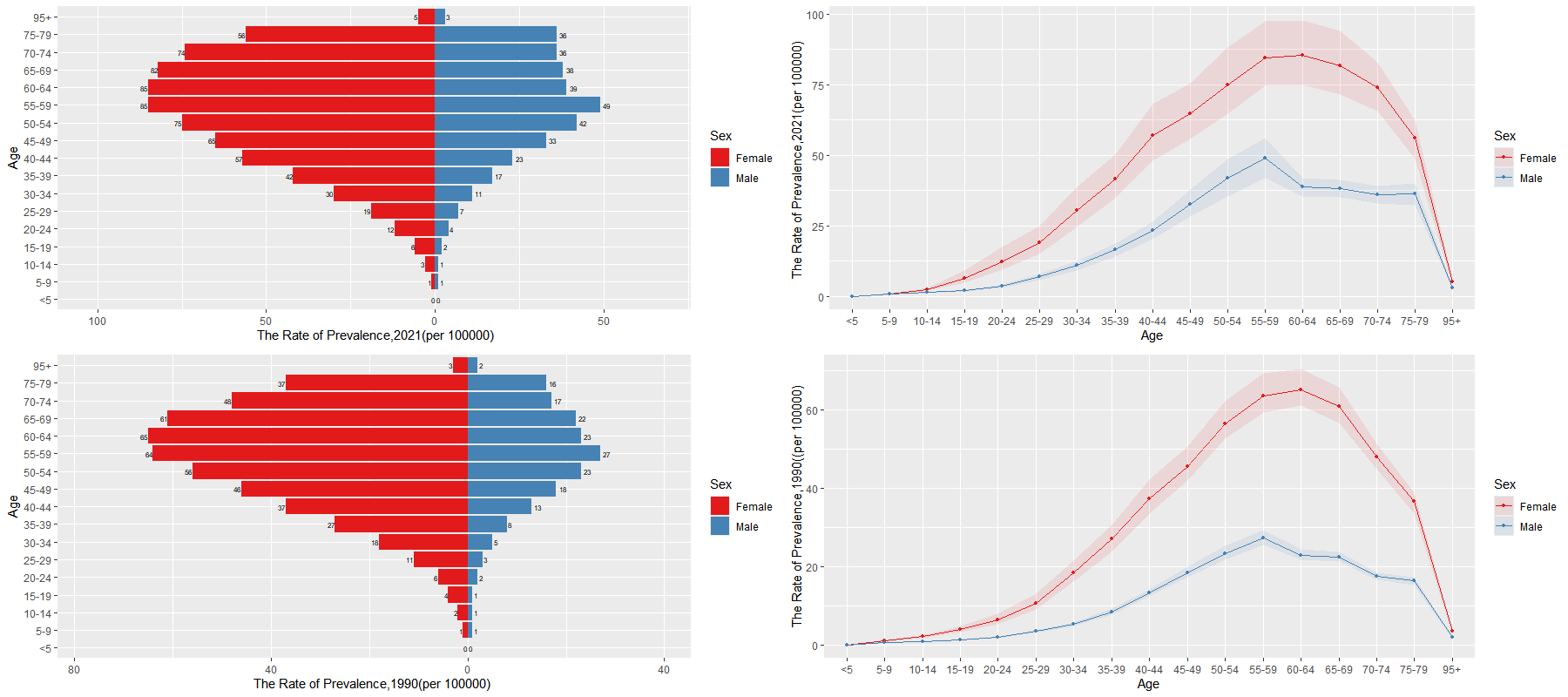


Figure S1 Temporal trends in age- and sex-stratified prevalence rates (per 100,000 population) for thyroid cancer , comparing 1990 and 2021.


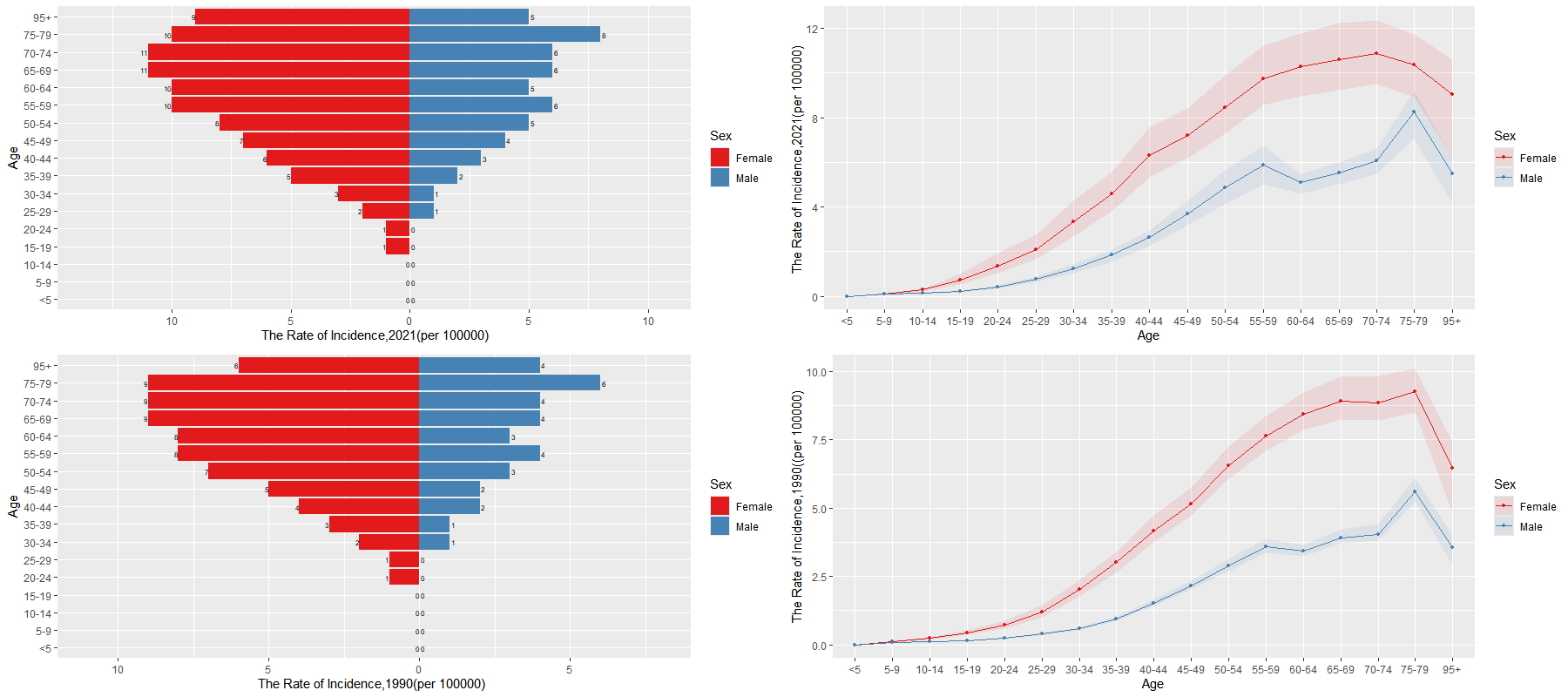


Figure S2 Temporal trends in age- and sex-stratified incidence rates (per 100,000 population) for thyroid cancer , comparing 1990 and 2021.


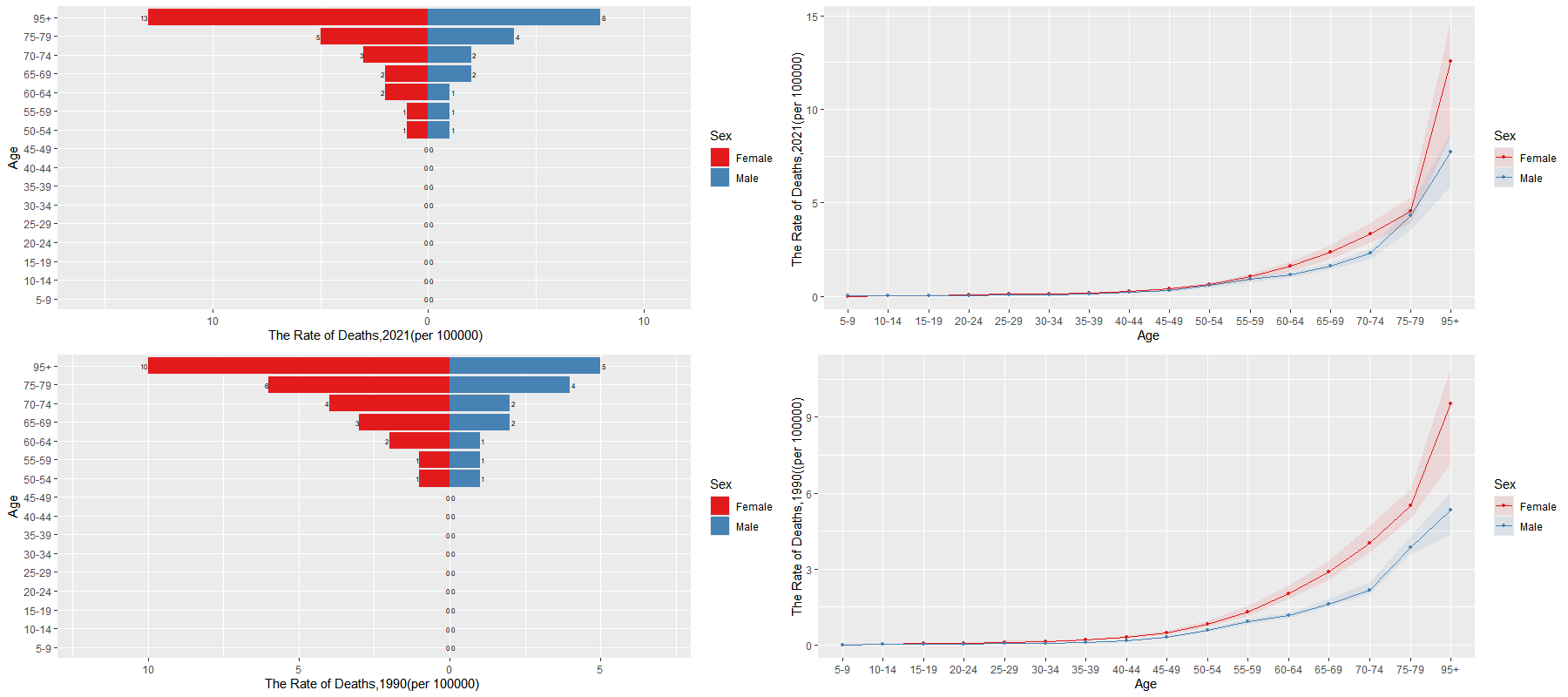


Figure S3 Temporal trends in age- and sex-stratified deaths rates (per 100,000 population) for thyroid cancer , comparing 1990 and 2021.


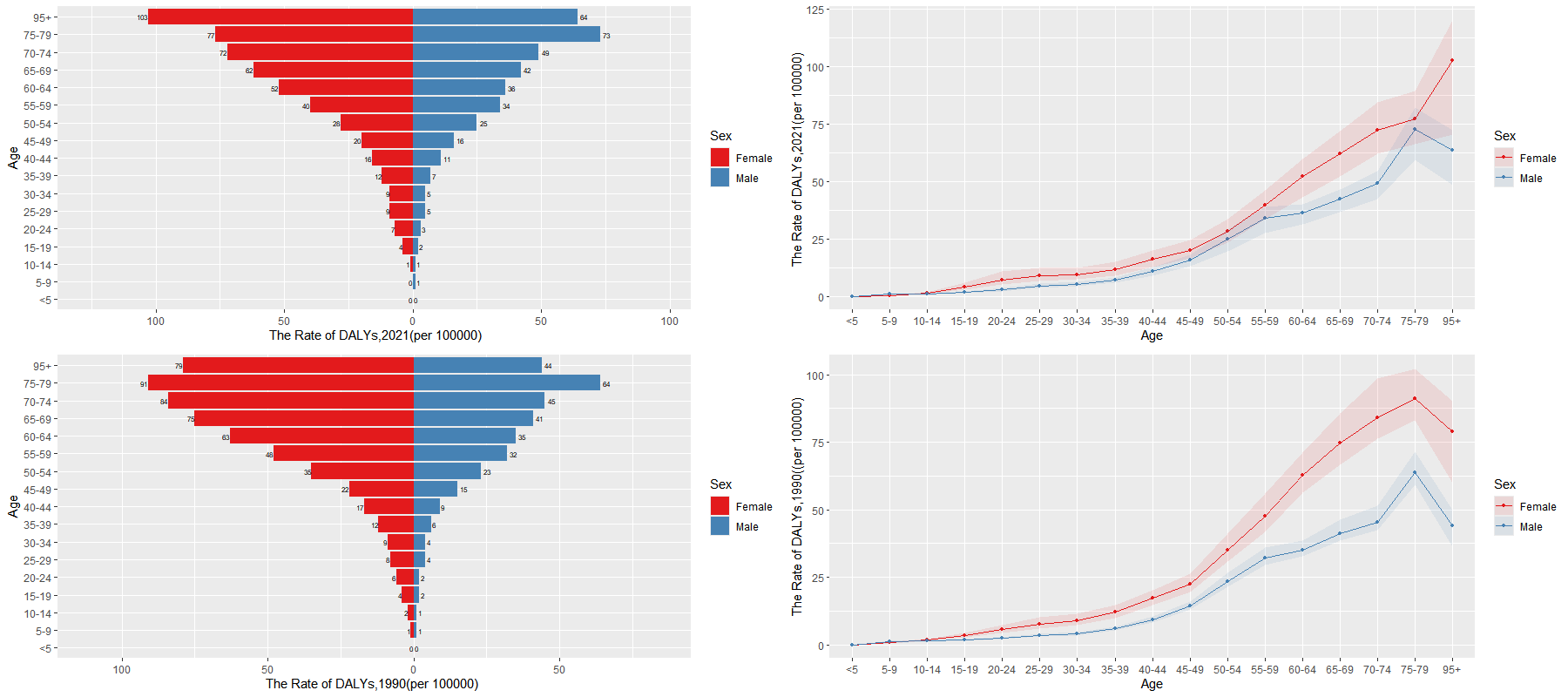


Figure S4 Temporal trends in age- and sex-stratified DALY rates (per 100,000 population) for thyroid cancer , comparing 1990 and 2021.

### ****Methods S1: Detailed implementation of the BAPC model.****

### ****Foundation and Construction of the BAPC Model****

#### ****1. 1 Data Preparation and Structure****

**Data Sources**: Age-specific disease incidence/mortality rates and population denominators; **Structure**: Data organized into a three-dimensional table by: **Age groups** (e.g., 5-year intervals); **Calendar periods** (e.g., 5-year intervals); **Birth cohorts** (derived as period minus age)

#### ****1.2 Mathematical Formulation:****

The core is a Poisson regression framework:

log(λ_{apc}) = μ + α_a + β_p + γ_c + ε

where:

λ_{apc}: Disease rate for age group"a", period" p", and cohort"c" ; α_a: Age effect (baseline age-specific risk); β_p: Period effect (impact of external interventions); γ_c: Cohort effect (lifetime exposure differences by birth cohort)

#### ****1.3 Core Implementation Steps****

**Trend Decomposition**: Use **Intrinsic Estimator (IE)** or **penalized least squares** to resolve linear dependency (non-identifiability).

**Prior Distribution Specification**: Age effect: Random Walk (RW1/RW2) prior to model smooth risk progression; Period/cohort effects: First- or second-order Random Walk priors to constrain temporal fluctuations.

**Bayesian Inference**: Employ **Markov Chain Monte Carlo (MCMC)** or **Integrated Nested Laplace Approximation (INLA)** for posterior sampling.

### ****Prior Specification: Rationale and Assumptions****

#### ****2.1 Basis for Prior Selection****

**Random Walk (RW) Priors**: Enforce smoothness in adjacent age/period/cohort effects.

Rationale: Chronic diseases typically exhibit monotonic or unimodal age-risk patterns.

**Weakly Informative Priors**: e.g., Gaussian distribution N(0, σ²) with large variance (e.g., σ=100) to minimize subjectivity.

#### ****2.2 Key Assumptions****

**Linear Additivity**: Independent and additive age, period, and cohort effects (testable via interaction terms).

**Effect Smoothness**: Continuity of risk across adjacent groups (adjust prior variance if data noise is high).

**Temporal Extrapolation**: Future period/cohort effects follow historical trends (requires sensitivity analysis).

Table: Recommended Priors for BAPC Components

| **Effect** | **Recommended Prior** | **Typical Use Case** | **Key Risk** |
| --- | --- | --- | --- |
| Age | RW1 / RW2 | Chronic diseases | Overfitting in sparse old-age groups |
| Period | AR(1) or RW1 | Acute epidemics | Trend breaks due to interventions |
| Cohort | RW2 | Long-term exposure factors | Bias from early cohort data gaps |

### ****Model Validation and Uncertainty Quantification****

#### ****3.1 Internal Validation****

**Goodness-of-fit**:

Compare models using **Deviance Information Criterion (DIC)** or **Watanabe-Akaike Information Criterion (WAIC)**.

**Residual Analysis**:

Assess spatial autocorrelation (e.g., Moran’s I) of standardized residuals.

#### ****3.2 External Validation****

**Back-testing**:

Train on partial historical data, predict holdout periods, and compare with observed values.

**Cross-population Validation**:

Test generalizability in populations with similar sociodemographic profiles.

#### ****3.3 Uncertainty Handling****

**Prediction Intervals (PI)**:

Generate 95% PI from posterior predictive distributions.

**Sensitivity Analyses**:

Test alternative priors (e.g., RW2 vs. AR1).

Vary extrapolation functions for period/cohort effects (linear vs. polynomial).

### ****Prediction Implementation****

#### **Key Steps**

**Parameter Stabilization**: Fix age effects (assuming biological stability); extrapolate period and cohort effects.

**Bayesian Projection**:Simulate future β_p and γ_c from posteriors to compute λ_{apc}.

**Result Synthesis**:Combine with population projections to estimate absolute case numbers.

### ****Methods S2: Estimation of EAPC and sensitivity analyses****

**1.Estimation of EAPC and Confidence Interval Construction**

We employed log-linear regression models to estimate the Estimated Annual Percentage Change (EAPC), quantifying the temporal trend in disease rates. The methodology proceeded as follows:

**1.1 Model Specification:**

Let *t* denote the observation year(*t*=1,2,3...,*T*),with Y*_t_* representing event counts (e.g., incidence, mortality) and P*t* the corresponding population at risk. We specified a generalized linear model:

log(*λt*)=log(*Pt*)+*β*0+*β*_1_*t*

Where**:**

*λt*=*E*(*Yt*) is the expected event count,

log(P*t*)is the **offset term** adjusting for population size heterogeneity,

*β*_1_ is the temporal slope coefficient.
The response *Yt* was assumed to follow a Poisson(λt) distribution. When overdispersion was detected (variance > mean), a negative binomial distribution was substituted.

**1.2 EAPC Calculation:**

EAPC=(*e^β^*^1^​−1)×100

A positive *β*1 indicates an increasing trend; a negative *β*1 indicates a decreasing trend.

**1.3 Confidence Interval Derivation:**

Maximum likelihood estimation (MLE) provided (
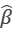
1) and its standard error SE(
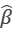
1).

Utilizing asymptotic normality of  (
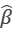
1) , the 100(1−α)%CI for *β*_1_was computed as:

CI(*β*_1_)=
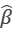
1 ± *Z_1−α/2_* × SE(
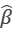
1)

The CI was transformed to the EAPC scale:

CI(EAPC)=[(e^L^*^β1^*−1)×100%,(e^U^*^β1^*−1)×100%]

where *L_β_*_1_and *U_β_*_1_ represent the lower and upper limits of *β*1's CI, respectively.

1. **Confidence Interval Derivation:**

To evaluate the robustness of modeling assumptions, we conducted the following sensitivity analyses:

**2.1 Assessment of Overdispersion**:

Compared EAPC estimates and CIs between Poisson and negative binomial models.

Tested for significant overdispersion using likelihood ratio tests (LRT) or by evaluating the dispersion parameter's significance.

Results were considered sensitive if:
(a) Negative binomial regression indicated significant overdispersion (p<0.05), and
(b) The EAPC CI width changed by >20% or statistical significance (e.g., inclusion of null value) was altered.

**2.2 Evaluation of Linearity Assumption**:

Extended the base model with a quadratic term:

log(λ_t_)=log(P_t_)+*β*0+*β*_1_t+*β*_2_t^2^

Significance of *β*2 was assessed via Wald test (p<0.05).

If *β*2 was significant, **Joinpoint Regression** (Joinpoint Regression Program, Version 5.0, NCI) was implemented to identify structural breakpoints, and segment-specific EAPCs were reported.

**2.3 Sensitivity to Temporal Coding**:

Re-estimated models using alternative time codings:
(a) Raw year (*t*=year)
(b) Centered year (*t*=year−median(year))
(c) Standardized year (*t*=year−*μ*_year_/*σ*_year_)

Variations in 
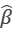
1 >10% were flagged as notable.

**2.4 Adjustment for Potential Confounders**:

Incorporated covariates (e.g., age groups, sex) into the model:

log(λ_t_)=log(P_t_)+*β*0+*β*_1_t+*
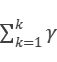
k X_K_*

Sensitivity was declared if covariate adjustment changed 
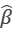
1 by >10% or modified statistical inference conclusions.

**2.5 Residual Diagnostics**:

Examined independence and homoscedasticity assumptions using Pearson residual-vs-fitted value plots.
